# The co-occurrence of overweight/obesity and anaemia among adult women, adolescent girls and children living in fifty-two low- and middle-income countries

**DOI:** 10.1017/S1368980021002512

**Published:** 2022-06

**Authors:** Ana Irache, Paramjit Gill, Rishi Caleyachetty

**Affiliations:** 1 Warwick Centre for Global Health, Warwick Medical School, University of Warwick, Medical School Building, Coventry CV4 7HL, UK; 2 Nuffield Department of Medicine, University of Oxford, Oxford, UK

**Keywords:** Overweight, Obesity, Anaemia, Women, Adolescent girls, Children, Low- and middle-income countries

## Abstract

**Objective:**

To investigate the magnitude and distribution of concurrent overweight/obesity and anaemia among adult women, adolescent girls and children living in low- and middle-income countries (LMIC).

**Design:**

We selected the most recent Demographic and Health Surveys with anthropometric and Hb level measures. Prevalence estimates and 95 % CI of concurrent overweight/obesity and anaemia were calculated for every country, overall and stratified by household wealth quintile, education level, area of residence and sex (for children only). Regional and overall pooled prevalences were estimated using a random-effects model. We measured gaps, expressed in percentage points, to display inequalities in the distribution of the double burden of malnutrition (DBM).

**Setting:**

Nationally representative surveys from fifty-two LMIC.

**Participants:**

Adult women (*n* 825 769) aged 20–49 years, adolescent girls (*n* 192 631) aged 15–19 years and children (*n* 391 963) aged 6–59 months.

**Results:**

The pooled prevalence of concurrent overweight/obesity and anaemia was 12·4 % (95 % CI 11·1, 13·7) among adult women, 4·5 % (95 % CI 4·0, 5·0) among adolescent girls and 3·0 % (95 % CI 2·7, 3·3) among children. Overall, the DBM followed an inverse social gradient, with a higher prevalence among the richest quintile, most educated groups and in urban areas; however, important variations exist. The largest inequality gaps were observed among adult women in Yemen by household wealth (24·0 percentage-points) and in Niger by education level (19·6 percentage-points) and area of residence (11·9 percentage-points). Differences were predominantly significant among adult women, but less among girls and children.

**Conclusions:**

Context-specific, multifaceted, responses with an equity lens are needed to reduce all forms of malnutrition.

For women, adolescent girls and children living in low- and middle-income countries (LMIC), micronutrient deficiencies and overweight (including obesity) are common nutrition-related disorders. Globally, the prevalence of anaemia, a proxy for micronutrient deficiencies in the absence of micronutrient data^(1)^, is 32·8 % among women of reproductive age (15–49 years old) and 43 % among children under-five^(2,3)^, whereas 39·2 % of women (≥ 18 years), 17·5 % of children and adolescents girls (5–19 years old) and 5·6 % of children under-five are currently living with overweight or obesity^(2)^. These estimates indicate modest reductions in anaemia alongside rapid increases in overweight/obesity^(2–4)^, resulting in a double burden of malnutrition (DBM) with multiple health and economic consequences^(5–7)^.

Anaemia can cause physical and cognitive impairments, fatigue and low productivity^(8–10)^, while overweight/obesity has been associated with a higher risk of developing non-communicable diseases (e.g. diabetes and CVD)^(11–13)^. Maternal anaemia contributes to maternal deaths and low birth weight^(9,14)^, and maternal overweight/obesity increases maternal morbidity, preterm birth and infant mortality^(14)^.

With no country on track to meet the 2025 Global Nutrition Targets of anaemia and adult obesity^(2)^, dual burdens of malnutrition present a major opportunity for integrated action to end malnutrition in all its forms by 2030^(15)^. Quantifying the extent and distribution of the DBM is the first step to achieve this, to guide the development of context-specific programmes and policies that address the full spectrum of malnutrition and that take into account inequalities, leaving no one behind^(2,16)^.

According to a recent review^(17)^, little research has focused on the coexistence of overweight/obesity with anaemia or micronutrient deficiencies at the individual level, when compared with other forms of DBM (e.g. overweight/obesity and stunting). Moreover, anaemia data were also excluded from the most comprehensive analysis on the DBM to date^(5)^, which might have underestimated the overall magnitude of the dual burden in LMIC^(18)^. Previous epidemiological studies on the magnitude of concurrent overweight/obesity and anaemia have been conducted predominantly in individual countries and have focused on women of reproductive age^(19–26)^ or children^(21,24)^. Thus, the co-occurrence of overweight/obesity and anaemia among adolescent girls remains poorly understood. Another review including Latin American countries found the proportion of concurrent overweight/obesity and anaemia to range from 3·4 % to 13·6 % among women and from 1·2 % to 1·4 % among children under-five^(27)^. More recently, the co-occurrence of overweight/obesity and anaemia has been estimated in a larger, yet limited number of countries (LMIC and high-income countries spanning different regions) among women of reproductive age (*n* 16)^(28)^ and pre-school children (*n* 21)^(29)^. The latter DBM study showed a positive association between higher socio-economic status and concurrent overweight/obesity and anaemia among women of reproductive age living in LMIC^(28)^; however, stratified analyses by socio-demographic characteristics are missing, masking subgroups within countries who might be most vulnerable to be simultaneously affected by both forms of malnutrition. Identifying subgroups at higher risk to develop concurrent overweight/obesity and anaemia is of utmost importance in order to design appropriate interventions that reduce health inequalities^(30)^.

To address the gaps in knowledge, this study examined the magnitude and distribution of concurrent overweight/obesity and anaemia by household wealth, education level, area of residence and sex, among adult women, adolescent girls and children living in LMIC.

## Methods

### Data sources and study participants

This study was based on the most recent Demographic and Health Surveys (DHS) from all LMIC (conducted between January 2000 and January 2019) with available anthropometric data (weight and height) and Hb levels for adult women, adolescent girls and children under-five. DHS are internationally comparable, nationally representative household surveys, conducted in LMIC about every 5 years, that provide data for a wide range of monitoring and impact evaluation indicators in the areas of population, health and nutrition. Complete descriptions of country DHS sampling, validation of questionnaires, data collection and data validation are published elsewhere^(31)^. DHS follow a multistage stratified random sampling technique. In the first stage, the number of households needed per geographical areas is determined, and clusters (or census enumeration areas) are randomly selected with probability proportional to size. The second stage consists of a random selection of households within the selected clusters. For each sampled household, standard model questionnaires are then employed to collect primary data at the household and individual level. Informed consent to participate in the study is taken from the participant (or from a parent or legal guardian for children and unmarried adolescents), before conducting any questionnaire or biomarker tests. In all households, members eligible for biomarker collection include women aged 15–49 years and children under 59 months^(31)^. We excluded women and girls who were pregnant or who have given birth in the 2 months preceding data collection, due to weight gain during pregnancy, and following DHS guidelines^(31)^. Individual participants with missing anthropometric measures and data on anaemia status (missing values or data not recorded) and those with biologically implausible height, weight or Hb values were also excluded from the analytic sample (see online Supplemental Fig. S1).

Ethical approval for conducting the DHS surveys was obtained centrally by the ORC Macro Institutional Review Board and by individual review boards within the individual countries participating in the programme. The DHS data sets, both the individual (IR) and children’s (KR) recodes used for this study, are publicly available and accessible at https://dhsprogram.com.

### Measure of anthropometry and Hb levels

DHS trained personnel weighed and measured children under-five and their mothers (15–49 years old), using a SECA digital scale and Shorr Productions measuring board. For children younger than 24 months old, recumbent length was obtained. BMI was calculated by dividing body weight in kilograms by squared height in squared metres. To define overweight/obesity, we used the Quetelex index for adult women (20–49 years old)^(32)^, and the WHO 2007 growth reference data for adolescent girls (15–19 years old)^(33)^. Women were categorised as having overweight/obesity if their BMI was ≥ 25·0 kg/m^2^, whereas among adolescent girls, overweight/obesity was defined as BMI-for-age *Z*-score >1 SD above from the median of the reference population. Among children under-five, a BMI-for-age *Z*-score >2 SD was classified as overweight/obesity^(34,35)^.

Diagnosis of anaemia was confirmed by measuring Hb concentration levels in blood through a stick capillary blood sample, using HemoCue® 201+ or the 301+ system, a portable Hb analyser. Anaemia in adult women and adolescent girls aged 15–49 years old was defined as Hb concentration levels <12·0 g/dl, and <11·0 g/dl in children (6–59 months)^(36,37)^. Hb levels were adjusted for altitude and smoking (when data on smoking status were collected) in women of reproductive age, and for altitude in children, as these are known factors to increase Hb concentrations^(31)^. Similarly, Hb levels were not measured in infants aged 0–6 months, considered a low-risk group for anaemia, for presenting higher Hb concentration levels in the first 6 months of life^(31)^.

### Defining the double burden of malnutrition at the individual level

The double burden was defined as the simultaneous presence of overweight/obesity and anaemia among: (i) adult women (20–49 years old); (ii) adolescent girls (15–19 years old) and (iii) children (6–59 months).

### Covariates

DHS collect information on a wide range of socio-demographic factors. Household wealth was generated using principal component analysis with data collected at each household including assets (e.g. bicycles, cars or radios) and dwelling characteristics (e.g. flooring material, drinking water source or type of toilet facility)^(38)^ and was further divided into five quintiles (Q1: poorest; Q2: poorer; Q3: middle; Q4: richer; Q5: richest). Education level, or maternal education level for children, was assessed by self-report of the completed educational level (E1: no education; E2: primary education; E3: secondary education; E4: higher education). Among adolescent girls, the third and fourth education levels were combined into one category as not all girls might have reached higher education, and thus, avoid low sample sizes. Place of residence was defined according to country-specific definitions and categorised as urban or rural. We also included sex for children, categorised as girls or boys.

## Statistical analysis

Prevalence estimates and 95 % CI of concurrent overweight/obesity and anaemia were first calculated for every country. We also ran separate stratified analyses by wealth quintile, education level, area of residence and sex (for children only). Because some of the combinations in the stratified analysis resulted in small sample sizes, we excluded prevalence estimates for which the sample size was lower than twenty-five observations, in accordance with DHS guidelines^(31)^. Pooled prevalence estimates were then calculated (command ‘*metaprop*’) using a random-effects model, overall and by WHO region (i.e. African, Eastern Mediterranean, European, Americas, Southeast Asian and Western Pacific). The Western Pacific region only had one country with available data (Cambodia); hence, the regional pooled prevalence could not be generated.

We measured inequality gaps, defined as the absolute difference in percentage points between the prevalence of DBM among the highest and lowest household wealth quintile (Q5-Q1), the highest and lowest education level (E4/E3-E1), urban *v*. rural areas and boys *v*. girls. Positive gaps depict a higher prevalence of concurrent overweight/obesity and anaemia in the richest quintile, most educated group, urban residents and boys than in the poorest quintile, least educated, rural residents and girls, respectively. Negative gaps represent the opposite. *P*-values <0·05, obtained through *χ* ^2^ tests and tests for trend, mean that differences in the prevalence of DBM observed across the different groups were significant and not due to chance.

All analyses were conducted using Stata version V.16.0. (Statacorp.). We used sampling weights and the Stata’s survey estimation procedures (‘*svy*’ command) throughout the analyses to account for the clustering and stratification in the sample design of DHS surveys.

## Results

### Characteristics of surveys and participants

Overall, fifty-two LMIC had a DHS between 2003 and 2018 with available data on anthropometry and anaemia, comprising a total of 825 769 adult women (20–49 years old), 192 631 adolescent girls (15–19 years old) and 391 963 children (6–59 months). By WHO region, per total number of LMIC with a DHS survey from 2000 onwards (*n* 69), 31 (86·1 %) of 36 in the African region, 3 (50·0 %) of 6 in the Eastern Mediterranean region, 6 (75·0 %) of 8 in the European region, 6 (66·7 %) of 9 in the Americas region, 5 (71·4 %) of 7 in the Southeast Asian region and 1 (33·3 %) of 3 in the Western Pacific region. We found anthropometric and anaemia data missing in Angola for adult women and adolescent girls. For Madagascar and Jordan, height and weight measurements among children were deemed unreliable in the most recent DHS survey and thus, we used the second most recent survey with available data for this age group. As a result, fifty-one DHS surveys were included in the analysis for adult women and adolescent girls, and fifty-two DHS surveys for children.

**Fig. 1:**
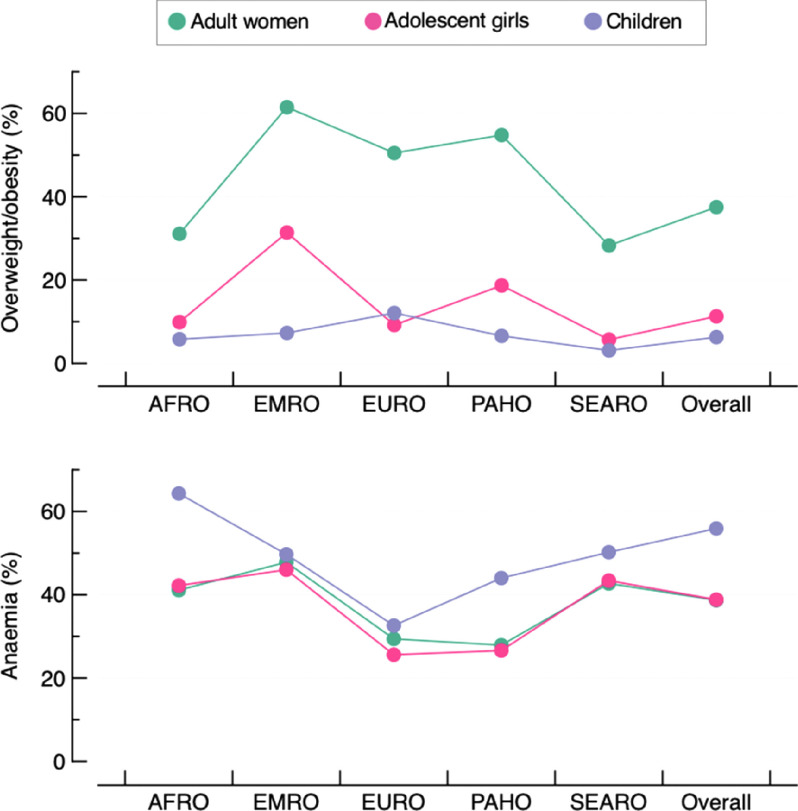
Bivariate prevalence of overweight/obesity and anaemia in the studied population by WHO regions and overall. Each dot is the pooled prevalence of overweight/obesity or anaemia. AFRO: African region; EMRO: Eastern Mediterranean region; EURO: European region; PAHO: Americas region; SEARO: Southeast Asian region. The Western Pacific region is missing as it only has one country with available data (Cambodia)

Characteristics of participants included in the study are provided in Supplemental Tables 1–3. The median age was between 30 and 43 years old among adult women, 17 and 18 years old among adolescent girls and 28 and 35 months among children. The largest sample size was found in India for the three age groups (*n* > 100 000), whereas sample sizes were smaller among adolescent girls (*n* < 1000 in 16 countries) when compared with adult women or children. The overall bivariate prevalence of overweight/obesity and anaemia was 37·5 % and 38·7 % among adult women, 11·3 % and 38·8 % among adolescent girls and 6·3 % and 55·9 % among children, respectively (Fig. 1 and see online Supplemental Table S4).

### Overweight/obesity and anaemia among adult women

**Fig. 2:**
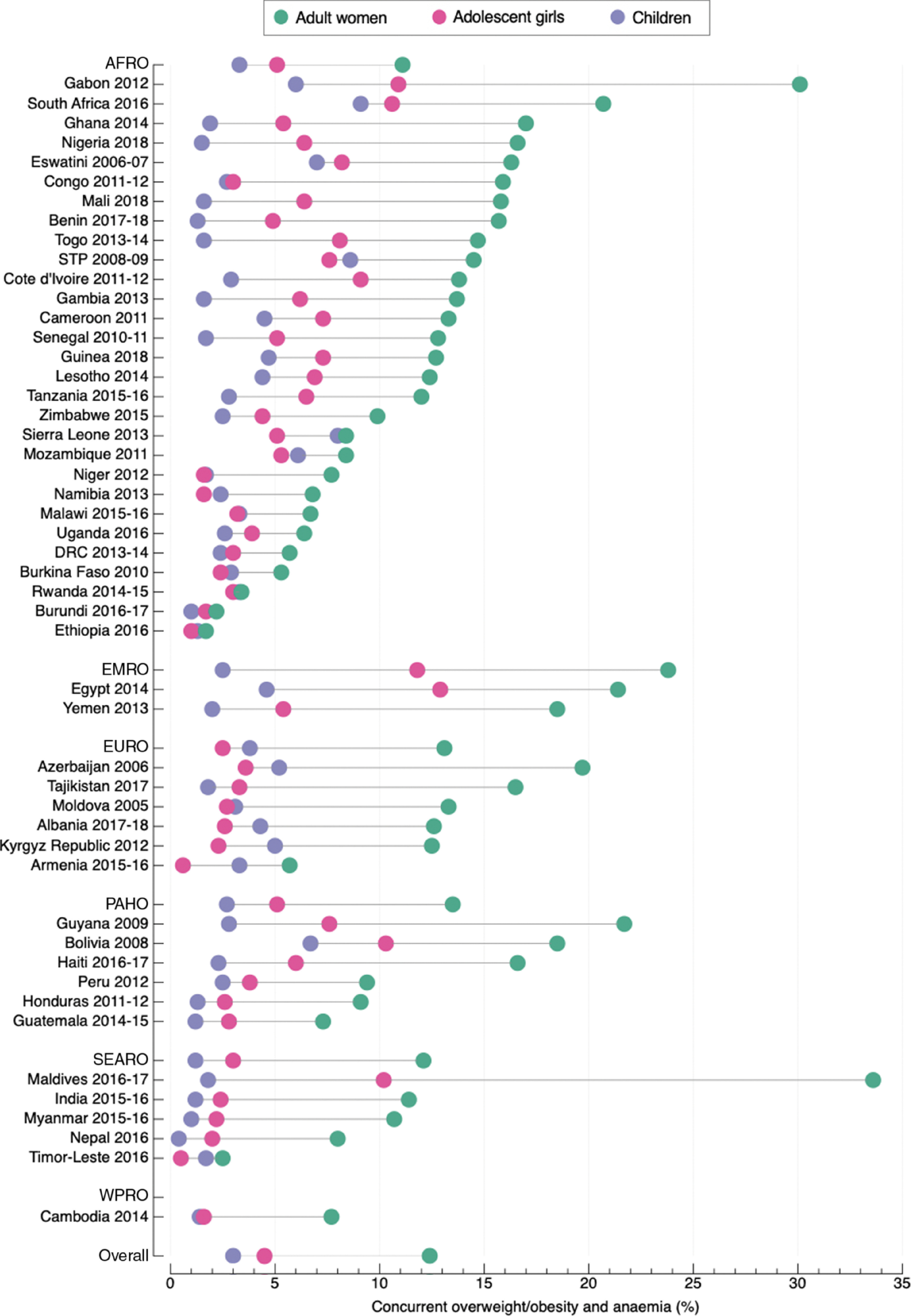
Prevalence of concurrent overweight/obesity and anaemia among adult women, adolescent girls and children living in LMIC. AFRO: African region; EMRO: Eastern Mediterranean region; EURO: European region; PAHO: Americas region; SEARO: Southeast Asian region; WPRO: Western Pacific region; DRC: Democratic of the Congo; STP: Sao Tome and Principe. Jordan and Madagascar are missing because data were from different DHS surveys. Angola was not included because data were missing for women of reproductive age. The three missing countries were included in the calculation of the regional and overall pooled prevalence

The pooled prevalence of concurrent overweight/obesity and anaemia among adult women was 12·4 % (95 % CI 11·1, 13·7; *I* ^2^ 99·6 %), ranging from 1·7 % in Ethiopia to 33·6 % in Maldives (Fig. 2 and see online Supplemental Table S4). The regional pooled prevalence ranged from 11·1 % (95 % CI 9·2, 13·0) in the African region to 23·8 % (95 % CI 17·0, 30·7) in the Eastern Mediterranean region.

The full distribution of the magnitude of the DBM among adult women is presented in Supplemental Tables 5–7. Overall, the highest DBM prevalence was found in the richest quintile (16·5 %), third education level (13·6 %) and urban women (15·3 %). The European region presented a distinct pattern for two of the three socio-economic measures, with the poorer quintile (13·9 %), third education level (16·8 %) and rural women (13·3 %) bearing the largest burden of DBM.

**Fig. 3:**
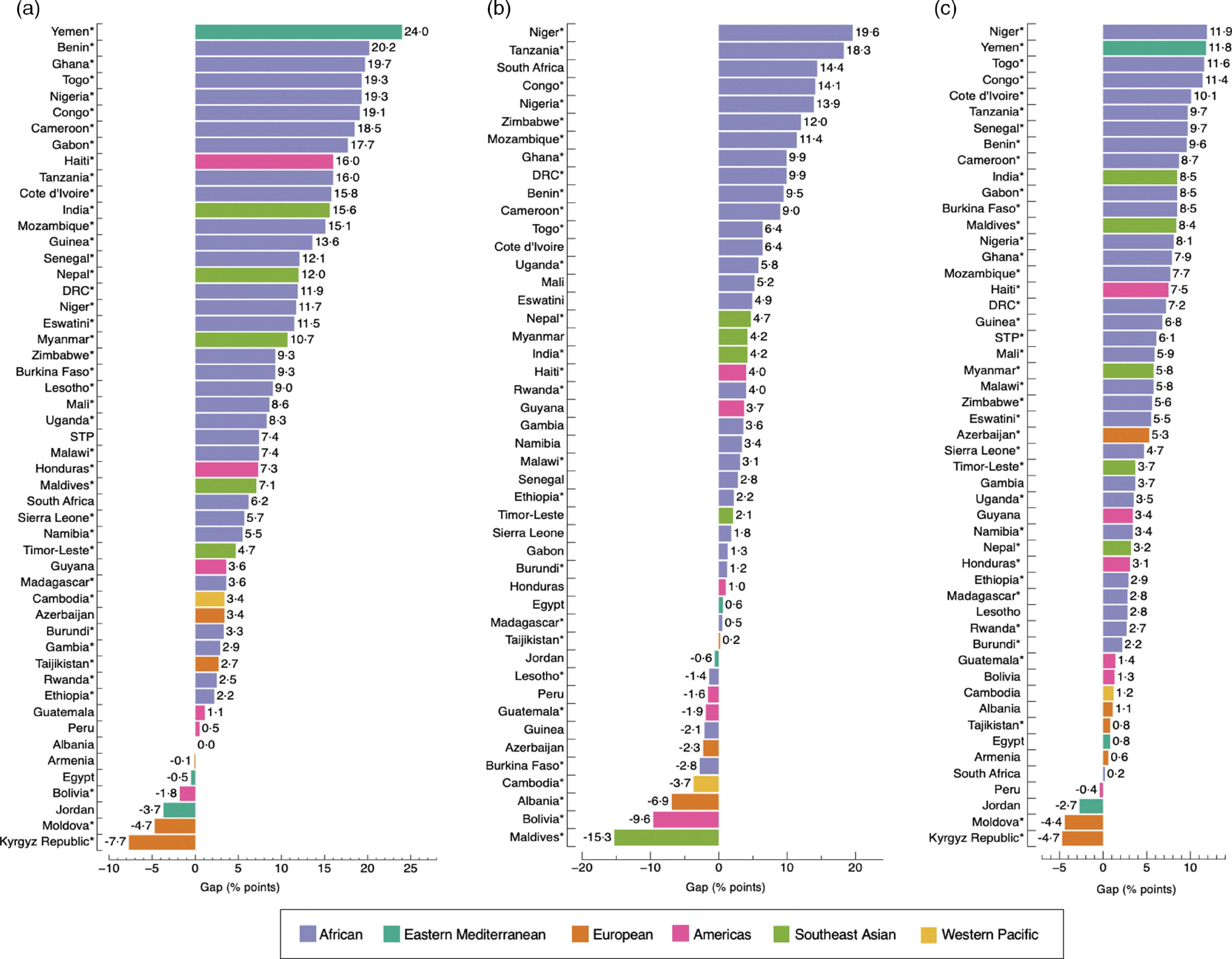
Absolute gap difference of concurrent overweight/obesity and anaemia by wealth quintile (a); education level (b); and area of residence (c) among adult women. Positive values mean that concurrent overweight/obesity and anaemia are more prevalent in the richest quintile (Q5), highest education level (E4) and in urban areas when compared to the poorest quintile (Q1), lowest education level (E1) and rural areas. Negative values mean the opposite. (*) *P*-value <0·05. In Fig. b, Yemen was not included because data on education level was missing. Likewise, countries with a sample size <25 observations for E1 or E4 were excluded. DRC: Democratic of the Congo; STP: Sao Tome and Principe

Figure 3 shows the absolute inequality of the prevalence of overweight/obesity and anaemia among adult women by the three socio-economic measures. Countries showed more or less inequalities regardless of the magnitude of DBM (see online Supplemental Tables 4–7). The largest gaps were observed in Yemen, with a 24·0 percentage-point difference (*P* < 0·001) in DBM prevalence by household wealth; and in Niger, with a 19·6 percentage-point difference (*P* < 0·001) by education level and 11·9 percentage-point difference (*P* < 0·001) by area of residence. Gaps were positive in 86·3 % (44/51), 76·1 % (35/46) and 92·2 % (47/51) of countries by household wealth, education level and area of residence, respectively, indicating higher prevalence of concurrent overweight/obesity and anaemia among the richest quintile than the poorest, the most educated than the least educated and urban than rural residents (Fig. 3). The opposite (i.e. higher prevalence among the poorest than the richest, the more educated than the least educated and rural than urban residents), was only observed in 11·8 % (6/51) of countries by household wealth, 23·9 % (11/46) of countries by education level and 7·8 % (4/51) of countries by area of residence. In one country (Albania), the inequality gap in the prevalence of DBM was 0·0 percentage-points among the richest and the poorest group (*P* = 0·278) (Fig. 3(a)). Differences observed across groups were significant in 80·4 % (41/51) of countries by household wealth and 78·4 % (40/51) by area of residence (Fig. 3(a) and (c)), and in 60·9 % (28/46) of countries by education level (Fig. 3(b)).

### Overweight/obesity and anaemia among adolescent girls

The pooled prevalence of concurrent overweight/obesity and anaemia among adolescent girls was 4·5 % (95 % CI 4·0, 5·0; *I* ^2^ 96·2 %), ranging from 0·5 % in Madagascar and Timor-Leste to 21·5 % in Jordan (Fig. 2 and see online Supplemental Table S4). The pooled regional prevalence ranged from 2·5 % (95 % CI 1·5, 3·4) in the European region to 11·8 % (95 % CI 4·2, 19·3) in the Eastern Mediterranean region.

Patterns in the distribution of concurrent overweight/obesity and anaemia were similar to those of adult women, although with more variation across and within regions. Overall, the highest prevalence was found in the fifth richest quintile (5·7 %), third education level (4·3 %), and urban residents (5·4 %) (see online Supplemental Tables 8–10). A distinct pattern was also observed in the European region, where the prevalence of DBM was higher in rural residents (see online Supplemental Table 10), and in the Americas region, with a higher prevalence of DBM observed among the least educated (see online Supplemental Table 9).

**Fig. 4:**
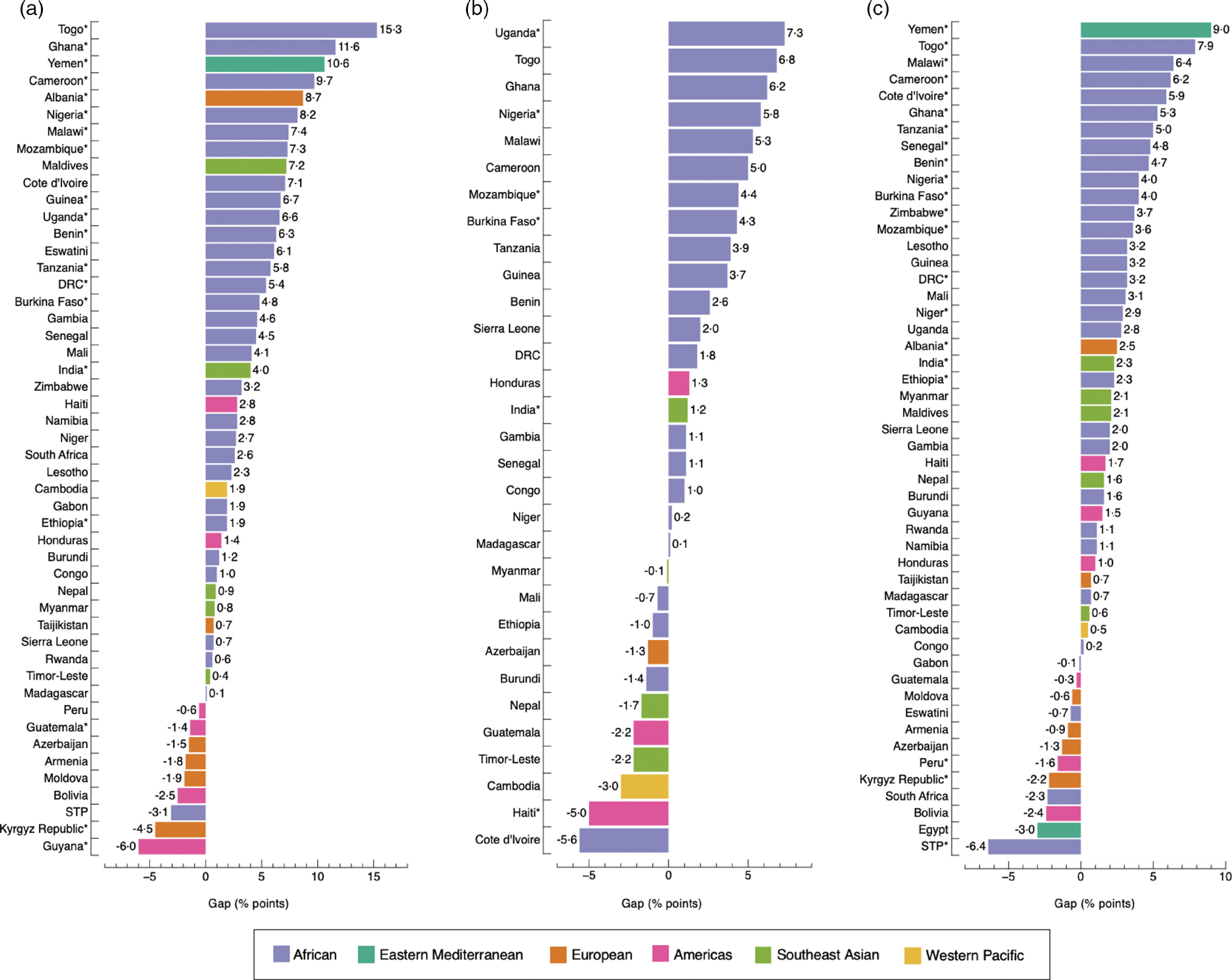
Absolute gap difference of concurrent overweight/obesity and anaemia by wealth quintile (a); education level (b); and area of residence (c) among adolescent girls. Positive values mean that concurrent overweight/obesity and anaemia are more prevalent in the richest quintile (Q5), highest education level (E3) and in urban areas when compared with the poorest quintile (Q1), lowest education level (E1) and rural areas. Negative values mean the opposite. (*) *P*-value <0·05. Countries with a sample size <25 observations were excluded. In Fig. b, Yemen was not included because data on education level was missing. DRC: Democratic of the Congo; STP: Sao Tome and Principe

The largest gaps were observed in Togo, with a 15·3 percentage-point difference (*P* < 0·001) in DBM prevalence by household wealth; in Uganda, with a 19·5 percentage-point difference (*P* < 0·001) by education level; and in Yemen, with a 9·0 percentage-point difference (*P* < 0·001) by area of residence (Fig. 4 and see online Supplemental Tables S8–S10). Gaps were positive in 81·6 % (40/49), 64·5 % (20/31) and 76·0 % (38/50) of countries by household wealth, education level and area of residence, respectively, whereas these were negative in 18·4 % (9/49) of countries by household wealth, 35·5 % (11/31) by education level and 24·0 % (12/50) by area of residence (Fig. 4). Differences observed across groups were significant in less than half of the countries for the three socio-economic measures. For example, by education level, differences observed between the least and most educated were only significant in Uganda, Nigeria, Mozambique, Burkina Faso, India and Haiti.

### Overweight/obesity and anaemia among children

The pooled prevalence of concurrent overweight/obesity and anaemia among children was 3·0 % (95 % CI 2·7, 3·3; *I* ^2^ 97·1 %), ranging from 0·4 % in Nepal to 9·1 % in South Africa (Fig. 2 and Supplemental Table S4). The pooled regional prevalence ranged from 1·2 % (95 % CI 0·8, 1·7) in the Southeast Asian region to 3·8 % (95 % CI 2·4, 5·2) in the European region.

Overall, the prevalence of concurrent overweight/obesity and anaemia among children was the lowest across the three age groups studied (see online Supplemental Table 4); however, Fig. 2 shows that the prevalence of DBM among children was higher than that of adolescent girls in fourteen countries and in the European region.

Patterns in the distribution of concurrent overweight/obesity and anaemia differed from those among adult women and adolescent girls (see online Supplemental Tables 11–13). Overall, the highest prevalence was in the second and fifth wealth quintile (2·8 %), second maternal education level (2·7 %) and in rural areas (3·0 %). Nevertheless, in the African region the distribution of the DBM by the three socio-economic measures among children emulated that of adult women and adolescent girls, with the highest prevalence in the fifth quintile, fourth education level and urban residents. For all WHO regions, the prevalence of overweight/obesity and anaemia was slightly higher among boys than girls (3·2 % *v*. 2·5 %) (see online Supplemental Table 14).

**Fig. 5:**
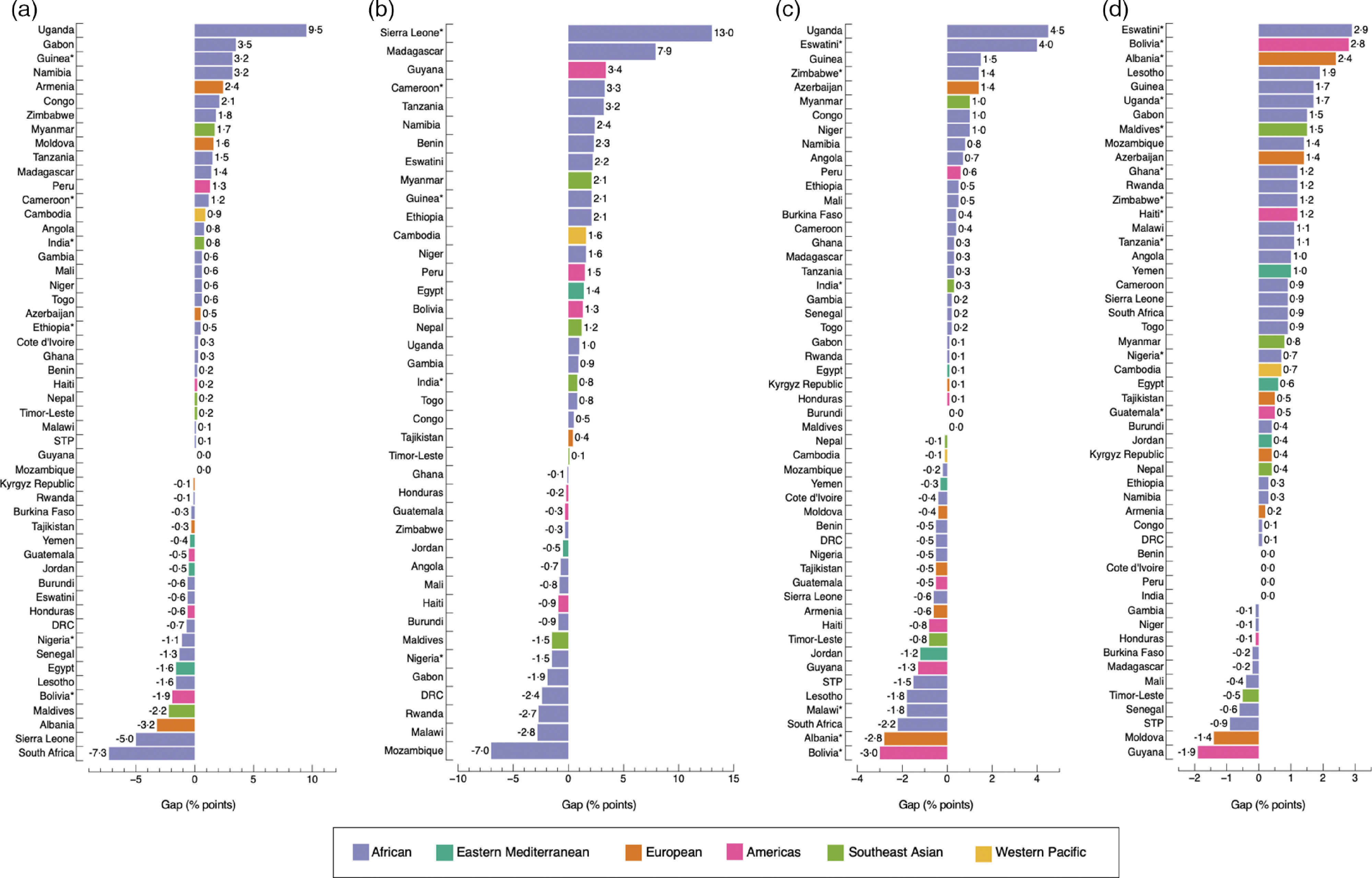
Absolute gap difference of concurrent overweight/obesity and anaemia by wealth quintile (a); maternal education level (b); area of residence (c); and sex (d) among children. Positive values mean that concurrent overweight/obesity and anaemia are more prevalent in the richest quintile (Q5), highest education level (E4), urban areas and among boys, when compared with the poorest quintile (Q1), lowest education level (E1), rural areas and girls. Negative values mean the opposite. (*) *P*-value <0·05. In b, Yemen was not included because data on education level were missing. Likewise, countries with a sample size <25 observations for E1 or E4 were excluded. DRC: Democratic of the Congo; STP: Sao Tome and Principe

The widths of inequality gaps were smaller than those of adult women and adolescent girls overall, with only fifteen instances where gaps were greater than 3·0 percentage-points (Fig. 5(a), (b), (c) and (d)). The largest gaps were observed in Uganda, with a 9·5 (*P* = 0·891) and a 4·5 percentage-point difference (*P* = 0·857) in DBM prevalence by household wealth and area of residence, respectively; and in Sierra Leone, with a 15·9 percentage-point difference (*P* = 0·028) by maternal education level (Fig. 5). The prevalence of DBM was the same, with a gap of 0·0 percentage-points, among the richest and poorest groups in Mozambique and Guyana; urban and rural residents in Maldives and Burundi and boys and girls in India, Peru, Cote d’Ivoire and Benin. Differences observed among groups were significant in 11·5 % (6/52), 11·1 % (5/45), 11·5 % (6/52) and 21·2 % (11/52) of countries by household wealth, maternal education level, area of residence and sex, respectively.

## Discussion

The present study provides evidence of the magnitude and distribution of overweight/obesity and anaemia at the individual level among adult women, adolescent girls and children, using nationally representative DHS samples from fifty-two LMIC. We show that concurrent overweight/obesity and anaemia were common among adult women, with more than 1 in 10 simultaneously affected by the two forms of malnutrition; however, it was low among adolescent girls and children. The overall pooled prevalence estimate for adult women (12·4 %) was almost three times that of the prevalence estimate for adolescent girls (4·5 %) and was four times higher than that of children (3·0 %). Important variations exist in the prevalence of concurrent overweight/obesity and anaemia across LMIC and WHO regions.

Williams and colleagues^(28)^ recently reported that the prevalence of overweight/obesity and anaemia at the individual level among women of reproductive age (15–49 years old) ranged between 1·0 % and 18·6 % (median = 8·6 %); however, these data were estimated in sixteen countries, including LMIC and high-income countries, using surveys from the BRINDA project (https://brinda-nutrition.org/). The prevalence estimates that we present for adult women and adolescent girls are not directly comparable with those from the above-mentioned study^(28)^, as we calculated separate estimates for adult women (20–49 years old) and adolescent girls (15–49 years old), and only included LMIC (*n* 51). By providing separate estimates, we show that the prevalence of concurrent overweight/obesity and anaemia among adolescent girls was similar to that of younger children (6–59 months), and that the prevalence of DBM among adult women was as high as 30·0 % in countries such as Maldives, Jordan or Gabon. Another study analysing anthropometric data to quantify the magnitude of DBM at the individual level (i.e. concurrent overweight/obesity and stunting) also found a low burden among adolescent girls (12–15 years old) living in LMIC, with prevalence estimates ranging from 0·0 % to 7·7 %^(39)^. We additionally observed that the difference in estimates among women and girls (7·9 percentage-points) was primarily driven by a higher prevalence of overweight/obesity among the adult population (37·5 % *v*. 11·3 %), with both groups bearing a similar burden of anaemia (38·7 % *v*. 38·8 %).

Our estimates among children under-five are slightly higher than those previously reported^(29)^. The earlier study calculated the prevalence of concurrent overweight/obesity and anaemia, ranging from 0·0 % to 5·0 % (median= 1·4 %)^(29)^. We found, however, the highest prevalences of DBM among children ranging from 6·0 % to 9·1 % in Gabon, Mozambique, Bolivia, Eswatini, Sierra Leone, Sao Tome and Principe and South Africa; none of which were included in Engle-Stone and colleagues’ study^(29)^. We present overlapping estimates for five countries, with a difference between 2·8 percentage-points in Cameroon (4·5 % *v*. 1·7 %) and 0·7 percentage-points in Malawi (2·6 % *v*. 3·3 %). Only in Cote d’Ivoire, our estimate is lower (2·9 %) than the previously reported value (3·7 %)^(29)^, although data analysed was collected 3 years apart, and thus, changes in the burden of overweight/obesity and anaemia would be expected. Nevertheless, we use data from the same year for Cambodia (2014) and Malawi (2015–2016) and obtained a higher prevalence of concurrent overweight/obesity and anaemia for both countries. Although the difference is low (<1·5 percentage-points), presenting different estimates can cause confusion for governments, or policy and programme planners, and hinder appropriate monitoring of the DBM. Significant differences in Hb distributions across different surveys have been discussed previously and attributed to factors such as humidity, the HemoCue® model used for data collection, or the use of different survey sampling procedures^(40)^. This may have had an influence in the prevalence of DBM; however, it is worth noting, that for Cambodia, our sample size is significantly higher (3799 *v*. 406), which may explain the difference in estimates from a 0·0 %^(29)^ to a 1·4 % in our study.

Overall, the co-occurrence of overweight/obesity and anaemia followed an inverse social gradient, emulating the distribution of overweight/obesity in LMIC^(2,41–45)^. The prevalence of DBM was higher among the richest quintile and most educated groups, and urban residents had consistently a larger burden than their rural counterparts in most countries, especially among adult women and adolescent girls. This is in alignment with previous studies reporting a higher prevalence of overweight/obesity and anaemia at the individual level among the most socio-economically advantaged and in urban areas^(28,46)^. Among children, we observed little inequalities by household wealth, maternal education level, area of residence and sex; however, the prevalence of DBM was slightly higher in rural areas, with the exception of the African region, and in boys.

Larger inequality gaps were observed among adult women and adolescent girls, when compared to children, for which gaps were overall small (< 3·0 percentage-points). Across the three age groups, the largest gaps by the three socio-economic measures were found in African countries (e.g. Niger, Uganda, Togo and Sierra Leone) and in Yemen. There were variations in the level of inequality across countries with a similar burden of DBM. For example, among adult women, the difference between the richest and the poorest group was 7·1 percentage-points in Maldives, where the prevalence of concurrent overweight/obesity was 33·6 %, whereas in Gabon, with a 30·1 % prevalence, the inequality gap was 17·7 percentage-points. This points to the need for context-specific solutions that appropriately address the nutritional needs of a country’s population. In countries with a high prevalence of concurrent overweight/obesity and anaemia across all socio-economic groups, population-based interventions that target both forms of malnutrition might be more suitable than targeting only the richest quintiles.

Differences observed by household wealth, education level and area of residence were significant for most countries among adult women; however, these were significant in <50 % of countries among adolescent girls, and only a handful of countries among children. Other studies had previously identified no significant associations between socio-demographic measures (i.e. sex, area of residence, household wealth and education) and concurrent overweight/obesity and anaemia among children under-five^(29)^ and mixed results among women of reproductive age^(28)^.

Our study has several limitations that need to be considered. First, we used anaemia as a proxy for micronutrient deficiencies in the absence of individual micronutrient data (e.g. Fe, vitamin A, etc.) in DHS surveys. The complex aetiology of anaemia in LMIC has been described in detail somewhere else^(47)^. In countries with a low infection burden, the proportion of anaemic women of reproductive age and children under-five with Fe deficiency is believed to be 71·0 % and 50·0 %, respectively, whereas in countries with a high infection burden, the proportion was estimated to be 58·0 % among children and as low as 35·1 % among women^(48,49)^. Second, we did not measure the independence of overweight/obesity and anaemia. Excess weight has been previously linked with Fe deficiency^(50–52)^, but this association would not necessarily be true for anaemia^(53)^. Recent evidence found that overweight/obesity and anaemia were either two independent conditions in LMIC or were negatively associated (i.e. odds of anaemia were higher among normal weight than overweight/obese women)^(28,29)^. Third, some categories created for the stratified analysis resulted in small sample sizes, and therefore, we could not include certain countries into the regional prevalence, and in some cases, the regional estimate could not be calculated. Fourth, for some WHO regions, the number of countries with data on both, anthropometric measures and anaemia was low, and thus, they might be underrepresented. For example, the Western Pacific region, only had one country with available data (i.e. Cambodia), and the Eastern and Mediterranean region had three countries (i.e. Egypt, Jordan and Yemen). Although most DHS surveys include weight and height measurements, not all collect data on Hb levels, and thus, a number of potential countries could not be included in our study. Fifth, it is important to note that we did not use complex measures of inequalities (i.e. slope index of inequality) which would have allowed to take into account differences across all socio-economic groups, including the intermediate groups; however, in this study, we were only interested in measuring differences between the least and most disadvantageous groups (e.g. Q5 *v*. Q1 and E4 *v*. E1). Sixth, although we sought to include the most recent DHS surveys available for each country, these were spread out over several years (from 2003 in Madagascar to 2018 in Nigeria). This might have influenced the prevalence estimates obtained and might not reflect the current magnitude of concurrent overweight/obesity and anaemia, particularly for those countries with data from older surveys.

Despite these limitations, our study included a large number of LMIC (*n* 52), and we were able to analyse overall large sample sizes from nationally representative surveys. Additionally, we were able to present the magnitude of concurrent overweight/obesity and anaemia for adult women and adolescent girls separately and stratified by different socio-economic measures, which could aid in informing more precise policy responses within individual countries.

The mechanisms underpinning the simultaneous presence of overweight/obesity and anaemia across the three age groups in LMIC are likely to be multifactorial due to the varied and complex aetiology of anaemia^(47–49)^. Nevertheless, the fact that we found the highest prevalence of concurrent overweight/obesity and anaemia among adult women living in urban areas and those from the richest quintiles (where the risk of infectious diseases is likely to be lower) could point to unhealthy dietary practices and/or overweight/obesity as plausible factors contributing to the DBM for this age group. Nevertheless, food insecurity could also lead to a higher consumption of energy-dense nutrient-poor foods and explain why some LMIC had a high prevalence of DBM across all wealth quintiles^(54)^. First, rapid changes in the food systems of LMIC are resulting in an increased availability of ultra-processed foods, which are easily accessible and affordable^(2,5,55,56)^. A high consumption of these foods rich in fats and poor in vitamins and minerals could make women more prone to develop both weight gain and anaemia as a result of Fe deficiency (or other micronutrient deficiencies) from the diet. Second, overweight/obesity can also lead to Fe deficiency due to increases in hepcidin, which reduces the absorption of Fe from the diet^(52)^. Data on dietary practices, as well as on the causes of anaemia, are needed in nationally representative surveys (e.g. DHS), in order to better elucidate the causes of concurrent overweight/obesity and anaemia.

Double-duty actions, proposed to address forms of undernutrition and overweight/obesity^(15)^, might have a positive effect in preventing and reducing the dual burden of overweight/obesity and anaemia (e.g. changes in the food environment conducive to supporting healthy diets, scale up of programmes that protect breast-feeding and offer a better guidance for complementary feeding practices, counselling about healthy eating during antenatal care, etc). Yet, the effectiveness of double-duty actions still needs to be tested in this context. For women who are already living with overweight/obesity, weight reduction could translate into better absorption of micronutrients (e.g. Fe) from the diet and hence increased Hb levels.

Furthermore, further research is needed to elucidate the full implications of concurrent overweight/obesity and anaemia, particularly among women of reproductive age, for whom the burden was highest. Women who enter pregnancy with concurrent overweight/obesity and anaemia might be confronted with adverse maternal, obstetric and birth outcomes related to both, anaemia and adiposity. Management of both of these conditions would be particularly challenging for many health systems in LMIC which are underdeveloped^(57)^ and might need to be redesigned to contend with women presenting with the dual burden of overweight/obesity and anaemia. Likewise, adolescent girls, who showed an overall low prevalence of DBM, could be a crucial second window of opportunity to act early on and prevent the detrimental intergenerational consequences of malnutrition, as well as to improve pregnancy outcomes^(6)^.

In conclusion, our study demonstrated a high prevalence of concurrent overweight/obesity and anaemia among adult women and a much lower prevalence among adolescent girls and children under-five. Concurrent overweight/obesity and anaemia were unequally distributed across wealth quintiles, education levels and area of residence. As the prevalence of overweight/obesity continues to increase rapidly across LMIC in response to the nutrition transition^(2,5)^, this may translate in increases in concurrent overweight/obesity and anaemia. Similarly, changes in the distribution of this form of DBM might also occur, in view of the obesity shift towards rural residents and the poor^(42,57–59)^. Given the variation in the magnitude and distribution observed, context-specific, multifaceted and equity-focused programmatic and policy responses that address all forms of malnutrition are needed.

## Supplementary material

For supplementary material accompanying this paper visit https://doi.org/10.1017/S1368980021002512. click here to view supplementary material
