# Supplementary material for: The co-occurrence of overweight/obesity and anaemia among adult women, adolescent girls and children living in fifty-two low- and middle-income countries
Source: Public Health Nutr. 2021 Jun 9;25(6):1595–606. doi: 10.1017/S1368980021002512 (PMC9991775; doi:10.1017/S1368980021002512)
Supplement: Supplementary file 1 [file S1368980021002512sup001.docx]

**Supplemental Figure 1.** Flowchart of study participants included in the analysis.

**
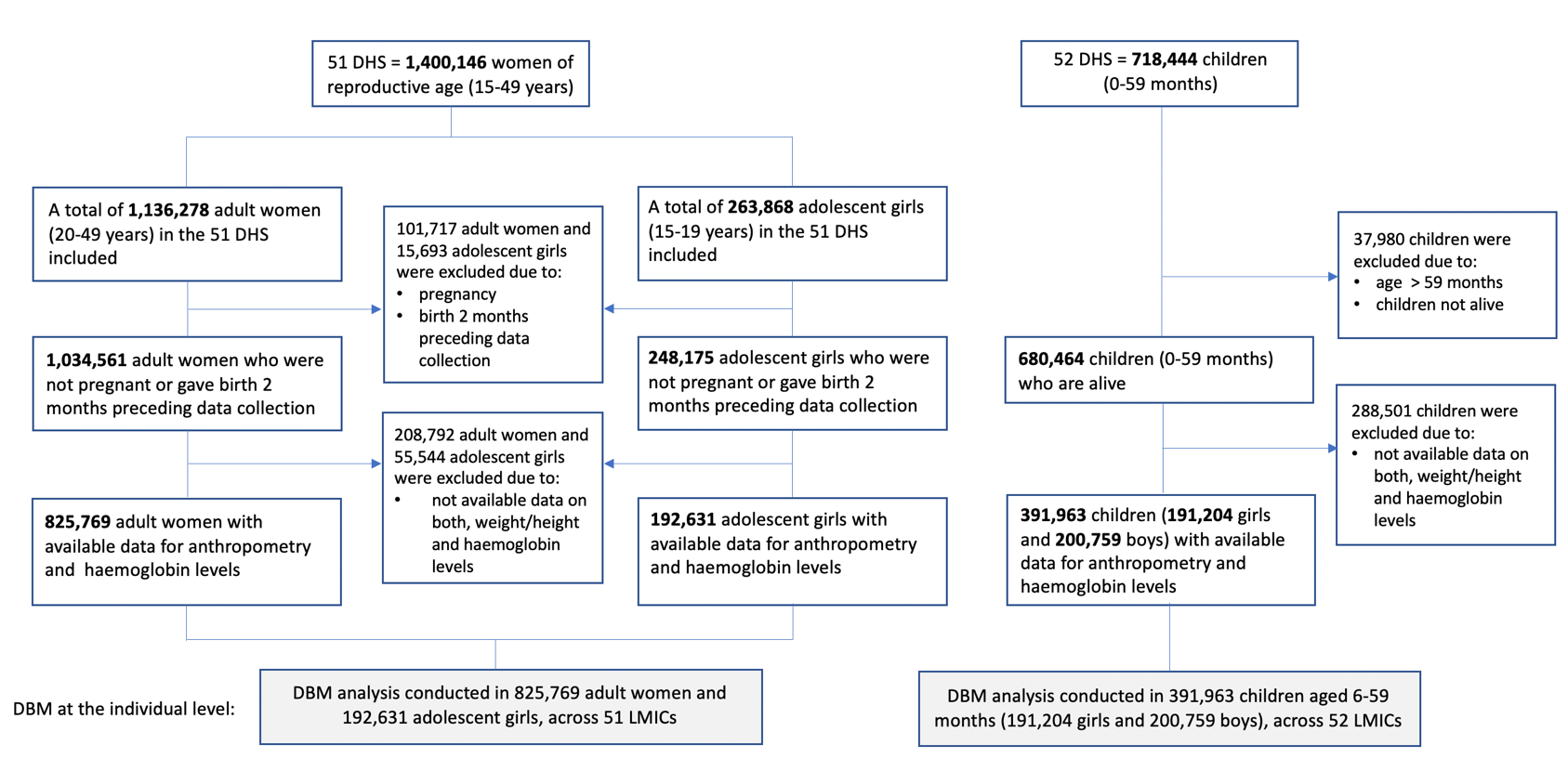
**

**Supplemental Table 1.** Characteristics of adult women (20-49 years old) included in the study.

|  |  | |  |  |  |
| --- | --- | --- | --- | --- | --- |
|  | **Adult women (20-49 years old)** | | | | |
| **Country and survey year** | **Sample size,** *n* | **Age, years** | **Height, cm** | **Weight, kg** | **Hb, g/dL** |
| **African region** |  |  |  |  |  |
| Benin 2017-18 | 5,274 | 32.0 [25.0, 39.0] | 158.7 [155.0, 162.7] | 57.2 [50.5, 66.8] | 11.8 [10.7, 12.7] |
| Burkina Faso 2010 | 5,671 | 31.0 [25.0, 39.0] | 162.0 [158.0, 165.7] | 55.0 [50.0,61.3] | 12.1 [10.9, 13.1] |
| Burundi 2016-17 | 5,675 | 30.0 [25.0, 38.0] | 155.7 [151.6, 159.8] | 50.0 [45.5, 55.5] | 12.5 [11.4, 13.4] |
| Cameroon 2011 | 5,148 | 30.0 [24.0, 39.0] | 160.3 [156.3, 164.6] | 60.3 [53.2, 70.1] | 12.4 [11.3, 13.4] |
| Congo 2011-12 | 3,779 | 32.0 [26.0, 39.0] | 158.7 [154.5, 162.8] | 54.7 [48.7, 63.4] | 11.9 [10.9, 12.7] |
| Cote d'Ivoire 2011-12 | 3,115 | 31.0 [25.0, 39.0] | 159.0 [154.9, 163.2] | 57.0 [51.2, 64.9] | 11.9 [10.8, 12.9] |
| DRC 2013-14 | 5,930 | 31.0 [25.0, 39.0] | 157.1 [152.3, 161.8] | 52.8 [47.3, 59.6] | 12.4 [11.3, 13.3] |
| Eswatini 2006-07 | 3,102 | 31.0 [24.0, 39.0] | 158.9 [154.8, 163.1] | 66.8 [57.8, 78.3] | 12.8 [11.7, 13.8] |
| Ethiopia 2016 | 9,878 | 30.0 [25.0, 38.0] | 157.4 [153.3, 161.6] | 50.6 [45.7, 57.3] | 13.0 [11.9, 14.0] |
| Gabon 2012 | 3,631 | 33.0 [25.0, 41.0] | 158.9 [154.7, 163.2] | 61.3 [53.1, 72.3] | 11.7 [10.5, 12.6] |
| Gambia 2013 | 2,923 | 30.0 [24.0, 37.0] | 162.2 [157.9, 166.5] | 58.1 [51.5, 67.4] | 11.5 [10.2, 12.5] |
| Ghana 2014 | 3,363 | 32.0 [25.0, 40.0] | 159.4 [155.5, 163.4] | 61.2 [53.4, 71.1] | 12.3 [11.3, 13.2] |
| Guinea 2018 | 3,439 | 32.0 [25.0, 40.0] | 159.2 [155.2, 163.6] | 58.1 [51.7, 66.5] | 12.2 [11.1, 13.1] |
| Lesotho 2014 | 2,383 | 31.0 [25.0, 39.0] | 157.5 [153.8, 161.6] | 63.2 [54.7, 74.6] | 13.0 [11.9, 14.1] |
| Madagascar 2008-09 | 5,585 | 32.0 [26.0, 40.0] | 154.0 [150.0, 158.0] | 48.0 [43.9, 53.0] | 12.4 [11.5, 13.4] |
| Malawi 2015-16 | 5,553 | 30.0 [25.0, 38.0] | 156.2 [152.5, 159.9] | 54.6 [49.5, 61.7] | 12.7 [11.6, 13.6] |
| Mali 2018 | 3,375 | 31.0 [25.0, 38.0] | 162.1 [157.8, 166.2] | 60.7 [53.5, 71.0] | 11.5 [10.4, 12.5] |
| Mozambique 2011 | 9,016 | 31.0 [25.0, 39.0] | 156.3 [152.2, 160.4] | 54.3 [49.0, 61.9] | 11.9 [10.7, 12.9] |
| Namibia 2013 | 3,913 | 36.0 [26.0, 47.0] | 161.5 [156.5, 166.0] | 62.2 [52.6, 75.0] | 13.2 [12.2, 14.1] |
| Niger 2012 | 3,303 | 30.0 [25.0, 38.0] | 160.5 [157.0, 164.5] | 57.0 [50.5, 64.9] | 12.3 [11.2, 13.2] |
| Nigeria 2018 | 10,029 | 32.0 [26.0, 40.0] | 158.5 [154.5, 162.4] | 57.1 [50.3, 66.8] | 11.7 [10.8, 12.7] |
| Rwanda 2014-15 | 4,685 | 31.0 [25.0, 38.0] | 157.0 [153.0, 161.3] | 55.5 [50.3, 62.2] | 13.3 [12.3, 14.1] |
| STP 2008-09 | 1,620 | 32.0 [26.0, 40.0] | 159.8 [156.0, 164.0] | 60.0 [54.0, 69.0] | 12.3 [11.4, 13.2] |
| Senegal 2010-11 | 3,614 | 30.0 [24.0, 38.0] | 163.2 [158.7, 167.4] | 57.0 [50.5, 65.7] | 11.8 [10.7, 12.7] |
| Sierra Leone 2013 | 5,271 | 32.0 [25.0, 39.0] | 158.3 [154.2, 162.4] | 55.9 [50.4, 62.7] | 12.2 [11.1, 13.1] |
| South Africa 2016 | 2,284 | 33.0 [26.0, 41.0] | 158.7 [154.2, 162.5] | 70.5 [59.7, 83.3] | 12.8 [11.6, 13.9] |
| Tanzania 2015-16 | 8,871 | 32.0 [25.0, 40.0] | 156.5 [152.5, 160.5] | 56.3 [49.8, 65.5] | 12.2 [11.1, 13.2] |
| Togo 2013-14 | 3,390 | 32.0 [25.0, 40.0] | 159.1 [155.2, 163.0] | 58.0 [51.6, 67.3] | 12.2 [11.2, 13.2] |
| Uganda 2016 | 3,868 | 30.0 [25.0, 39.0] | 159.2 [155.1, 163.4] | 56.7 [50.8, 64.2] | 12.7 [11.7, 13.7] |
| Zimbabwe 2015 | 6,515 | 31.0 [25.0, 38.0] | 160.5 [156.5, 164.8] | 62.3 [54.9, 73.2] | 12.9 [11.8, 13.9] |
| **Eastern Mediterranean region** | | | | | |
| Egypt 2014 | 6,077 | 34.0 [28.0, 41.0] | 159.2 [155.9, 162.7] | 75.1 [66.6, 85.2] | 12.6 [12.0, 13.2] |
| Jordan 2017-18 | 5,879 | 36.0 [29.0, 43.0] | 159.6 [155.8, 163.4] | 70.2 [62.0, 80.0] | 12.1 [11.1, 13.0] |
| Yemen 2013 | 4,730 | 30.0 [24.0, 38.0] | 153.9 [150.1, 157.9] | 51.8 [44.9, 61.5] | 10.8 [9.5, 12.0] |
| **European region** |  |  |  |  |  |
| Albania 2017-18 | 12,360 | 43.0 [32.0, 52.0] | 159.1 [154.9, 163.7] | 67.4 [59.3, 77.0] | 12.8 [12.1, 13.5] |
| Armenia 2015-16 | 4,868 | 33.0 [27.0, 41.0] | 160.3 [156.4, 163.7] | 64.5 [56.7, 74.3] | 13.4 [12.5, 14.1] |
| Azerbaijan 2006 | 6,304 | 35.0 [27.0, 42.0] | 158.7 [154.8, 162.3] | 63.7 [55.8, 72.7] | 12.4 [11.4, 13.2] |
| Kyrgyz Republic 2012 | 5,765 | 33.0 [26.0, 41.0] | 159.7 [155.6, 163.4] | 62.3 [54.7, 71.3] | 12.6 [11.5, 13.4] |
| Moldova 2005 | 5,495 | 35.0 [27.0, 43.0] | 161.2 [157.4, 165.1] | 65.0 [57.0, 76.0] | 12.7 [11.8, 13.5] |
| Tajikistan 2017 | 7,820 | 32.0 [26.0, 40.0] | 158.1 [154.4, 161.8] | 60.2 [52.5, 70.2] | 12.2 [11.2, 13.0] |
| **Americas region** |  |  |  |  |  |
| Bolivia 2008 | 4,063 | 32.0 [26.0, 40.0] | 151.8 [148.1, 156.0] | 59.2 [52.8, 67.5] | 12.5 [11.4, 13.4] |
| Guatemala 2014-15 | 18,180 | 32.0 [25.0, 39.0] | 149.1 [144.9, 153.5] | 58.5 [50.6, 67.6] | 13.4 [12.5, 14.2] |
| Guyana 2009 | 3,402 | 34.0 [27.0, 41.0] | 156.0 [151.1, 161.1] | 62.9 [53.8, 74.2] | 12.5 [11.4, 13.3] |
| Haiti 2016-17 | 6,587 | 31.0 [25.0, 39.0] | 159.5 [155.4, 163.6] | 59.0 [51.8, 69.2] | 12.1 [11.0, 13.1] |
| Honduras 2011-12 | 15,323 | 32.0 [25.0, 39.0] | 153.0 [148.7, 157.2] | 60.9 [52.5, 71.1] | 13.3 [12.4, 14.1] |
| Peru 2012 | 17,898 | 34.0 [27.0, 41.0] | 151.8 [148.1, 155.6] | 60.5 [53.7, 68.4] | 13.1 [12.3, 13.9] |
| **Southeast Asian region** |  |  |  |  |  |
| India 2015-16 | 524,796 | 32.0 [26.0, 40.0] | 152.1 [148.4, 156.0] | 50.0 [43.8, 57.5] | 11.9 [10.8, 12.8] |
| Maldives 2016-17 | 5,570 | 33.0 [27.0, 40.0] | 152.2 [148.6, 156.0] | 59.6 [52.2, 68.5] | 11.7 [10.7, 12.5] |
| Myanmar 2015-16 | 10,054 | 34.0 [27.0, 42.0] | 152.7 [149.2, 156.2] | 51.8 [45.7, 59.6] | 12.1 [11.2, 13.1] |
| Nepal 2016 | 4,787 | 32.0 [25.0, 40.0] | 151.6 [148.0, 155.4] | 50.1 [44.6, 57.5] | 12.3 [11.4, 13.2] |
| Timor-Leste 2016 | 2,870 | 32.0 [25.0, 41.0] | 152.0 [148.2, 155.5] | 47.6 [42.8, 53.1] | 12.9 [12.1, 13.6] |
| **Western Pacific region** |  |  |  |  |  |
| Cambodia 2014 | 8,703 | 32.0 [26.0, 41.0] | 153.2 [149.7, 156.7] | 51.4 [46.1, 58.2] | 12.2 [11.3, 13.0] |

Values are medians and interquartile ranges, unless otherwise indicated.

Hb, Haemoglobin levels; DRC, Democratic Republic of the Congo; STP, Sao Tome and Principe.

**Supplemental Table 2.** Characteristics of adolescent girls (15-19 years old) included in the study.

|  |  | |  |  |  |
| --- | --- | --- | --- | --- | --- |
|  | **Adolescent girls (15-19 years old)** | | | | |
| **Country and survey year** | **Sample size,** *n* | **Age, years** | **Height, cm** | **Weight, kg** | **Hb, g/dL** |
| **African region** |  |  |  |  |  |
| Benin 2017-18 | 1,481 | 17.0 [15.0, 18.0] | 157.0 [153.1, 161.1] | 50.2 [45.6, 55.2] | 11.7 [10.7, 12.6] |
| Burkina Faso 2010 | 1,512 | 17.0 [16.0, 18.0] | 159.2 [155.1, 163.0] | 51.3 [46.8, 56.7] | 12.1 [10.9, 13.0] |
| Burundi 2016-17 | 1,891 | 17.0 [16.0, 18.0] | 154.1 [149.9, 158.4] | 48.4 [43.6, 53.2] | 12.5 [11.6, 13.5] |
| Cameroon 2011 | 1,654 | 17.0 [16.0, 18.0] | 158.7 [154.8, 163.0] | 54.8 [49.7, 60.8] | 12.4 [11.4, 13.3] |
| Congo 2011-12 | 959 | 17.0 [15.0, 18.0] | 157.2 [153.1, 161.4] | 49.8 [45.4, 54.7] | 11.9 [11.0, 12.7] |
| Cote d'Ivoire 2011-12 | 843 | 17.0 [16.0, 18.0] | 157.0 [153.5, 161.6] | 52.2 [46.8, 57.3] | 11.9 [10.9, 12.9] |
| DRC 2013-14 | 1,706 | 17.0 [16.0, 18.0] | 155.3 [150.0, 159.5] | 49.5 [44.5, 54.6] | 12.3 [11.3, 13.3] |
| Eswatini 2006-07 | 1,112 | 17.0 [16.0, 18.0] | 158.1 [153.5, 162.2] | 56.7 [51.0, 62.5] | 12.9 [11.9, 13.7] |
| Ethiopia 2016 | 3,006 | 17.0 [16.0, 18.0] | 156.9 [152.8, 161.0] | 48.4 [43.6, 53.6] | 13.1 [12.1, 14.1] |
| Gabon 2012 | 1,036 | 17.0 [16.0, 18.0] | 157.5 [153.3, 161.5] | 52.1 [47.1, 58.1] | 11.6 [10.5, 12.5] |
| Gambia 2013 | 958 | 17.0 [16.0, 18.0] | 160.7 [157.0, 165.0] | 51.5 [47.0, 57.7] | 11.6 [10.7, 12.6] |
| Ghana 2014 | 833 | 17.0 [16.0, 18.0] | 158.4 [154.9, 162.3] | 52.8 [48.0, 58.0] | 12.0 [11.0, 12.8] |
| Guinea 2018 | 1,097 | 17.0 [15.0, 18.0] | 157.2 [152.6, 161.8] | 51.9 [47.5, 57.8] | 12.1 [11.0, 12.9] |
| Lesotho 2014 | 731 | 17.0 [16.0, 18.0] | 156.0 [152.2, 159.6] | 52.8 [48.2, 58.9] | 13.1 [12.1, 14.0] |
| Madagascar 2008-09 | 1,659 | 17.0 [16.0, 18.0] | 151.5 [147.9, 155.6] | 45.0 [41.0, 49.0] | 12.4 [11.6, 13.4] |
| Malawi 2015-16 | 1,507 | 17.0 [16.0, 18.0] | 155.4 [151.5, 159.2] | 50.9 [46.5, 56.1] | 12.5 [11.6, 13.4] |
| Mali 2018 | 863 | 17.0 [16.0, 18.0] | 160.5 [156.4, 164.9] | 53.7 [48.8, 59.5] | 11.4 [10.5, 12.4] |
| Mozambique 2011 | 2,594 | 17.0 [15.0, 18.0] | 154.4 [150.2, 158.8] | 50.1 [45.4, 55.4] | 11.9 [10.8, 12.9] |
| Namibia 2013 | 776 | 17.0 [16.0, 18.0] | 159.7 [155.0, 163.9] | 50.8 [46.0, 57.3] | 13.3 [12.3, 14.1] |
| Niger 2012 | 706 | 17.0 [16.0, 18.0] | 158.2 [154.2, 162.0] | 49.5 [45.3, 54.5] | 12.2 [11.2, 13.2] |
| Nigeria 2018 | 2,559 | 17.0 [15.0, 18.0] | 157.0 [152.6, 160.8] | 50.0 [45.2, 55.7] | 11.6 [10.8, 12.5] |
| Rwanda 2014-15 | 1,345 | 17.0 [16.0, 18.0] | 155.3 [151.0, 160.0] | 53.0 [47.6, 58.8] | 13.2 [12.3, 14.0] |
| STP 2008-09 | 430 | 17.0 [16.0, 18.0] | 158.0 [154.0, 162.0] | 53.0 [49.0, 60.0] | 11.9 [10.9, 12.8] |
| Senegal 2010-11 | 1,215 | 17.0 [16.0, 18.0] | 162.0 [157.6, 166.4] | 51.2 [46.4, 56.8] | 11.9 [10.8, 12.8] |
| Sierra Leone 2013 | 1,612 | 17.0 [15.0, 18.0] | 156.4 [152.8, 160.4] | 51.0 [47.0, 56.0] | 12.0 [11.0, 13.0] |
| South Africa 2016 | 488 | 17.0 [16.0, 18.0] | 157.8 [153.6, 161.9] | 56.3 [49.9, 63.7] | 12.7 [11.5, 13.8] |
| Tanzania 2015-16 | 2,591 | 17.0 [15.0, 18.0] | 155.3 [151.2, 159.6] | 50.5 [45.6, 56.6] | 12.1 [11.2, 13.0] |
| Togo 2013-14 | 851 | 17.0 [16.0, 18.0] | 158.1 [153.7, 162.2] | 52.6 [47.5, 58.0] | 11.9 [10.9, 12.8] |
| Uganda 2016 | 1,230 | 17.0 [16.0, 18.0] | 157.3 [153.3, 161.8] | 52.1 [47.7, 57.5] | 12.6 [11.7, 13.5] |
| Zimbabwe 2015 | 1,859 | 17.0 [16.0, 18.0] | 159.0 [154.9, 163.0] | 53.9 [48.8, 60.1] | 12.9 [11.9, 13.9] |
| **Eastern Mediterranean region** | | | | | |
| Egypt 2014 | 145 | 18.0 [17.0, 19.0] | 159.1 [155.7, 162.3] | 62.8 [58.0, 69.8] | 12.6 [12.1, 13.1] |
| Jordan 2017-18 | 117 | 18.0 [17.0, 19.0] | 158.1 [154.6, 160.7] | 60.5 [54.4, 66.9] | 12.2 [11.1, 13.1] |
| Yemen 2013 | 1,733 | 17.0 [16.0, 18.0] | 153.0 [149.0, 157.0] | 45.5 [40.9, 50.3] | 10.9 [9.6, 12.1] |
| **European region** |  |  |  |  |  |
| Albania 2017-18 | 1,594 | 17.0 [16.0, 18.0] | 161.1 [158.0, 165.2] | 55.1 [50.5, 60.8] | 12.9 [12.1, 13.6] |
| Armenia 2015-16 | 677 | 17.0 [16.0, 18.0] | 158.2 [153.2, 161.9] | 52.5 [48.4, 58.1] | 13.2 [12.3, 14.0] |
| Azerbaijan 2006 | 1,375 | 17.0 [16.0, 18.0] | 158.2 [154.2, 161.5] | 52.0 [48.2, 57.5] | 12.6 [11.9, 13.4] |
| Kyrgyz Republic 2012 | 1,487 | 17.0 [16.0, 18.0] | 159.2 [155.0, 163.0] | 52.0 [48.0, 57.1] | 12.5 [11.7, 13.3] |
| Moldova 2005 | 1,330 | 17.0 [16.0, 18.0] | 162.0 [157.7, 166.0] | 54.0 [49.6, 59.6] | 12.8 [12.0, 13.5] |
| Tajikistan 2017 | 1,783 | 17.0 [16.0, 18.0] | 157.3 [153.5, 160.9] | 51.5 [46.9, 56.7] | 12.4 [11.6, 13.2] |
| **Americas region** |  |  |  |  |  |
| Bolivia 2008 | 1,080 | 17.0 [16.0, 18.0] | 153.5 [149.8, 157.1] | 53.4 [48.7, 58.6] | 12.5 [11.5, 13.5] |
| Guatemala 2014-15 | 5,237 | 17.0 [16.0, 18.0] | 149.5 [145.3, 153.9] | 49.8 [45.1, 55.5] | 13.5 [12.6, 14.2] |
| Guyana 2009 | 854 | 17.0 [16.0, 18.0] | 157.0 [152.2, 161.7] | 51.5 [45.5, 59.2] | 12.5 [11.5, 13.3] |
| Haiti 2016-17 | 2,127 | 17.0 [16.0, 18.0] | 158.7 [154.5, 162.9] | 51.7 [47.0, 57.0] | 11.9 [10.8, 12.8] |
| Honduras 2011-12 | 4,516 | 17.0 [16.0, 18.0] | 153.5 [149.5, 157.5] | 51.3 [46.2, 57.7] | 13.3 [12.5, 14.1] |
| Peru 2012 | 4,163 | 17.0 [16.0, 18.0] | 152.6 [148.9, 156.2] | 52.5 [47.7, 57.6] | 13.1 [12.3, 13.8] |
| **Southeast Asian region** |  |  |  |  |  |
| India 2015-16 | 116,665 | 17.0 [16.0, 18.0] | 151.4 [147.8, 155.4] | 43.9 [39.9, 48.6] | 11.9 [10.8, 12.8] |
| Maldives 2016-17 | 880 | 17.0 [16.0, 18.0] | 153.8 [150.5, 157.7] | 47.2 [41.0, 57.0] | 11.7 [10.8, 12.5] |
| Myanmar 2015-16 | 1,744 | 17.0 [16.0, 18.0] | 152.2 [148.7, 155.8] | 46.3 [42.4, 50.6] | 12.1 [11.2, 13.0] |
| Nepal 2016 | 1,216 | 17.0 [16.0, 18.0] | 152.3 [148.4, 155.8] | 46.0 [42.3, 50.3] | 12.1 [11.2, 13.0] |
| Timor-Leste 2016 | 1,008 | 17.0 [16.0, 18.0] | 150.8 [147.3, 154.9] | 43.1 [39.8, 47.3] | 12.8 [12.1, 13.5] |
| **Western Pacific region** |  |  |  |  |  |
| Cambodia 2014 | 1,796 | 17.0 [16.0, 18.0] | 153.0 [149.7, 156.6] | 46.6 [42.6, 50.7] | 12.1 [11.2, 12.8] |

Values are medians and interquartile ranges, unless otherwise indicated.

Hb, Haemoglobin levels; DRC, Democratic Republic of the Congo; STP, Sao Tome and Principe.

**Supplemental Table 3.** Characteristics of children (6-59 months) included in the study.

|  | **Children (6-59 months)** | | | | | |
| --- | --- | --- | --- | --- | --- | --- |
| **Country and survey year** | **Sample size,** *n* | **Age, months** | **Girls*, %** | **Height, cm** | **Weight, kg** | **Hb, g/dL** |
| **African region** |  |  |  |  |  |  |
| Angola 2015-16 | 5,319 | 31.5 [18.3, 44.6] | 49.8 (47.8, 51.7) | 85.3 [76.5, 94.4] | 11.6 [9.4, 13.8] | 10.5 [9.6, 11.4] |
| Benin 2017-18 | 5,343 | 30.7 [17.5, 45.1] | 49.1 (47.7, 50.6) | 85.2 [76.3, 94.4] | 11.4 [9.2, 13.7] | 10.2 [9.2, 11.1] |
| Burkina Faso 2010 | 5,685 | 31.1 [18.2, 44.8] | 49.2 (47.8, 50.6) | 85.5 [76.6, 94.5] | 11.2 [9.0, 13.6] | 9.1 [7.9, 10.2] |
| Burundi 2016-17 | 5,334 | 31.6 [18.1, 46.7] | 49.9 (48.4, 51.4) | 83.9 [75.2, 91.8] | 11.1 [9.0, 13.0] | 10.6 [9.5, 11.6] |
| Cameroon 2011 | 4,394 | 29.3 [16.2, 43.7] | 51.4 (49.7, 53.0) | 84.6 [75.2, 94.1] | 11.8 [9.5, 14.3] | 10.5 [9.5, 11.5] |
| Congo 2011-12 | 3,888 | 30.9 [17.1, 43.3] | 51.2 (48.7, 53.4) | 86.4 [76.5, 95.2] | 11.7 [9.5, 13.9] | 10.5 [9.6, 11.3] |
| Cote d'Ivoire 2011-12 | 2,685 | 30.0 [17.1, 43.0] | 52.5 (50.1, 54.8) | 85.1 [76.4, 93.6] | 11.5 [9.3, 13.8] | 9.9 [8.9, 10.9] |
| DRC 2013-14 | 6,856 | 31.2 [17.6, 44.8] | 50.5 (49.0, 52.0) | 84.6 [76.5, 92.1] | 11.3 [9.3, 13.3] | 10.4 [9.3, 11.5] |
| Eswatini 2006-07 | 1,730 | 29.1 [16.3, 44.1] | 50.5 (48.0, 53.1) | 85.3 [75.4, 94.7] | 12.4 [10.0, 14.7] | 11.2 [10.2, 12.1] |
| Ethiopia 2016 | 7,426 | 31.6 [17.8, 46.4] | 48.5 (46.9, 50.1) | 85.6 [76.7, 94.4] | 11.3 [9.1, 13.3] | 10.5 [9.4, 11.6] |
| Gabon 2012 | 2,903 | 29.3 [17.0, 43.5] | 49.9 (46.5, 53.2) | 86.4 [76.2, 95.7] | 12.1 [9.8, 14.5] | 10.5 [9.6, 11.3] |
| Gambia 2013 | 2,614 | 30.3 [16.7, 43.9] | 47.8 (45.5, 50.0) | 85.7 [76.4, 95.6] | 11.3 [9.1, 13.4] | 9.9 [8.8, 10.9] |
| Ghana 2014 | 2,359 | 30.7 [18.5, 44.2] | 47.1 (44.7, 49.5) | 86.9 [78.4, 96.2] | 11.9 [9.6, 14.2] | 10.3 [9.1, 11.2] |
| Guinea 2018 | 2,867 | 33.8 [17.8, 45.4] | 48.4 (46.5, 50.4) | 87.2 [77.3, 96.1] | 11.9 [9.7, 14.1] | 10.1 [9.2, 11.0] |
| Lesotho 2014 | 1,127 | 29.3 [16.0, 44.1] | 53.7 (50.5, 56.8) | 84.1 [75.4, 93.4] | 11.6 [9.6, 14.0] | 10.8 [9.8, 11.8] |
| Madagascar 2003-04 | 1,383 | 29.1 [14.0, 45.1] | 52.0 (48.7, 55.2) | 83.4 [71.3, 92.9] | 10.5 [8.1, 12.8] | 10.5 [9.8, 11.2] |
| Malawi 2015-16 | 4,533 | 32.6 [18.7, 45.8] | 51.8 (50.1, 53.5) | 86.2 [77.5, 94.5] | 11.9 [9.8, 14.0] | 10.5 [9.5, 11.4] |
| Mali 2018 | 3,615 | 31.1 [18.4, 44.8] | 48.8 (46.9, 50.7) | 87.2 [77.8, 96.4] | 11.6 [9.3, 13.9] | 9.7 [8.6, 10.7] |
| Mozambique 2011 | 4,152 | 30.3 [16.8, 44.5] | 51.2 (49.3, 53.0) | 85.0 [75.1, 93.8] | 11.9 [9.4, 14.1] | 10.3 [9.3, 11.3] |
| Namibia 2013 | 1,488 | 28.6 [16.2, 42.8] | 51.2 (48.6, 53.8) | 85.0 [76.1, 95.0] | 11.5 [9.3, 13.8] | 10.9 [9.9, 11.8] |
| Niger 2012 | 3,969 | 32.0 [18.9, 45.3] | 49.8 (48.2, 51.5) | 85.2 [76.0, 93.9] | 10.8 [8.7, 13.0] | 10.0 [9.0, 11.0] |
| Nigeria 2018 | 9,884 | 31.2 [17.9, 45.4] | 49.0 (47.8, 50.1) | 85.4 [76.5, 95.0] | 11.6 [9.2, 13.9] | 10.3 [9.2, 11.2] |
| Rwanda 2014-15 | 3,165 | 31.1 [17.7, 43.7] | 49.2 (47.3, 51.2) | 85.1 [76.0, 93.5] | 12.0 [10.0, 14.2] | 11.4 [10.5, 12.2] |
| STP 2008-09 | 1,301 | 31.1 [18.9, 43.8] | 50.6 (46.7, 54.4) | 88.0 [78.0, 97.0] | 12.0 [10.0, 14.0] | 10.6 [9.8, 11.4] |
| Senegal 2010-11 | 2,933 | 29.6 [16.6, 43.8] | 48.4 (46.0, 50.9) | 85.5 [75.8, 95.1] | 11.3 [9.1, 13.3] | 9.8 [8.7, 10.8] |
| Sierra Leone 2013 | 3,524 | 30.9 [17.2, 44.3] | 51.1 (49.0, 53.3) | 86.0 [76.1, 95.5] | 12.0 [9.5, 14.2] | 9.8 [8.6, 10.7] |
| South Africa 2016 | 781 | 33.5 [19.2, 46.8] | 51.4 (47.1, 55.6) | 89.3 [78.2, 97.4] | 13.0 [10.8, 15.4] | 10.5 [9.4, 11.4] |
| Tanzania 2015-16 | 7,885 | 30.0 [17.4, 44.6] | 49.4 (48.2, 50.7) | 85.1 [76.2, 94.2] | 11.6 [9.4, 13.7] | 10.6 [9.7, 11.6] |
| Togo 2013-14 | 2,828 | 31.0 [17.6, 45.0] | 49.9 (47.8, 52.0) | 86.1 [77.8, 94.3] | 11.6 [9.3, 13.7] | 10.1 [9.1, 11.2] |
| Uganda 2016 | 3,877 | 30.9 [18.3, 45.3] | 49.8 (48.1, 51.5) | 86.5[77.1, 95.5] | 12.1 [9.8, 14.4] | 10.8 [9.7, 11.7] |
| Zimbabwe 2015 | 4,065 | 32.0 [18.7, 46.1] | 50.7 (49.0, 52.4) | 87.3 [77.5, 96.5] | 12.4 [9.9, 14.7] | 11.3 [10.5, 12.1] |
| **Eastern Mediterranean region** | | | | | | |
| Egypt 2014 | 4,166 | 31.5 [18.5, 45.1] | 47.8 (45.9, 49.7) | 89.2 [79.3, 98.8] | 13.0 [10.3, 15.4] | 11.5 [10.8, 12.1] |
| Jordan 2012 | 5,408 | 33.0 [19.7, 46.4] | 48.7 (46.7, 50.7) | 91.0 [81.1, 99.5] | 13.2 [10.9, 15.4] | 11.6 [10.7, 12.5] |
| Yemen 2013 | 3,433 | 31.7 [18.0, 45.8] | 50.2 (48.1, 52.3) | 84.4 [76.0, 92.2] | 10.8 [8.9, 12.5] | 8.7 [7.4, 9.9] |
| **European region** |  |  |  |  |  |  |
| Albania 2017-18 | 1,890 | 32.8 [20.5, 46.3] | 48.3 (45.5, 51.1) | 90.6 [81.4, 100] | 14.0 [11.6, 16.3] | 11.5 [10.8, 12.3] |
| Armenia 2015-16 | 1,302 | 33.5 [19.4, 46.8] | 47.4 (44.7, 50.1) | 92.3 [81.5, 100.8] | 13.9 [11.5, 16.3] | 12.0 [11.3, 12.9] |
| Azerbaijan 2006 | 1,665 | 32.1 [18.8, 45.6] | 47.1 (44.5, 49.8) | 86.7 [78.4, 95.7] | 12.5 [10.3, 14.8] | 11.4 [10.6, 12.1] |
| Kyrgyz Republic 2012 | 3,531 | 30.2 [17.1, 43.9] | 48.9 (47.1, 50.7) | 87.4 [77.9, 96.1] | 12.6 [10.3, 14.9] | 11.1 [10.1, 12.0] |
| Moldova 2005 | 1,083 | 29.5 [17.1, 44.0] | 48.5 (45.2, 51.8) | 89.7 [79.2, 98.5] | 12.9 [10.7, 15.2] | 11.4 [10.7, 12.1] |
| Tajikistan 2017 | 5,199 | 32.4 [19.5, 45.8] | 49.6 (47.7, 51.5) | 88.7 [79.4, 97.2] | 12.5 [10.2, 14.7] | 11.2 [10.3, 12.1] |
| **Americas region** |  |  |  |  |  |  |
| Bolivia 2008 | 2,285 | 31.8 [18.4, 45.4] | 48.4 (46.0, 50.8) | 87.0 [77.3, 95.2] | 12.7 [10.2, 15.0] | 10.6 [9.4, 11.6] |
| Guatemala 2014-15 | 10,287 | 32.4 [19.1, 45.8] | 48.5 (47.4, 49.6) | 85.4 [76.3, 93.4] | 11.9 [9.7, 14.0] | 11.5 [10.6, 12.3] |
| Guyana 2009 | 1,308 | 30.9 [18.7, 45.4] | 50.9 (47.4, 54.3) | 87.9 [78.0, 97.2] | 12.3 [10.1, 14.7] | 11.3 [10.3, 12.1] |
| Haiti 2016-17 | 4,881 | 32.6 [18.5, 46.5] | 49.6 (47.8, 51.4) | 88.0 [78.5, 97.2] | 12.3 [10.0,14.7] | 10.4 [9.4, 11.3] |
| Honduras 2011-12 | 8,442 | 30.9 [18.0, 44.9] | 47.7 (46.4, 48.9) | 86.5 [77.8, 95.1] | 12.1 [10.0, 14.4] | 11.6 [10.7, 12.3] |
| Peru 2012 | 8,194 | 33.0 [19.8, 46.2] | 48.7 (47.3, 50.2) | 88.6 [79.0, 96.7] | 12.8 [10.4, 15.1] | 11.4 [10.6, 12.2] |
| **Southeast Asian region** |  |  |  |  |  |  |
| India 2015-16 | 196,301 | 33.1 [19.4, 46.2] | 48.0 (47.7, 48.4) | 86.9 [77.5, 95.0] | 11.1 [9.1, 13.1] | 10.7 [9.7, 11.6] |
| Maldives 2016-17 | 1,859 | 34.6 [20.9, 46.6] | 46.8 (43.4, 50.2) | 91.0 [80.5, 99.3] | 12.1 [10.1, 14.3] | 11.0 [10.2, 11.9] |
| Myanmar 2015-16 | 3,436 | 33.0 [19.0, 46.0] | 48.6 (46.4, 50.9) | 87.0 [78.3, 95.0] | 11.5 [9.6, 13.4] | 10.8 [9.9, 11.6] |
| Nepal 2016 | 2,044 | 32.7 [19.9, 46.0] | 48.1 (45.8, 50.4) | 86.1 [77.7, 94.5] | 11.2 [9.2, 13.2] | 10.9 [9.9, 11.7] |
| Timor-Leste 2016 | 1,537 | 33.7 [20.0, 46.6] | 47.9 (45.0, 50.9) | 87.0 [76.8, 95.0] | 10.9 [9.0, 12.7] | 11.2 [10.3, 12.0] |
| **Western Pacific region** |  |  |  |  |  |  |
| Cambodia 2014 | 3,799 | 31.2 [18.2, 45.6] | 49.1 (47.0, 51.2) | 86.2 [76.5, 94.4] | 11.1 [9.2, 13.0] | 10.8 [9.9, 11.6] |

Hb, Haemoglobin levels; DRC, Democratic Republic of the Congo; STP, Sao Tome and Principe.

*Values for girls are percentages and 95% CIs; estimates account for survey design. All other values (i.e., age, height, weight and hb) are medians and interquartile ranges.

**Supplemental Table 4.** Prevalence of overweight/obesity, anaemia and concurrent overweight/obesity and anaemia among adult women, adolescent girls and children.

|  | **Adult women (20-49 years old)^*^** | | | **Adolescent girls (15-19 years old)^*^** | | | **Children (6-59 months)^*^** | | |
| --- | --- | --- | --- | --- | --- | --- | --- | --- | --- |
| **Country** | **OWOB** | **Anaemia** | **DBM** | **OWOB** | **Anaemia** | **DBM** | **OWOB** | **Anaemia** | **DBM** |
| **African region** | **31.1 [26.4, 35.8]** | **41.1 [36.7, 45.5]** | **11.1 [9.2, 13.0]** | **9.9 [8.2, 11.5]** | **42.2 [37.3, 47.1]** | **5.1 [4.2, 6.0]** | **5.8 [4.9, 6.6]** | **64.3 [59.9, 68.6]** | **3.3 [2.8, 3.8]** |
| Angola 2015-16 | - | - | - | - | - | - | 4.1 [3.4, 4.9] | 65.1 [62.7, 67.3] | 2.3 [1.8, 2.9] |
| Benin 2017-18 | 31.4 [29.7, 33.1] | 55.7 [54.0, 57.4] | 15.7 [14.6, 16.9] | 9.4 [7.9, 11.1] | 56.6 [53.6, 59.6] | 4.9 [3.9, 6.2] | 2.0 [1.7, 2.3] | 71.6 [70.0, 73.2] | 1.3 [1.0, 1.7] |
| Burkina Faso 2010 | 13.1 [11.9, 14.3] | 47.5 [45.7, 49.3] | 5.3 [4.7, 6.0] | 6.5 [4.9, 8.5] | 45.8 [42.9, 48.7] | 2.4 [1.6, 3.5] | 3.1 [2.6, 3.7] | 87.7 [86.6, 88.7] | 2.9 [2.4, 3.6] |
| Burundi 2016-17 | 8.9 [8.0, 9.9] | 39.1 [37.4, 40.8] | 2.2 [1.8, 2.6] | 6.2 [4.8, 7.9] | 35.4 [32.7, 38.2] | 1.7 [1.1, 2.6] | 1.8 [1.5, 2.3] | 60.4 [58.6, 62.3] | 1.0 [0.8, 1.4] |
| Cameroon 2011 | 37.4 [35.7, 39.1] | 37.8 [36.1, 39.5] | 13.3 [12.3, 14.4] | 19.8 [17.8, 22.0] | 38.2 [35.2, 41.2] | 7.3 [6.0, 8.8] | 8.3 [7.4, 9.3] | 61.6 [59.2, 63.1] | 4.5 [3.8, 5.2] |
| Congo 2011-12 | 31.6 [29.1, 34.2] | 53.7 [51.1, 56.2] | 15.9 [13.8, 18.1] | 8.4 [6.1, 11.4] | 54.3 [48.8, 59.7] | 3.0 [1.7, 5.1] | 3.9 [3.0, 5.1] | 67.2 [64.4, 69.9] | 2.7 [2.0, 3.7] |
| Cote d'Ivoire 2011-12 | 30.1 [27.9, 32.4] | 52.1 [49.5, 54.7] | 13.8 [12.1, 15.6] | 14.7 [11.9, 18.0] | 52.6 [47.7, 57.3] | 9.1 [6.7, 12.3] | 3.9 [3.1, 4.8] | 75.5 [73.4, 77.5] | 2.9 [2.2, 3.7] |
| DRC 2013-14 | 18.5 [16.4, 20.8] | 36.9 [34.5, 39.4] | 5.7 [4.9, 6.6] | 9.4 [7.5, 11.8] | 39.0 [35.5, 42.7] | 3.0 [2.1, 4.4] | 4.8 [4.1, 5.6] | 59.8 [57.6, 62.0] | 2.4 [2.0, 3.0] |
| Eswatini 2006-07 | 59.9 [57.9, 61.8] | 29.6 [27.9, 31.3] | 16.3 [14.9, 17.8] | 30.3 [27.7, 33.0] | 26.4 [23.9, 28.9] | 8.2 [6.5, 10.3] | 14.2 [12.6, 15.9] | 42.3 [39.2, 45.4] | 7.0 [6.0, 8.1] |
| Ethiopia 2016 | 8.9 [7.7, 10.3] | 23.6 [21.8, 25.4] | 1.7 [1.3, 2.1] | 5.1 [4.0, 6.5] | 19.5 [17.4, 21.7] | 1.0 [0.6, 1.6] | 2.9 [2.4, 3.6] | 57.0 [54.5, 59.5] | 1.3 [1.0, 1.8] |
| Gabon 2012 | 51.9 [49.1, 54.6] | 60.5 [57.6, 63.3] | 30.1 [27.6, 32.8] | 18.0 [14.7, 21.8] | 62.6 [58.0, 66.9] | 10.9 [8.1, 14.5] | 9.2 [7.4, 11.3] | 61.2 [58.4, 64.0] | 6.0 [4.6, 7.8] |
| Gambia 2013 | 27.0 [25.1, 29.0] | 59.2 [56.3, 62.1] | 13.7 [12.2, 15.3] | 11.6 [9.2, 14.4] | 57.7 [53.0, 62.2] | 6.2 [4.4, 8.6] | 2.7 [2.1, 3.5] | 73.0 [70.0, 75.7] | 1.6 [1.1, 2.4] |
| Ghana 2014 | 47.2 [44.8, 49.7] | 40.7 [38.5, 43.0] | 17.0 [15.2, 19.0] | 11.9 [9.6, 14.7] | 46.5 [42.6, 50.5] | 5.4 [3.9, 7.5] | 2.8 [2.1, 3.8] | 66.7 [63.6, 69.7] | 1.9 [1.4, 2.7] |
| Guinea 2018 | 32.0 [29.8, 34.3] | 44.1 [42.1, 46.2] | 12.7 [11.4, 14.1] | 14.7 [12.3, 17.4] | 46.6 [43.4, 49.9] | 7.3 [5.8, 9.2] | 6.3 [5.3, 7.4] | 75.1 [73.1, 77.0] | 4.7 [3.8, 5.9] |
| Lesotho 2014 | 51.8 [49.3, 54.2] | 27.2 [25.2, 29.3] | 12.4 [10.9, 14.1] | 22.5 [18.7, 26.9] | 24.1 [19.8, 29.0] | 6.9 [4.9, 9.7] | 9.5 [8.0, 11.4] | 54.2 [49.9, 58.4] | 4.4 [3.2, 5.9] |
| Madagascar 2003-04 | - | - | - | - | - | - | 5.5 [4.2, 7.2] | 68.3 [64.2, 72.2] | 3.1 [1.9, 5.0] |
| Madagascar 2008-09 | 7.9 [6.9, 8.9] | 35.3 [33.6, 37.1] | 2.2 [1.8, 2.7] | 1.6 [1.0, 2.6] | 34.6 [31.8, 37.6] | 0.5 [0.3, 0.9] | - | - | - |
| Malawi 2015-16 | 24.5 [23.1, 26.0] | 30.5 [28.9, 32.1] | 6.7 [6.0, 7.6] | 10.3 [8.5, 12.4] | 33.2 [30.3, 36.3] | 3.2 [2.3, 4.6] | 5.3 [4.5, 6.2] | 63.0 [61.0, 65.0] | 3.3 [2.8, 4.0] |
| Mali 2018 | 31.7 [29.3, 34.3] | 61.8 [59.4, 64.2] | 15.8 [14.3, 17.4] | 13.3 [10.6, 16.5] | 64.4 [60.0, 68.6] | 6.4 [4.7, 8.7] | 2.0 [1.7, 2.4] | 82.4 [80.8, 83.9] | 1.6 [1.2, 2.1] |
| Mozambique 2011 | 19.3 [18.1, 20.5] | 53.4 [51.6, 55.2] | 8.4 [7.6, 9.2] | 1.6 [1.1, 2.3] | 55.2 [52.4, 58.0] | 5.3 [4.4, 6.4] | 9.4 [8.5, 10.3] | 68.7 [66.4, 70.8] | 6.1 [5.2, 7.0] |
| Namibia 2013 | 37.9 [36.0, 39.9] | 20.4 [18.7, 22.2] | 6.8 [5.8, 7.9] | 10.1 [7.8, 13.1] | 18.4 [15.5, 21.9] | 1.6 [0.8, 3.4] | 5.1 [4.1, 6.4] | 49.2 [46.1, 52.2] | 2.4 [1.6, 3.4] |
| Niger 2012 | 20.4 [18.7, 22.3] | 42.2 [39.7, 44.7] | 7.7 [6.6, 8.9] | 5.6 [4.1, 7.7] | 43.3 [38.7, 48.0] | 1.6 [0.9, 2.8] | 2.8 [2.2, 3.5] | 73.0 [71.0, 74.9] | 1.7 [1.2, 2.3] |
| Nigeria 2018 | 33.2 [31.9, 34.5] | 56.3 [54.9, 57.6] | 16.6 [15.6, 17.7] | 10.7 [9.2, 12.4] | 59.8 [57.5, 62.1] | 6.4 [5.1, 7.9] | 2.4 [2.1, 2.8] | 67.9 [66.4, 69.3] | 1.5 [1.2, 1.8] |
| Rwanda 2014-15 | 22.9 [21.6, 24.3] | 18.6 [17.2, 20.0] | 3.4 [3.0, 4.0] | 17.5 [15.5, 19.7] | 18.5 [16.3, 21.0] | 3.0 [2.2, 4.0] | 10.9 [9.9, 12.0] | 36.5 [34.5, 38.5] | 3.3 [2.7, 4.0] |
| STP 2008-09 | 39.1 [36.2, 42.1] | 38.9 [35.5, 42.5] | 14.5 [11.9, 17.7] | 15.7 [12.4, 19.5] | 50.2 [43.0, 57.4] | 7.6 [5.2, 10.8] | 13.2 [11.1, 15.7] | 63.3 [59.6, 66.8] | 8.6 [6.9, 10.5] |
| Senegal 2010-11 | 26.0 [23.6, 28.5] | 53.4 [50.9, 55.9] | 12.8 [11.1, 14.7] | 8.1 [6.1, 10.6] | 54.6 [51.2, 58.0] | 5.1 [3.4, 7.5] | 2.9 [2.2, 3.7] | 77.1 [74.8, 79.3] | 1.7 [1.2, 2.4] |
| Sierra Leone 2013 | 21.6 [19.8, 23.5] | 42.0 [39.3, 44.7] | 8.4 [7.4, 9.6] | 10.7 [9.0, 12.8] | 48.0 [44.7, 51.4] | 5.1 [3.9, 6.7] | 9.9 [8.7, 11.3] | 80.1 [78.3, 81.7] | 8.0 [6.8, 9.4] |
| South Africa 2016 | 69.0 [66.5, 71.3] | 32.2 [29.1, 35.6] | 20.7 [17.8, 23.9] | 32.0 [27.4, 36.9] | 33.0 [28.2, 38.2] | 10.6 [7.8, 14.1] | 15.7 [12.9, 18.9] | 62.3 [57.6, 66.8] | 9.1 [6.7, 12.1] |
| Tanzania 2015-16 | 33.7 [32.0, 35.4] | 42.3 [40.6, 43.9] | 12.0 [11.1, 12.9] | 14.3 [12.5, 16.2] | 45.0 [42.6, 47.6] | 6.5 [5.2, 8.1] | 4.8 [4.3, 5.4] | 58.6 [56.9, 60.2] | 2.8 [2.4, 3.3] |
| Togo 2013-14 | 35.5 [33.6, 37.5] | 44.7 [42.6, 46.8] | 14.7 [13.3, 16.2] | 15.0 [12.4, 18.1] | 53.8 [50.0, 57.5] | 8.1 [6.1, 10.5] | 2.1 [1.6, 2.8] | 70.8 [68.6, 72.9] | 1.6 [1.2, 2.3] |
| Uganda 2016 | 28.0 [26.0, 30.0] | 29.5 [27.7, 31.4] | 6.4 [5.5, 7.5] | 13.7 [11.6, 16.2] | 31.5 [28.6, 34.6] | 3.9 [2.8, 5.5] | 5.0 [4.3, 5.7] | 53.6 [51.3, 55.9] | 2.6 [2.1, 3.3] |
| Zimbabwe 2015 | 41.2 [39.5, 43.0] | 26.3 [24.9, 27.7] | 9.9 [9.0, 10.9] | 16.4 [14.7, 18.3] | 25.6 [23.4, 27.9] | 4.4 [3.3, 5.7] | 6.8 [6.0, 7.7] | 38.2 [36.3, 40.1] | 2.5 [2.0, 3.2] |
| **Eastern Mediterranean region** | **61.5 [24.8, 98.3]** | **47.8 [19.0, 76.7]** | **23.8 [17.0, 30.7]** | **31.4 [0.0, 64.0]** | **46.0 [10.3, 81.8]** | **11.8 [4.2, 19.3]** | **7.3 [0.6, 14.1]** | **49.7 [10.0, 89.3]** | **2.5 [1.3, 3.7]** |
| Egypt 2014 | 85.5 [84.7, 86.2] | 25.6 [24.1, 27.2] | 21.4 [20.0, 22.8] | 54.2 [48.8, 59.6] | 22.2 [15.0, 31.6] | 12.9 [7.4, 21.7] | 16.9 [15.8, 17.9] | 27.8 [25.9, 29.8] | 4.6 [3.8, 5.5] |
| Jordan 2012 | - | - | - | - | - | - | 4.6 [3.8, 5.5] | 32.2 [29.8, 34.6] | 1.4 [1.0, 2.1] |
| Jordan 2017-18 | 70.0 [68.0, 71.9] | 44.7 [42.4, 47.0] | 30.9 [29.1, 32.8] | 38.8 [27.4, 51.6] | 39.6 [28.2, 52.3] | 21.5 [12.8, 33.8] | - | - | - |
| Yemen 2013 | 29.8 [28.4, 31.3] | 70.0 [68.0, 71.9] | 18.5 [16.9, 20.1] | 8.2 [7.4, 9.3] | 68.5 [65.4, 71.4] | 5.4 [4.1, 7.0] | 2.4 [2.1, 2.8] | 86.0 [84.4, 87.5] | 2.0 [1.5, 2.6] |
| **European region** | **50.5 [44.2, 56.7]** | **29.4 [20.6, 38.2]** | **13.1 [9.4, 16.8]** | **9.2 [7.5, 10.8]** | **25.6 [20.1, 31.1]** | **2.5 [1.5, 3.4]** | **12.1 [7.5, 16.7]** | **32.6 [24.3, 40.9]** | **3.8 [2.4, 5.2]** |
| Albania 2017-18 | 60.5 [59.1, 61.9] | 21.7 [20.5, 22.9] | 12.6 [11.8, 13.5] | 17.4 [14.9, 20.2] | 17.7 [15.3, 20.4] | 2.6 [2.3, 5.6] | 18.0 [16.0, 20.2] | 24.1 [21.6, 26.8] | 4.3 [3.3, 5.6] |
| Armenia 2015-16 | 49.8 [48.1, 51.5] | 12.9 [11.5, 14.5] | 5.7 [4.9, 6.7] | 10.6 [8.2, 13.6] | 17.2 [13.9, 21.2] | 0.6 [0.2, 1.7] | 14.7 [12.4, 17.2] | 15.8 [13.7, 18.2] | 3.3 [2.3, 4.6] |
| Azerbaijan 2006 | 55.7 [54.1, 57.4] | 37.3 [35.6, 39.0] | 19.7 [18.2, 21.3] | 12.2 [10.2, 14.5] | 28.9 [26.0, 32.1] | 3.6 [2.4, 5.2] | 14.8 [12.8, 16.9] | 37.5 [34.0, 41.2] | 5.2 [4.0, 6.7] |
| Kyrgyz Republic 2012 | 43.7 [41.8, 45.7] | 34.7 [32.8, 36.7] | 12.5 [11.4, 13.7] | 8.6 [7.0, 10.4] | 34.4 [31.2, 37.8] | 2.3 [1.6, 3.2] | 10.3 [9.1, 11.7] | 43.2 [40.6, 45.8] | 5.0 [4.2, 6.0] |
| Moldova 2005 | 49.6 [48.0, 51.2] | 28.5 [26.9, 30.1] | 13.3 [12.4, 14.3] | 11.2 [9.5, 13.1] | 23.4 [21.0, 25.8] | 2.7 [1.9, 3.7] | 10.1 [8.4, 12.1] | 33.1 [30.2, 36.2] | 3.1 [2.0, 4.7] |
| Tajikistan 2017 | 43.4 [41.8, 45.0] | 42.3 [40.4, 44.2] | 16.5 [15.3, 17.8] | 12.7 [11.0, 14.7] | 33.3 [30.3, 36.3] | 3.3 [2.4, 4.6] | 4.0 [3.4, 4.7] | 41.6 [39.3, 43.9] | 1.8 [1.3, 2.1] |
| **Americas region** | **54.8 [48.6, 61.0]** | **27.9 [19.7, 36.0]** | **13.5 [10.5, 16.4]** | **18.7 [13.8, 23.6]** | **26.6 [17.1, 36.2]** | **5.1 [3.8, 6.4]** | **6.6 [5.0, 8.3]** | **44.0 [32.5, 55.4]** | **2.7 [1.9, 3.5]** |
| Bolivia 2008 | 56.8 [55.5, 58.1] | 36.5 [34.6, 38.5] | 18.5 [17.0, 20.1] | 29.2 [27.0, 31.4] | 36.0 [32.5, 39.7] | 10.3 [8.2, 12.8] | 11.3 [10.4, 12.2] | 60.7 [57.9, 63.5] | 6.7 [5.5, 8.2] |
| Guatemala 2014-15 | 60.6 [59.6, 61.7] | 13.2 [12.5, 13.9] | 7.3 [6.8, 7.8] | 28.9 [27.3, 30.5] | 10.4 [9.4, 11.5] | 2.8 [2.3, 3.3] | 5.1 [4.6, 5.5] | 32.7 [31.4, 34.2] | 1.2 [1.0, 1.5] |
| Guyana 2009 | 56.1 [53.8, 58.4] | 38.4 [36.3, 40.5] | 21.7 [19.9, 23.6] | 21.4 [17.9, 25.3] | 33.9 [29.7, 38.3] | 7.6 [5.7, 10.0] | 7.1 [5.5, 9.1] | 38.9 [35.2, 42.7] | 2.8 [1.8, 4.1] |
| Haiti 2016-17 | 38.5 [37.0, 40.1] | 47.0 [45.3, 48.7] | 16.6 [15.3, 17.9] | 11.2 [9.7, 12.8] | 52.7 [49.9, 55.4] | 6.0 [4.8, 7.4] | 3.9 [3.3, 4.5] | 66.8 [64.8, 68.8] | 2.3 [1.9, 2.9] |
| Honduras 2011-12 | 59.9 [58.8, 60.9] | 15.5 [14.7, 16.2] | 9.1 [8.5, 9.7] | 25.3 [23.7, 26.9] | 12.0 [10.8, 13.3] | 2.6 [2.1, 3.2] | 5.7 [5.1, 6.3] | 29.2 [27.9, 30.6] | 1.3 [1.0, 1.7] |
| Peru 2012 | 61.7 [60.7, 62.8] | 16.8 [16.0, 17.7] | 9.4 [8.8, 10.0] | 28.2 [26.4, 30.1] | 15.8 [14.4, 17.3] | 3.8 [3.1, 4.7] | 8.3 [7.4, 9.2] | 32.2 [30.8, 33.7] | 2.5 [2.1, 3.0] |
| **Southeast Asian region** | **28.3 [19.2, 37.4]** | **42.7 [33.0, 52.4]** | **12.1 [7.2, 17.0]** | **5.7 [3.9, 7.6]** | **43.4 [31.7, 55.2]** | **3.0 [1.8, 4.1]** | **3.1 [2.0, 4.1]** | **50.2 [44.6, 55.8]** | **1.2 [0.8, 1.7]** |
| India 2015-16 | 24.3 [24.1, 24.5] | 52.9 [52.6, 53.1] | 11.4 [11.2, 11.6] | 5.3 [5.1, 5.5] | 54.1 [53.6, 54.5] | 2.4 [2.2, 2.5] | 2.2 [2.1, 2.3] | 58.1 [57.7, 58.5] | 1.2 [1.1, 1.2] |
| Maldives 2016-17 | 55.0 [53.0, 57.0] | 63.6 [61.5, 65.6] | 33.6 [31.7, 35.6] | 18.5 [15.3, 22.2] | 59.8 [55.6, 64.0] | 10.2 [7.5, 13.6] | 4.7 [3.8, 5.8] | 50.8 [47.9, 53.7] | 1.8 [1.2, 2.8] |
| Myanmar 2015-16 | 28.2 [27.0, 29.6] | 45.7 [44.2, 47.2] | 10.7 [9.8, 11.6] | 6.2 [4.8, 8.1] | 47.5 [44.3, 50.6] | 2.2 [1.3, 3.6] | 1.5 [1.1, 2.1] | 59.0 [56.7, 61.2] | 1.0 [0.7, 1.6] |
| Nepal 2016 | 26.9 [24.7, 29.1] | 39.5 [37.4, 41.8] | 8.0 [7.0, 9.2] | 5.2 [4.0, 6.8] | 43.6 [39.6, 47.6] | 2.0 [1.3, 3.1] | 1.1 [0.7, 1.6] | 51.9 [49.2, 54.7] | 0.4 [0.2, 0.9] |
| Timor-Leste 2016 | 12.5 [11.4, 13.7] | 21.7 [19.8, 23.7] | 2.5 [1.9, 3.3] | 2.7 [2.1, 3.6] | 20.7 [17.5, 24.3] | 0.5 [0.2, 1.3] | 4.8 [4.1, 5.5] | 40.4 [37.2, 43.8] | 1.7 [1.0, 2.7] |
| **Western Pacific region** | **-** | **-** | **-** | **-** | **-** | **-** | **-** | **-** | **-** |
| Cambodia 2014 | 21.1 [19.9, 22.3] | 43.8 [42.4, 45.1] | 7.7 [6.9, 8.4] | 4.5 [3.5, 5.8] | 48.6 [45.7, 51.5] | 1.6 [1.1, 2.5] | 2.3 [1.8, 3.0] | 56.4 [54.3, 58.6] | 1.4 [0.9, 2.0] |
| **Overall pooled prevalence** | **37.5 [32.0, 43.0]** | **38.7 [34.2, 43.3]** | **12.4 [11.1, 13.7]** | **11.3 [9.9, 12.7]** | **38.8 [33.7, 43.9]** | **4.5 [4.0, 5.0]** | **6.3 [5.7, 7.0]** | **55.9 [51.7, 60.1]** | **3.0 [2.7, 3.3]** |

DRC, Democratic Republic of the Congo; STP, Sao Tome and Principe; OWOB, overweight/obesity; DBM, concurrent overweight/obesity and anaemia.

*Values are percentages and 95% CIs; estimates account for survey design. Regional estimates are pooled prevalences and 95% CIs, calculated with the available data from countries within that region. The Western Pacific region only had one country with available data (Cambodia), thus the regional prevalence was not calculated.

- We used the most recent dataset available for each country. Estimates were not calculated or are missing for that country due to i) data not available for one or more age groups (i.e. Angola) or ii) children’s anthropometric measures were unreliable in the most recent survey (i.e. Jordan and Madagascar).

**Supplemental Table 5.** Concurrent overweight/obesity and anaemia by household wealth quintiles among adult women (20-49 years old).

|  |  |  |  |  |  |  |  |
| --- | --- | --- | --- | --- | --- | --- | --- |
|  | **Household wealth quintiles^*^** | | | | | | |
| **Country** | **Poorest (Q1)** | **Poorer (Q2)** | **Middle (Q3)** | **Richer (Q4)** | **Richest (Q5)** | **Gap**^†^ | **p-value**^‡^ |
| **AFRO** | **5.9 [4.8, 7.0]** | **7.9 [6.4, 9.3]** | **10.4 [8.4, 12.3]** | **13.1 [10.7, 15.5]** | **16.8 [14.0, 19.6]** | **10.9** | **-** |
| Benin 2017-18 | 6.4 [4.8, 8.5] | 10.0 [8.1, 12.3] | 14.0 [11.8, 16.5] | 16.8 [14.3, 19.6] | 26.6 [23.9, 29.5] | 20.2 | 0.0000 |
| Burkina Faso 2010 | 2.8 [1.8, 4.2] | 2.2 [1.4, 3.5] | 3.1 [2.2, 4.5] | 4.3 [2.9, 6.2] | 12.1 [10.5, 13.7] | 9.3 | 0.0000 |
| Burundi 2016-17 | 1.0 [0.5, 2.0] | 1.5 [0.9, 2.4] | 1.5 [0.9, 2.6] | 2.4 [1.6, 3.7] | 4.3 [3.0, 6.2] | 3.3 | 0.0000 |
| Cameroon 2011 | 2.7 [1.6, 4.4] | 7.9 [6.3, 9.9] | 12.8 [10.6, 15.4] | 16.2 [13.8, 18.8] | 21.2 [18.9, 23.6] | 18.5 | 0.0000 |
| Congo 2011-12 | 6.2 [5.0, 7.8] | 10.0 [7.5, 13.0] | 15.5 [11.6, 20.4] | 21.0 [16.0, 27.2] | 25.3 [20.0, 31.4] | 19.1 | 0.0000 |
| Cote d'Ivoire 2011-12 | 4.6 [2.8, 7.6] | 10.5 [8.0, 13.8] | 14.9 [11.0, 19.9] | 16.2 [12.8, 20.2] | 20.4 [16.5, 24.9] | 15.8 | 0.0000 |
| DRC 2013-14 | 1.9 [1.3, 2.9] | 1.8 [1.1, 3.0] | 3.8 [2.7, 5.5] | 5.6 [3.6, 8.6] | 13.8 [11.6, 16.5] | 11.9 | 0.0000 |
| Eswatini 2006-07 | 9.7 [7.3, 12.6] | 11.9 [9.5, 14.7] | 16.2 [13.6, 19.2] | 19.0 [16.4, 21.9] | 21.2 [17.8, 25.0] | 11.5 | 0.0000 |
| Ethiopia 2016 | 1.5 [0.9, 2.4] | 0.7 [0.3, 1.5] | 0.5 [0.2, 1.0] | 1.1 [0.7, 2.0] | 3.7 [2.8, 4.9] | 2.2 | 0.0000 |
| Gabon 2012 | 17.3 [14.4, 20.7] | 28.3 [23.2, 34.0] | 30.5 [25.3, 36.1] | 35.5 [30.8, 40.6] | 35.0 [28.5, 42.2] | 17.7 | 0.0001 |
| Gambia 2013 | 11.7 [8.9, 15.3] | 8.7 [6.5, 11.5] | 15.3 [11.7, 19.7] | 17.2 [13.7, 21.4] | 14.6 [11.4, 18.6] | 2.9 | 0.0059 |
| Ghana 2014 | 5.5 [4.0, 7.6] | 14.3 [11.4, 17.7] | 16.7 [13.6, 20.4] | 18.2 [14.5, 22.5] | 25.2 [20.8, 30.2] | 19.7 | 0.0000 |
| Guinea 2018 | 5.2 [3.8, 7.1] | 11.2 [8.7, 14.5] | 10.7 [8.2, 13.8] | 17.3 [14.1, 21.0] | 18.8 [15.8, 22.2] | 13.6 | 0.0000 |
| Lesotho 2014 | 7.0 [4.6, 10.7] | 7.9 [5.4, 11.2] | 12.8 [9.6, 16.9] | 14.3 [11.0, 18.5] | 16.0 [12.7, 19.9] | 9.0 | 0.0008 |
| Madagascar 2008-09 | 1.0 [0.5, 2.0] | 1.0 [0.5, 2.0] | 1.2 [0.6, 2.7] | 2.4 [1.6, 3.6] | 4.6 [3.4, 6.3] | 3.6 | 0.0000 |
| Malawi 2015-16 | 4.2 [2.9, 5.9] | 3.3 [2.3, 4.8] | 5.7 [4.3, 7.4] | 7.9 [6.1, 10.0] | 11.6 [9.5, 13.9] | 7.4 | 0.0000 |
| Mali 2018 | 11.2 [8.6, 14.5] | 13.7 [11.1, 16.9] | 16.1 [11.4, 17.3] | 19.1 [15.9, 22.7] | 19.8 [16.4, 23.7] | 8.6 | 0.0004 |
| Mozambique 2011 | 3.0 [2.0, 4.4] | 3.7 [2.7, 5.0] | 5.7 [4.1, 7.8] | 8.5 [7.1, 10.1] | 18.1 [16.5, 19.7] | 15.1 | 0.0000 |
| Namibia 2013 | 3.4 [2.0, 5.7] | 4.2 [2.7, 6.5] | 5.3 [3.6, 7.7] | 10.0 [7.6, 13.0] | 8.9 [6.5, 12.1] | 5.5 | 0.0001 |
| Niger 2012 | 4.3 [2.4, 7.6] | 5.4 [3.6, 8.0] | 5.6 [3.9, 8.2] | 6.1 [4.4, 8.5] | 16.0 [13.4, 19.1] | 11.7 | 0.0000 |
| Nigeria 2018 | 5.8 [4.6, 7.4] | 10.4 [8.7, 12.3] | 15.3 [13.5, 17.3] | 21.0 [18.1, 24.4] | 25.1 [22.8, 27.5] | 19.3 | 0.0000 |
| Rwanda 2014-15 | 2.9 [2.0, 4.2] | 3.4 [2.3, 4.9] | 2.3 [1.5, 3.6] | 2.8 [1.9, 4.2] | 5.4 [4.2, 6.8] | 2.5 | 0.0032 |
| STP 2008-09 | 9.3 [6.0, 14.3] | 11.2 [7.5, 16.5] | 16.0 [10.4, 23.6] | 18.4 [13.3, 24.9] | 16.7 [11.3, 23.9] | 7.4 | 0.0887 |
| Senegal 2010-11 | 7.0 [4.9, 9.9] | 7.1 [5.4, 9.3] | 11.5 [9.0, 14.5] | 14.8 [11.5, 18.8] | 19.1 [14.9, 24.2] | 12.1 | 0.0000 |
| Sierra Leone 2013 | 5.9 [4.4, 7.8] | 6.2 [4.8, 8.1] | 8.0 [6.3, 10.2] | 9.8 [7.9, 12.1] | 11.6 [8.9, 15.0] | 5.7 | 0.0004 |
| South Africa 2016 | 16.2 [12.0, 21.5] | 22.4 [16.7, 29.3] | 20.3 [15.7, 26.0] | 23.2 [18.3, 29.0] | 22.4 [15.9, 30.5] | 6.2 | 0.3570 |
| Tanzania 2015-16 | 5.5 [4.0, 7.6] | 6.2 [5.0, 7.7] | 8.1 [6.6, 9.7] | 12.8 [10.9, 14.9] | 21.5 [19.6, 23.5] | 16.0 | 0.0000 |
| Togo 2013-14 | 4.6 [3.0, 6.9] | 7.3 [5.2, 10.0] | 12.6 [10.1, 15.6] | 18.4 [15.3, 21.9] | 23.9 [21.0, 26.9] | 19.3 | 0.0000 |
| Uganda 2016 | 3.2 [1.9, 5.1] | 3.5 [2.3, 5.4] | 5.3 [3.9, 7.3] | 5.8 [4.1, 8.3] | 11.5 [9.3, 14.1] | 8.3 | 0.0000 |
| Zimbabwe 2015 | 5.0 [3.6, 6.8] | 5.9 [4.5, 7.6] | 10.0 [8.0, 12.3] | 11.5 [9.6, 13.6] | 14.3 [12.5, 16.2] | 9.3 | 0.0000 |
| **EMRO** | **19.9 [4.2, 35.7]** | **21.8 [11.6, 31.9]** | **22.8 [15.7, 29.8]** | **25.2 [17.6, 32.8]** | **28.0 [19.0, 36.9]** | **8.1** | **-** |
| Egypt 2014 | 23.5 [20.6, 26.7] | 21.8 [18.8, 25.1] | 20.2 [17.7, 23.0] | 18.9 [16.4, 21.8] | 23.0 [20.1, 26.3] | -0.5 | 0.1463 |
| Jordan 2017-18 | 34.9 [31.4, 38.5] | 32.4 [28.4, 36.7] | 27.1 [23.4, 31.0] | 29.4 [25.1, 34.0] | 31.2 [27.3, 35.4] | -3.7 | 0.0764 |
| Yemen 2013 | 6.0 [4.2, 8.5] | 10.9 [8.6, 13.6] | 17.6 [14.7, 20.8] | 23.4 [20.4, 26.6] | 30.0 [26.1, 34.3] | 24.0 | 0.0000 |
| **EURO** | **12.8 [9.0, 16.6]** | **13.9 [10.2, 17.6]** | **13.5 [10.1, 16.9]** | **13.8 [9.6, 18.1]** | **11.8 [7.4, 16.3]** | **-1.0** | **-** |
| Albania 2017-18 | 11.5 [10.2, 13.0] | 13.6 [12.2, 15.2] | 13.3 [11.8, 15.0] | 13.2 [11.3, 15.4] | 11.5 [9.5, 13.9] | 0.0 | 0.2784 |
| Armenia 2015-16 | 4.8 [3.6, 6.4] | 6.3 [4.6, 8.5] | 6.3 [4.6, 8.6] | 6.3 [4.6, 8.5] | 4.7 [3.2, 7.1] | -0.1 | 0.4547 |
| Azerbaijan 2006 | 17.0 [14.9, 19.3] | 19.1 [16.7, 21.8] | 19.2 [16.9, 21.7] | 22.5 [18.6, 26.9] | 20.4 [17.5, 23.7] | 3.4 | 0.1973 |
| Kyrgyz Republic 2012 | 15.8 [12.9, 19.2] | 13.1 [11.0, 15.5] | 14.2 [11.8, 17.0] | 13.5 [11.4, 15.8] | 8.1 [6.3, 10.3] | -7.7 | 0.0000 |
| Moldova 2005 | 13.9 [11.5, 16.7] | 16.2 [13.6, 19.3] | 15.3 [13.2, 17.6] | 13.2 [11.8, 14.9] | 9.2 [7.8, 10.7] | -4.7 | 0.0001 |
| Tajikistan 2017 | 14.4 [12.2, 17.1] | 15.8 [13.6, 18.2] | 15.0 [12.7, 17.6] | 19.9 [17.0, 23.1] | 17.1 [15.2, 19.2] | 2.7 | 0.0156 |
| **PAHO** | **9.7 [7.2, 12.2]** | **11.9 [9.0, 14.8]** | **13.8 [10.7, 16.9]** | **15.5 [11.8, 19.1]** | **15.5 [11.3, 19.6]** | **5.8** | **-** |
| Bolivia 2008 | 16.3 [13.0, 20.3] | 19.6 [16.1, 23.6] | 22.8 [19.4, 26.7] | 19.4 [16.2, 23.1] | 14.5 [12.3, 16.9] | -1.8 | 0.0044 |
| Guatemala 2014-15 | 6.4 [5.5, 7.5] | 6.9 [5.9, 8.0] | 7.3 [6.4, 8.3] | 7.9 [7.0, 9.0] | 7.5 [6.5, 8.7] | 1.1 | 0.2658 |
| Guyana 2009 | 19.1 [14.9, 24.1] | 21.5 [17.7, 25.8] | 19.6 [16.4, 23.3] | 24.6 [20.8, 29.0] | 22.7 [18.9, 27.1] | 3.6 | 0.3033 |
| Haiti 2016-17 | 8.4 [6.6, 10.7] | 9.6 [7.8, 11.8] | 14.2 [12.1, 16.5] | 19.1 [16.7, 21.8] | 24.4 [22.0, 27.0] | 16.0 | 0.0000 |
| Honduras 2011-12 | 4.7 [4.0, 5.6] | 6.5 [5.5, 7.6] | 9.0 [7.7, 10.4] | 11.2 [10.0, 12.5] | 12.0 [10.5, 13.5] | 7.3 | 0.0000 |
| Peru 2012 | 9.1 [8.0, 10.4] | 9.5 [8.4, 10.8] | 9.2 [8.1, 10.4] | 9.5 [8.4, 10.7] | 9.6 [8.1, 11.2] | 0.5 | 0.9793 |
| **SEARO** | **7.7 [4.6, 10.9]** | **9.9 [5.4, 14.4]** | **11.4 [6.0, 16.7]** | **14.1 [9.0, 19.3]** | **17.0 [11.4, 22.6]** | **9.3** | **-** |
| India 2015-16 | 3.3 [3.2, 3.5] | 6.4 [6.2, 6.7] | 10.5 [10.2, 10.8] | 15.4 [15.0, 15.8] | 18.9 [18.4, 19.3] | 15.6 | 0.0000 |
| Maldives 2016-17 | 29.1 [26.5, 31.9] | 30.9 [28.4, 35.6] | 31.7 [28.8, 34.8] | 40.0 [34.3, 45.8] | 36.2 [30.5, 42.3] | 7.1 | 0.0051 |
| Myanmar 2015-16 | 4.6 [3.6, 6.0] | 7.8 [6.5, 9.3] | 10.5 [8.9, 12.2] | 13.5 [11.5, 15.7] | 15.3 [13.4, 17.5] | 10.7 | 0.0000 |
| Nepal 2016 | 3.2 [2.1, 4.9] | 5.3 [4.0, 6.9] | 6.0 [4.4, 8.1] | 8.6 [6.5, 11.2] | 15.2 [12.7, 18.0] | 12.0 | 0.0000 |
| Timor-Leste 2016 | 0.2 [0.0, 1.1] | 0.9 [0.3, 2.7] | 1.0 [0.4, 2.5] | 4.6 [2.6, 7.8] | 4.9 [3.2, 7.4] | 4.7 | 0.0000 |
| **WPRO** | **-** | **-** | **-** | **-** | **-** | **-** | **-** |
| Cambodia 2014 | 5.2 [4.0, 6.7] | 7.9 [6.3, 9.9] | 7.6 [6.2, 9.4] | 8.6 [7.1, 10.4] | 8.6 [7.4, 10.1] | 3.4 | 0.0120 |
| **Overall pooled prevalence** | **8.2 [7.1, 9.2]** | **10.1 [8.9, 11.2]** | **11.9 [10.5, 13.3]** | **14.2 [12.5, 15.8]** | **16.5 [14.7, 18.3]** | **8.3** | **-** |

AFRO, African region; DRC, Democratic Republic of the Congo; STP, Sao Tome and Principe; EMRO, Eastern Mediterranean region; EURO, European region; PAHO, Americas region; SEARO, Southeast Asian region; WPRO, Western Pacific region.

*Values are percentages and 95% CIs; estimates account for survey design. Regional estimates are pooled prevalences and 95% CIs, calculated with the available data from countries within that region. The WPRO region only had one country with available data (Cambodia), thus the regional prevalence was not calculated.

† Gaps are expressed in percentage points, calculated as the arithmetic difference between the prevalence in the highest vs. the prevalence in the lowest household wealth quintile (%Q5 - %Q1).

^‡^ p-values were obtained through tests for trend for each country. A p-value <0.05 indicate significant differences in the distribution of concurrent overweight/obesity and anaemia by household wealth quintiles.

**Supplemental Table 6**. Concurrent overweight/obesity and anaemia by education level among adult women (20-49 years old).

|  | **Education level^*^** | | | | | |
| --- | --- | --- | --- | --- | --- | --- |
| **Country** | **None (E1)** | **Primary (E2)** | **Secondary (E3)** | **Higher (E4)** | **Gap**^†^ | **p-value** |
| **AFRO** | **8.0 [6.4, 9.6]** | **11.3 [9.4, 13.1]** | **12.9 [10.7, 15.1]** | **14.0 [11.1, 16.9]** | **6.0** | **-** |
| Benin 2017-18 | 13.8 [12.5, 15.3] | 22.2 [19.2, 25.5] | 15.0 [12.7, 17.7] | 23.3 [16.3, 32.2] | 9.5 | 0.0000 |
| Burkina Faso 2010 | 4.0 [3.4, 4.7] | 7.8 [5.9, 10.2] | 12.8 [10.2, 15.9] | 1.2 [0.1, 9.0] | -2.8 | 0.0000 |
| Burundi 2016-17 | 1.3 [0.9, 1.8] | 2.2 [1.6, 3.0] | 4.2 [2.9, 5.9] | 2.5 [0.8, 7.8] | 1.2 | 0.0000 |
| Cameroon 2011 | 5.8 [4.3, 7.8] | 11.4 [10.0, 12.9] | 19.6 [17.6, 21.6] | 14.8 [11.1, 19.4] | 9.0 | 0.0000 |
| Congo 2011-12 | 10.4 [5.4, 19.0] | 12.3 [9.6, 15.6] | 17.1 [14.4, 20.2] | 24.5 [15.8, 36.0] | 14.1 | 0.0139 |
| Cote d'Ivoire 2011-12 | 12.4 [10.3, 14.9] | 15.8 [12.6, 19.5] | 14.5 [10.6, 19.6] | 18.8 [10.4, 31.5] | 6.4 | 0.2228 |
| DRC 2013-14 | 2.6 [1.5, 4.4] | 4.1 [3.2, 5.2] | 8.0 [6.6, 9.6] | 12.5 [8.6, 17.9] | 9.9 | 0.0000 |
| Eswatini 2006-07 | 12.8 [9.8, 16.5] | 16.5 [14.2, 19.1] | 16.6 [14.8, 18.6] | 17.7 [14.6, 21.3] | 4.9 | 0.3170 |
| Ethiopia 2016 | 1.0 [0.7, 1.4] | 2.1 [1.3, 3.3] | 3.5 [2.0, 6.2] | 3.2 [1.5, 6.6] | 2.2 | 0.0009 |
| Gabon 2012 | 25.6 [15.0, 40.2] | 32.8 [28.6, 37.3] | 30.1 [27.6, 32.8] | 26.9 [18.7, 37.1] | 1.3 | 0.5250 |
| Gambia 2013 | 13.7 [11.7, 16.0] | 13.3 [9.3, 18.6] | 12.9 [9.9, 16.7] | 17.3 [11.0, 26.1] | 3.6 | 0.7079 |
| Ghana 2014 | 11.3 [9.0, 14.0] | 16.2 [12.9, 20.1] | 19.1 [16.8, 21.7] | 21.2 [14.1, 30.6] | 9.9 | 0.0014 |
| Guinea 2018 | 12.1 [10.6, 13.7] | 14.3 [10.3, 19.5] | 16.6 [13.1, 20.8] | 10.0 [5.6, 17.1] | -2.1 | 0.1051 |
| Lesotho 2014 | 17.2 [4.1, 50.2] | 9.7 [7.8, 12.0] | 14.1 [11.5, 17.1] | 15.8 [11.4, 21.4] | -1.4 | 0.0702 |
| Madagascar 2008-09 | 1.7 [1.0, 2.9] | 1.6 [1.1, 2.3] | 3.8 [2.8, 5.0] | 2.2 [0.8, 5.9] | 0.5 | 0.0002 |
| Malawi 2015-16 | 7.4 [5.3, 10.1] | 5.7 [4.8, 6.8] | 8.3 [6.5, 10.6] | 10.5 [6.0, 17.8] | 3.1 | 0.0384 |
| Mali 2018 | 15.5 [13.8, 17.3] | 18.6 [14.6, 23.3] | 14.3 [11.1, 18.2] | 20.7 [11.5, 34.6] | 5.2 | 0.3109 |
| Mozambique 2011 | 5.1 [4.2, 6.1] | 8.7 [7.6, 9.9] | 14.8 [12.7, 17.1] | 16.5 [11.8, 22.6] | 11.4 | 0.0000 |
| Namibia 2013 | 5.1 [2.6, 10.0] | 6.4 [4.6, 8.9] | 6.8 [5.6, 8.3] | 8.5 [5.3, 13.3] | 3.4 | 0.6115 |
| Niger 2012 | 6.9 [5.7, 8.2] | 12.0 [8.5, 16.6] | 11.0 [7.4, 16.1] | 26.5 [15.3, 42.0] | 19.6 | 0.0001 |
| Nigeria 2018 | 10.0 [8.6, 11.5] | 18.9 [16.0, 22.1] | 19.1 [17.7, 20.7] | 23.9 [21.0, 27.1] | 13.9 | 0.0000 |
| Rwanda 2014-15 | 2.2 [1.4, 3.6] | 3.4 [2.8, 4.1] | 4.2 [3.1, 5.9] | 6.2 [3.6, 10.7] | 4.0 | 0.0286 |
| STP 2008-09^¶^ | 13.9 [7.8, 23.5] | 14.6 [11.2, 18.9] | 14.5 [10.8, 19.2] | - | - | - |
| Senegal 2010-11 | 11.3 [9.7, 13.1] | 16.1 [12.1, 21.1] | 14.0 [10.1, 19.0] | 14.1 [5.2, 32.9] | 2.8 | 0.1301 |
| Sierra Leone 2013 | 7.6 [6.5, 8.9] | 10.2 [7.7, 13.4] | 10.1 [7.5, 13.5] | 9.4 [5.1, 16.7] | 1.8 | 0.1746 |
| South Africa 2016 | 4.8 [1.9, 11.7] | 22.6 [15.8, 31.3] | 21.2 [18.1, 24.6] | 19.2 [13.1, 27.3] | 14.4 | 0.0908 |
| Tanzania 2015-16 | 9.0 [7.5, 10.9] | 11.1 [10.0, 12.3] | 16.4 [14.5, 18.6] | 27.3 [17.7, 39.7] | 18.3 | 0.0000 |
| Togo 2013-14 | 11.2 [9.3, 13.4] | 16.3 [13.9, 19.1] | 16.8 [14.3, 19.8] | 17.6 [10.7, 27.6] | 6.4 | 0.0050 |
| Uganda 2016 | 5.6 [3.5, 8.8] | 5.1 [4.1, 6.3] | 8.2 [6.1, 10.9] | 11.4 [7.7, 16.6] | 5.8 | 0.0013 |
| Zimbabwe 2015 | 2.3 [0.6, 8.3] | 8.4 [7.2, 9.9] | 10.1 [8.9, 11.4] | 14.3 [11.4, 17.9] | 12.0 | 0.0003 |
| **EMRO** | **21.2 [19.2, 23.2]** | **23.8 [21.3, 26.4]** | **25.1 [24.1, 26.2]** | **25.2 [23.6, 26.8]** | **4.0** | **-** |
| Egypt 2014 | 20.2 [17.7, 23.0] | 21.5 [17.8, 25.7] | 22.1 [20.3, 24.1] | 20.8 [17.6, 24.3] | 0.6 | 0.6499 |
| Jordan 2017-18 | 31.6 [22.4, 42.6] | 36.4 [29.5, 43.9] | 30.2 [27.7, 32.8] | 31.0 [28.0, 34.3] | -0.6 | 0.4519 |
| Yemen 2013^§^ | - | - | - | - | - | - |
| **EURO** | **14.1 [9.5, 18.8]** | **14.2 [13.4, 15.1]** | **16.8 [14.1, 19.5]** | **12.4 [7.3, 17.5]** | **-1.7** | **-** |
| Albania 2017-18 | 14.3 [7.4, 25.6] | 14.0 [12.8, 15.3] | 14.2 [12.8, 15.8] | 7.4 [5.9, 9.3] | -6.9 | 0.0000 |
| Armenia 2015-16^¶^ | - | 6.2 [3.4, 11.1] | 6.6 [5.4, 8.2] | 5.0 [3.9, 6.4] | - | - |
| Azerbaijan 2006 | 18.9 [16.5, 21.5] | 19.0 [13.3, 26.4] | 20.2 [18.6, 22.0] | 16.6 [14.0, 19.6] | -2.3 | 0.1706 |
| Kyrgyz Republic 2012^¶^ | - | - | 13.1 [11.6, 14.8] | 11.9 [10.4, 13.6] | - | - |
| Moldova 2005^¶^ | - | 10.5 [0.0, 97.1] | 14.6 [13.4, 15.8] | 8.9 [7.7, 10.3] | - | - |
| Tajikistan 2017 | 13.3 [8.6, 19.9] | 16.6 [12.7, 21.6] | 17.3 [15.9, 18.8] | 13.5 [11.6, 15.7] | 0.2 | 0.0096 |
| **PAHO** | **10.8 [8.3, 13.2]** | **13.9 [11.0, 16.9]** | **13.8 [10.3, 17.3]** | **11.1 [8.3, 13.8]** | **0.3** | **-** |
| Bolivia 2008 | 21.4 [14.8, 29.9] | 21.2 [19.0, 23.6] | 18.6 [15.8, 21.7] | 11.8 [9.6, 14.4] | -9.6 | 0.0000 |
| Guatemala 2014-15 | 7.2 [6.3, 8.3] | 8.1 [7.4, 8.8] | 6.6 [5.8, 7.4] | 5.3 [4.1, 6.7] | -1.9 | 0.0011 |
| Guyana 2009 | 17.6 [9.4, 30.4] | 22.9 [19.3, 27.4] | 21.5 [19.4, 23.7] | 21.3 [16.2, 27.5] | 3.7 | 0.8157 |
| Haiti 2016-17 | 12.5 [10.3, 15.1] | 16.8 [14.6, 19.1] | 18.0 [16.1, 20.0] | 16.5 [13.4, 20.1] | 4.0 | 0.0098 |
| Honduras 2011-12 | 8.6 [6.6, 11.0] | 9.0 [8.3, 9.9] | 9.0 [8.0, 10.2] | 9.6 [7.9, 11.7] | 1.0 | 0.9115 |
| Peru 2012 | 10.3 [7.9, 13.4] | 9.8 [8.1, 10.9] | 9.7 [8.8, 10.6] | 8.7 [7.7, 9.8] | -1.6 | 0.3496 |
| **SEARO** | **10.6 [6.8, 14.4]** | **13.5 [7.6, 19.4]** | **12.0 [6.9, 17.1]** | **12.4 [7.4, 17.4]** | **1.8** | **-** |
| India 2015-16 | 8.4 [8.2, 8.6] | 11.4 [11.0, 11.8] | 13.4 [13.1, 13.7] | 12.6 [12.2, 13.1] | 4.2 | 0.0000 |
| Maldives 2016-17 | 43.9 [35.9, 52.2] | 40.5 [37.7, 43.3] | 30.6 [27.8, 33.5] | 28.6 [24.1, 33.5] | -15.3 | 0.0000 |
| Myanmar 2015-16 | 8.7 [6.9, 10.9] | 10.9 [9.7, 12.2] | 10.4 [9.2, 11.8] | 12.9 [10.6, 15.6] | 4.2 | 0.0531 |
| Nepal 2016 | 5.8 [4.6, 7.2] | 8.0 [5.9, 10.8] | 10.1 [8.2, 12.5] | 10.5 [7.9, 14.0] | 4.7 | 0.0009 |
| Timor-Leste 2016 | 1.2 [0.5, 2.6] | 3.2 [1.7, 5.8] | 2.8 [1.9, 4.2] | 3.3 [1.7, 6.2] | 2.1 | 0.1314 |
| **WPRO** | **-** | **-** | **-** | **-** | **-** | **-** |
| Cambodia 2014 | 8.9 [7.2, 10.9] | 8.7 [7.6, 9.9] | 5.6 [4.7, 6.8] | 5.2 [3.4, 8.0] | -3.7 | 0.0003 |
| **Overall pooled prevalence** | **9.5 [8.3, 10.6]** | **12.6 [11.2, 14.0]** | **13.6 [12.2, 15.0]** | **13.4 [11.8, 14.9]** | **3.9** | **-** |

AFRO, African region; DRC, Democratic Republic of the Congo; STP, Sao Tome and Principe; EMRO, Eastern Mediterranean region; EURO, European region; PAHO, Americas region; SEARO, Southeast Asian region; WPRO, Western Pacific region.

*Values are percentages and 95% CIs; estimates account for survey design. Regional estimates are pooled prevalences and 95% CIs, calculated with the available data from countries within that region. The WPRO region only had one country with available data (Cambodia), thus the regional prevalence was not calculated.

† Gaps are expressed in percentage points, calculated as the arithmetic difference between the prevalence in the highest vs. the prevalence in the lowest education level (%E4 - %E1).

^‡^ p-values were obtained through tests for trend for each country. A p-value <0.05 indicate significant differences in the distribution of concurrent overweight/obesity and anaemia by education level.

^§^ Yemen has missing data on education level, and thus, the stratified estimates could not be calculated.

^¶^ Missing estimates for certain categories are missing for Sao Tome and Principe, Albania, Kyrgyz Republic and Moldova due to sample size <25.

**Supplemental Table 7.** Concurrent overweight/obesity and anaemia by area of residence among adult women (20-49 years old).

|  | **Area of residence^*^** | | | |
| --- | --- | --- | --- | --- |
| **Country** | **Urban** | **Rural** | **Gap**^†^ | **p-value**^‡^ |
| **AFRO** | **14.9 [12.6, 17.3]** | **8.7 [7.2, 10.1]** | **6.2** | **-** |
| Benin 2017-18 | 21.2 [19.3, 23.2] | 11.6 [10.3, 13.1] | 9.6 | 0.0000 |
| Burkina Faso 2010 | 11.5 [10.0, 13.2] | 3.0 [2.5, 3.7] | 8.5 | 0.0000 |
| Burundi 2016-17 | 4.1 [2.5, 6.6] | 1.9 [1.5, 2.3] | 2.2 | 0.0027 |
| Cameroon 2011 | 17.2 [15.7, 18.8] | 8.5 [7.3, 10.0] | 8.7 | 0.0000 |
| Congo 2011-12 | 19.6 [16.7, 22.9] | 8.2 [7.1, 9.5] | 11.4 | 0.0000 |
| Cote d'Ivoire 2011-12 | 19.0 [16.0, 22.3] | 8.9 [7.3, 10.8] | 10.1 | 0.0000 |
| DRC 2013-14 | 10.3 [8.7, 12.2] | 3.1 [2.3, 4.1] | 7.2 | 0.0000 |
| Eswatini 2006-07 | 20.3 [17.6, 23.2] | 14.8 [13.2, 16.6] | 5.5 | 0.0007 |
| Ethiopia 2016 | 3.9 [2.9, 5.2] | 1.0 [0.7, 1.4] | 2.9 | 0.0000 |
| Gabon 2012 | 31.2 [28.3, 34.1] | 22.7 [18.6, 27.4] | 8.5 | 0.0029 |
| Gambia 2013 | 15.3 [13.1, 17.8] | 11.6 [9.8, 13.6] | 3.7 | 0.0145 |
| Ghana 2014 | 20.5 [17.8, 23.5] | 12.6 [10.6, 15.0] | 7.9 | 0.0000 |
| Guinea 2018 | 17.1 [14.6, 19.9] | 10.3 [8.8, 11.9] | 6.8 | 0.0000 |
| Lesotho 2014 | 14.2 [11.1, 17.8] | 11.4 [9.8, 13.2] | 2.8 | 0.1307 |
| Madagascar 2008-09 | 4.5 [3.3, 6.0] | 1.7 [1.3, 2.3] | 2.8 | 0.0000 |
| Malawi 2015-16 | 11.5 [9.4, 13.9] | 5.7 [4.9, 6.6] | 5.8 | 0.0000 |
| Mali 2018 | 20.2 [16.9, 24.0] | 14.3 [12.7, 16.1] | 5.9 | 0.0018 |
| Mozambique 2011 | 13.4 [12.1, 14.9] | 5.7 [4.8, 6.8] | 7.7 | 0.0000 |
| Namibia 2013 | 8.3 [6.5, 10.1] | 4.9 [3.8, 6.3] | 3.4 | 0.0008 |
| Niger 2012 | 17.4 [14.7, 20.5] | 5.5 [4.4, 6.8] | 11.9 | 0.0000 |
| Nigeria 2018 | 20.9 [19.1, 22.7] | 12.8 [11.8, 13.9] | 8.1 | 0.0000 |
| Rwanda 2014-15 | 5.6 [4.5, 7.1] | 2.9 [2.4, 3.5] | 2.7 | 0.0000 |
| STP 2008-09 | 16.9 [12.9, 21.7] | 10.8 [8.5, 13.7] | 6.1 | 0.0128 |
| Senegal 2010-11 | 17.6 [14.7, 21.0] | 7.9 [6.6, 9.5] | 9.7 | 0.0000 |
| Sierra Leone 2013 | 11.5 [9.4, 14.1] | 6.8 [5.8, 8.0] | 4.7 | 0.0001 |
| South Africa 2016 | 20.8 [17.4, 24.6] | 20.6 [15.6, 26.6] | 0.2 | 0.9470 |
| Tanzania 2015-16 | 18.2 [16.4, 20.2] | 8.5 [7.5, 9.5] | 9.7 | 0.0000 |
| Togo 2013-14 | 20.9 [18.6, 23.4] | 9.3 [7.8, 10.9] | 11.6 | 0.0000 |
| Uganda 2016 | 9.0 [7.1, 11.4] | 5.5 [4.4, 6.7] | 3.5 | 0.0018 |
| Zimbabwe 2015 | 13.3 [11.7, 15.1] | 7.7 [6.6, 8.9] | 5.6 | 0.0000 |
| **EMRO** | **26.5 [18.9, 34.1]** | **23.4 [16.2, 30.6]** | **3.1** | **-** |
| Egypt 2014 | 21.9 [19.7, 24.3] | 21.1 [19.3, 22.9] | 0.8 | 0.5735 |
| Jordan 2017-18 | 30.6 [28.6, 32.7] | 33.3 [29.6, 37.2] | -2.7 | 0.2102 |
| Yemen 2013 | 26.1 [22.9, 29.6] | 14.3 [12.7, 16.0] | 11.8 | 0.0000 |
| **EURO** | **12.7 [8.6, 16.8]** | **13.3 [9.9, 16.8]** | **-0.6** | **-** |
| Albania 2017-18 | 13.1 [11.9, 14.4] | 12.0 [10.9, 13.1] | 1.1 | 0.1685 |
| Armenia 2015-16 | 5.9 [4.7, 7.5] | 5.3 [4.2, 6.8] | 0.6 | 0.5192 |
| Azerbaijan 2006 | 22.0 [19.7, 24.4] | 16.7 [15.1, 18.5] | 5.3 | 0.0004 |
| Kyrgyz Republic 2012 | 9.6 [7.9, 11.6] | 14.3 [13.0, 15.9] | -4.7 | 0.0002 |
| Moldova 2005 | 10.8 [9.7, 12.0] | 15.2 [13.8, 16.7] | -4.4 | 0.0000 |
| Tajikistan 2017 | 17.1 [14.4, 18.9] | 16.3 [14.7, 17.9] | 0.8 | 0.4937 |
| **PAHO** | **15.1 [11.9, 18.3]** | **12.2 [9.3, 15.1]** | **2.9** | **-** |
| Bolivia 2008 | 18.9 [17.1, 20.9] | 17.6 [15.1, 20.4] | 1.3 | 0.4470 |
| Guatemala 2014-15 | 8.0 [7.3, 8.8] | 6.6 [6.0, 7.2] | 1.4 | 0.0042 |
| Guyana 2009 | 24.1 [20.9, 27.5] | 20.7 [18.6, 23.1] | 3.4 | 0.0966 |
| Haiti 2016-17 | 20.5 [18.6, 22.6] | 13.0 [11.5, 14.5] | 7.5 | 0.0000 |
| Honduras 2011-12 | 10.5 [9.7, 11.4] | 7.4 [6.7, 8.1] | 3.1 | 0.0000 |
| Peru 2012 | 9.3 [8.6, 10.0] | 9.7 [8.8, 10.8] | -0.4 | 0.4676 |
| **SEARO** | **15.2 [9.9, 20.5]** | **10.8 [5.8, 15.8]** | **4.4** | **-** |
| India 2015-16 | 16.9 [16.5, 17.3] | 8.4 [8.3, 8.5] | 8.5 | 0.0000 |
| Maldives 2016-17 | 38.6 [34.3, 43.1] | 30.2 [28.7, 31.7] | 8.4 | 0.0002 |
| Myanmar 2015-16 | 14.8 [12.9, 16.9] | 9.0 [8.1, 10.0] | 5.8 | 0.0000 |
| Nepal 2016 | 9.2 [7.8, 10.8] | 6.0 [4.8, 7.6] | 3.2 | 0.0031 |
| Timor-Leste 2016 | 5.0 [3.5, 7.2] | 1.3 [0.8, 1.9] | 3.7 | 0.0000 |
| **WPRO** | **-** | **-** | **-** | **-** |
| Cambodia 2014 | 8.6 [7.4, 9.9] | 7.4 [6.6, 8.4] | 1.2 | 0.1435 |
| **Overall pooled prevalence** | **15.3 [13.8, 16.7]** | **10.7 [9.5, 11.8]** | **4.6** | **-** |

AFRO, African region; DRC, Democratic Republic of the Congo; STP, Sao Tome and Principe; EMRO, Eastern Mediterranean region; EURO, European region; PAHO, Americas region; SEARO, Southeast Asian region; WPRO, Western Pacific region.

*Values are percentages and 95% CIs; estimates account for survey design. Regional estimates are pooled prevalences and 95% CIs, calculated with the available data from countries within that region. The WPRO region only had one country with available data (Cambodia), thus the regional prevalence was not calculated.

† Gaps are expressed in percentage points, calculated as the arithmetic difference between the prevalence in urban vs. the prevalence in rural areas (%urban - %rural).

^‡^ p-values were obtained through chi-squared tests for each country. A p-value <0.05 indicate significant differences in the distribution of concurrent overweight/obesity and anaemia by area of residence.

**Supplemental Table 8.** Concurrent overweight/obesity and anaemia by household wealth quintiles among adolescent girls (15-19 years old).

|  | **Household wealth quintiles^*^** | | | | | | |
| --- | --- | --- | --- | --- | --- | --- | --- |
| **Country** | **Poorest (Q1)** | **Poorer (Q2)** | **Middle (Q3)** | **Richer (Q4)** | **Richest (Q5)** | **Gap**^†^ | **p-value**^‡^ |
| **AFRO** | **2.3 [1.7, 2.9]** | **3.1 [2.4, 3.8]** | **3.8 [3.0, 4.7]** | **5.6 [4.4, 6.8]** | **7.1 [5.7, 8.4]** | **4.8** | **-** |
| Benin 2017-18 | 2.3 [1.0, 5.6] | 2.6 [1.2, 5.7] | 4.3 [2.5, 7.3] | 5.1 [3.1, 8.2] | 8.6 [6.1, 12.1] | 6.3 | 0.0034 |
| Burkina Faso 2010 | 0.0 [0.0, 0.0] | 1.3 [0.3, 5.6] | 1.5 [0.5, 4.5] | 2.1 [1.0, 4.4] | 4.8 [2.9, 7.9] | 4.8 | 0.0052 |
| Burundi 2016-17 | 1.5 [0.6, 4.1] | 1.0 [0.3, 3.3] | 1.0 [0.3, 3.3] | 1.9 [0.8, 4.7] | 2.7 [1.3, 5.2] | 1.2 | 0.4692 |
| Cameroon 2011 | 1.3 [0.4, 4.1] | 3.8 [2.3, 6.5] | 8.8 [5.4, 14.0] | 8.2 [5.7, 11.5] | 11.0 [8.4, 14.5] | 9.7 | 0.0001 |
| Congo 2011-12 | 2.4 [1.2, 4.8] | 1.9 [0.9, 4.0] | 1.5 [0.4, 5.4] | 5.8 [2.3, 13.9] | 3.4 [1.2, 9.6] | 1.0 | 0.1579 |
| Cote d'Ivoire 2011-12 | 3.7 [0.9, 13.7] | 2.7 [0.7, 9.7] | 9.0 [4.5, 17.1] | 13.8 [8.2, 22.1] | 10.8 [6.7, 17.0] | 7.1 | 0.0561 |
| DRC 2013-14 | 0.3 [0.1, 1.5] | 2.0 [0.8, 5.1] | 2.1 [1.1, 3.9] | 2.9 [1.3, 6.4] | 5.7 [3.6, 9.0] | 5.4 | 0.0030 |
| Eswatini 2006-07 | 5.5 [3.7, 8.3] | 6.6 [4.8, 9.1] | 7.8 [5.5, 11.0] | 8.7 [5.8, 12.9] | 11.6 [8.6, 15.5] | 6.1 | 0.2410 |
| Ethiopia 2016 | 0.3 [0.1, 0.8] | 0.6 [0.2, 2.4] | 1.1 [0.3, 3.5] | 0.1 [0.0, 0.7] | 2.2 [1.2, 4.0] | 1.9 | 0.0047 |
| Gabon 2012 | 10.6 [6.4, 17.0] | 9.3 [3.9, 20.6] | 15.2 [7.4, 28.5] | 7.0 [3.7, 12.9] | 12.5 [6.0, 24.3] | 1.9 | 0.5327 |
| Gambia 2013 | 5.0 [2.7, 9.0] | 4.5 [2.4, 8.2] | 6.4 [3.3, 12.1] | 4.7 [2.2, 9.6] | 9.6 [4.8, 18.1] | 4.6 | 0.2924 |
| Ghana 2014 | 1.6 [0.7, 3.7] | 0.8 [0.2, 3.4] | 6.0 [2.6, 13.3] | 6.7 [3.4, 12.7] | 13.2 [7.9, 21.2] | 11.6 | 0.0000 |
| Guinea 2018 | 3.3 [1.5, 7.0] | 4.5 [2.2, 8.9] | 6.5 [4.1, 11.2] | 9.3 [6.3, 13.7] | 10.0 [6.4, 15.4] | 6.7 | 0.0407 |
| Lesotho 2014 | 2.8 [1.1, 6.5] | 4.8 [2.1, 10.5] | 6.9 [2.9, 15.8] | 12.6 [7.1, 21.3] | 5.1 [2.5, 10.3] | 2.3 | 0.0514 |
| Madagascar 2008-09 | 0.7 [0.2, 2.8] | 0.0 [0.0, 0.0] | 0.4 [0.1, 2.6] | 0.5 [0.2, 1.8] | 0.8 [0.3, 2.0] | 0.1 | 0.5905 |
| Malawi 2015-16 | 0.2 [0.0, 1.2] | 1.5 [0.6, 4.5] | 1.7 [0.6, 4.9] | 3.2 [1.6, 6.2] | 7.6 [4.7, 12.2] | 7.4 | 0.0000 |
| Mali 2018 | 3.9 [1.2, 12.0] | 6.4 [2.3, 16.4] | 5.2 [2.2, 11.9] | 7.3 [4.3, 12.2] | 8.0 [4.7, 13.4] | 4.1 | 0.7764 |
| Mozambique 2011 | 3.3 [1.1, 9.0] | 2.7 [1.4, 5.1] | 2.7 [1.4, 5.2] | 4.4 [3.0, 6.5] | 10.6 [8.5, 13.0] | 7.3 | 0.0001 |
| Namibia 2013 | 0.8 [0.1, 6.1] | 0.4 [0.1, 3.1] | 0.7 [0.1, 4.7] | 2.0 [0.7, 5.6] | 3.6 [1.0, 12.1] | 2.8 | 0.1786 |
| Niger 2012 | 0.7 [0.1, 5.4] | 1.4 [0.3, 5.7] | 0.7 [0.1, 5.1] | 1.0 [0.2, 5.3] | 3.4 [1.8, 6.5] | 2.7 | 0.2146 |
| Nigeria 2018 | 1.0 [0.5, 1.8] | 4.6 [3.0, 6.9] | 4.8 [2.8, 8.0] | 10.7 [6.4, 17.2] | 9.2 [6.4, 13.0] | 8.2 | 0.0003 |
| Rwanda 2014-15 | 3.3 [1.5, 7.1] | 1.8 [0.7, 4.9] | 2.6 [1.0, 6.5] | 2.7 [1.3, 5.7] | 3.9 [2.6, 5.8] | 0.6 | 0.6977 |
| STP 2008-09 | 7.6 [3.8, 14.5] | 8.4 [3.9, 17.2] | 16.2 [8.4, 28.9] | 6.1 [2.2, 15.9] | 4.5 [1.5, 12.6] | -3.1 | 0.1532 |
| Senegal 2010-11 | 2.9 [1.3, 6.2] | 3.4 [1.7, 6.4] | 4.0 [1.9, 8.0] | 6.5 [2.9, 14.0] | 7.4 [3.0, 16.8] | 4.5 | 0.3874 |
| Sierra Leone 2013 | 5.5 [2.7, 11.0] | 3.7 [1.7, 7.8] | 2.1 [0.9, 4.9] | 6.2 [3.8, 10.2] | 6.2 [4.0, 9.5] | 0.7 | 0.2558 |
| South Africa 2016 | 9.6 [5.4, 16.4] | 11.8 [7.0, 19.3] | 11.0 [5.4, 21.3] | 9.2 [4.8, 17.1] | 12.2 [3.3, 36.3] | 2.6 | 0.9691 |
| Tanzania 2015-16 | 3.8 [2.0, 7.2] | 4.4 [2.7, 7.1] | 6.9 [4.7, 10.0] | 5.1 [3.3, 7.8] | 9.6 [6.4, 14.1] | 5.8 | 0.0132 |
| Togo 2013-14 | 1.3 [0.3, 5.3] | 2.4 [0.7, 7.9] | 7.1 [3.7, 13.2] | 8.1 [4.6, 13.8] | 16.6 [11.5, 23.3] | 15.3 | 0.0000 |
| Uganda 2016 | 0.7 [0.2, 2.8] | 2.6 [0.9, 7.3] | 2.5 [1.0, 6.3] | 4.9 [2.6, 9.2] | 7.3 [4.1, 12.6] | 6.6 | 0.0108 |
| Zimbabwe 2015 | 2.3 [1.1, 4.9] | 3.6 [1.4, 8.8] | 3.1 [1.8, 5.3] | 6.7 [3.8, 11.7] | 5.5 [3.6, 8.4] | 3.2 | 0.1343 |
| **EMRO** | **-** | **-** | **-** | **-** | **-** | **-** | **-** |
| Egypt 2014^¶^ | 17.7 [5.0, 46.6] | 11.4 [3.7, 30.1] | 7.2 [2.2, 21.3] | 12.0 [12.0, 12.0] | - | - | - |
| Jordan 2017-18^¶^ | 26.2 [15.7, 40.3] | 13.9 [4.4, 36.3] | - | - | - | - | - |
| Yemen 2013 | 0.2 [0.0, 1.2] | 1.6 [0.7, 4.0] | 4.1 [2.4, 6.9] | 9.4 [5.6, 15.2] | 10.8 [7.0, 16.3] | 10.6 | 0.0000 |
| **EURO** | **2.8 [1.8, 3.8]** | **2.9 [2.0, 3.7]** | **3.1 [2.2, 3.9]** | **2.3 [1.5, 3.0]** | **2.4 [0.7, 4.1]** | **-0.4** | **-** |
| Albania 2017-18 | 1.4 [0.7, 2.8] | 2.0 [0.8, 4.5] | 2.6 [1.3, 5.0] | 2.8 [1.0, 7.5] | 10.1 [4.9, 19.5] | 8.7 | 0.0001 |
| Armenia 2015-16 | 1.8 [0.5, 7.0] | 0.0 [0.0, 0.0] | 0.0 [0.0, 0.0] | 1.3 [0.6, 2.9] | 0.0 [0.0, 0.0] | -1.8 | 0.2750 |
| Azerbaijan 2006 | 3.3 [2.0, 5.4] | 5.0 [3.7, 6.8] | 4.6 [2.1, 9.6] | 3.3 [0.9, 11.3] | 1.8 [0.6, 4.7] | -1.5 | 0.5369 |
| Kyrgyz Republic 2012 | 4.9 [2.8, 8.5] | 2.2 [1.1, 4.3] | 2.5 [1.1, 5.4] | 1.9 [0.7, 5.0] | 0.4 [0.1, 3.1] | -4.5 | 0.0179 |
| Moldova 2005 | 3.9 [2.5, 6.0] | 2.0 [1.0, 4.0] | 2.4 [1.3, 4.1] | 3.0 [1.7, 5.1] | 2.0 [1.2, 3.2] | -1.9 | 0.5875 |
| Tajikistan 2017 | 3.5 [1.6, 7.8] | 3.1 [1.5, 6.4] | 3.1 [1.7, 5.6] | 2.6 [1.3, 5.2] | 4.2 [2.4, 7.1] | 0.7 | 0.8784 |
| **PAHO** | **4.6 [2.9, 6.3]** | **4.7 [3.0, 6.4]** | **3.7 [2.6, 4.8]** | **5.7 [3.7, 7.7]** | **4.3 [3.0, 5.7]** | **-0.3** | **-** |
| Bolivia 2008 | 8.5 [4.6, 15.1] | 15.3 [10.1, 22.5] | 8.9 [5.1, 15.2] | 12.8 [8.5, 19.0] | 6.0 [3.2, 11.0] | -2.5 | 0.0644 |
| Guatemala 2014-15 | 4.0 [2.8, 5.5] | 2.1 [1.3, 3.2] | 1.8 [1.2, 2.9] | 3.6 [2.5, 5.1] | 2.6 [1.7, 4.1] | -1.4 | 0.0319 |
| Guyana 2009 | 11.9 [6.9, 19.7] | 6.2 [3.1, 11.7] | 2.2 [0.7, 7.1] | 12.1 [7.4, 19.3] | 5.9 [2.8, 11.9] | -6.0 | 0.0142 |
| Haiti 2016-17 | 3.6 [1.9, 6.5] | 5.5 [3.4, 8.7] | 5.4 [3.3, 8.7] | 8.2 [5.7, 11.8] | 6.4 [4.3, 9.6] | 2.8 | 0.1866 |
| Honduras 2011-12 | 1.4 [0.8, 2.3] | 2.5 [1.6, 3.8] | 3.1 [2.1, 4.5] | 2.7 [1.7, 4.4] | 2.8 [1.8, 4.5] | 1.4 | 0.2989 |
| Peru 2012 | 4.9 [3.5, 6.9] | 4.7 [3.4, 6.5] | 2.9 [1.6, 5.1] | 2.5 [1.4, 4.5] | 4.3 [2.5, 7.1] | -0.6 | 0.2243 |
| **SEARO** | **2.0 [0.6, 3.4]** | **2.7 [1.0, 4.4]** | **2.9 [1.3, 4.5]** | **3.0 [2.2, 3.9]** | **3.1 [1.5, 4.7]** | **1.1** | **-** |
| India 2015-16 | 0.9 [0.7, 1.1] | 1.4 [1.2, 1.6] | 2.1 [1.9, 2.5] | 3.4 [3.0, 3.8] | 4.9 [4.3, 5.8] | 4.0 | 0.0000 |
| Maldives 2016-17 | 10.3 [7.1, 14.7] | 7.3 [4.7, 11.2] | 8.9 [5.1, 15.1] | 7.2 [2.8, 17.4] | 17.5 [8.8, 31.8] | 7.2 | 0.1466 |
| Myanmar 2015-16 | 2.1 [0.9, 4.9] | 1.4 [0.4, 4.5] | 1.9 [0.7, 4.6] | 2.4 [0.9, 6.3] | 2.9 [1.2, 6.5] | 0.8 | 0.8301 |
| Nepal 2016 | 0.5 [0.1, 2.1] | 2.4 [1.1, 5.4] | 1.4 [0.5, 4.2] | 3.7 [1.6, 8.3] | 1.4 [0.5, 4.2] | 0.9 | 0.1161 |
| Timor-Leste 2016 | 1.2 [0.2, 8.6] | 0.0 [0.0, 0.0] | 0.0 [0.0, 0.0] | 0.0 [0.0, 0.0] | 1.6 [0.7, 3.6] | 0.4 | 0.2725 |
| **WPRO** | **-** | **-** | **-** | **-** | **-** | **-** | **-** |
| Cambodia 2014 | 0.5 [0.1, 2.0] | 1.0 [0.4, 2.5] | 1.5 [0.4, 4.9] | 2.2 [0.9, 5.2] | 2.4 [1.2, 4.6] | 1.9 | 0.3545 |
| **Overall pooled prevalence** | **2.4 [2.0, 2.9]** | **3.1 [2.6, 3.6]** | **3.4 [2.9, 3.8]** | **4.7 [4.0, 5.3]** | **5.7 [4.9, 6.5]** | **3.3** | **-** |

AFRO, African region; DRC, Democratic Republic of the Congo; STP, Sao Tome and Principe; EMRO, Eastern Mediterranean region; EURO, European region; PAHO, Americas region; SEARO, Southeast Asian region; WPRO, Western Pacific region.

*Values are percentages and 95% CIs; estimates account for survey design. Regional estimates are pooled prevalences and 95% CIs, calculated with the available data from countries within that region. The WPRO region only had one country with available data (Cambodia), thus the regional prevalence was not calculated.

† Gaps are expressed in percentage points, calculated as the arithmetic difference between the prevalence in the highest vs. the prevalence in the lowest household wealth quintile (%Q5 - %Q1).

^‡^ p-values were obtained through tests for trend for each country. A p-value <0.05 indicate significant differences in the distribution of concurrent overweight/obesity and anaemia by household wealth quintiles.

^¶^ Missing estimates for certain categories are missing for Egypt and Jordan due to sample size <25, and thus, the pooled prevalence is missing for the Eastern and Mediterranean region.

**Supplemental Table 9**. Concurrent overweight/obesity and anaemia by education level among adolescent girls (15-19 years old).

|  | **Education level^*^** | | | | |
| --- | --- | --- | --- | --- | --- |
| **Country** | **None (E1)** | **Primary (E2)** | **Secondary or higher (E3)** | **Gap**^†^ | **p-value**^‡^ |
| **AFRO** | **3.1 [2.2, 4.0]** | **3.9 [3.0, 4.8]** | **5.3 [4.1, 6.6]** | **2.2** | **-** |
| Benin 2017-18 | 3.6 [2.2, 5.8] | 4.2 [2.5, 6.8] | 6.2 [4.6, 8.3] | 2.6 | 0.1126 |
| Burkina Faso 2010 | 1.3 [0.6, 2.6] | 1.8 [0.6, 5.0] | 5.6 [3.1, 10.1] | 4.3 | 0.0027 |
| Burundi 2016-17 | 3.3 [0.7, 14.5] | 1.2 [0.6, 2.3] | 1.9 [1.1, 3.3] | -1.4 | 0.3740 |
| Cameroon 2011 | 3.0 [0.4, 18.3] | 6.8 [4.6, 9.9] | 8.0 [6.4, 9.9] | 5.0 | 0.3686 |
| Congo 2011-12 | 1.6 [1.3, 2.0] | 4.7 [2.0, 10.6] | 2.6 [1.3, 5.1] | 1.0 | 0.3195 |
| Cote d'Ivoire 2011-12 | 11.4 [7.6, 16.9] | 9.9 [5.9, 16.2] | 5.8 [3.1, 10.8] | -5.6 | 0.1594 |
| DRC 2013-14 | 1.5 [0.3, 7.4] | 2.8 [1.5, 5.1] | 3.3 [2.0, 5.3] | 1.8 | 0.6472 |
| Eswatini 2006-07^¶^ | - | 6.9 [5.1, 9.2] | 8.8 [6.7, 11.6] | - | - |
| Ethiopia 2016 | 2.0 [0.8, 4.9] | 0.8 [0.4, 1.6] | 1.0 [0.5, 2.2] | -1.0 | 0.2219 |
| Gabon 2012^¶^ | - | 7.2 [4.2, 12.1] | 11.7 [8.3, 16.3] | - | - |
| Gambia 2013 | 5.1 [2.7, 9.7] | 7.4 [3.9, 13.5] | 6.2 [4.0, 9.4] | 1.1 | 0.7235 |
| Ghana 2014 | 0.0 [0.0, 0.0] | 3.9 [1.6, 9.0] | 6.2 [4.3, 8.8] | 6.2 | 0.2761 |
| Guinea 2018 | 5.1 [3.6, 7.3] | 9.6 [5.9, 15.2] | 8.8 [6.0, 12.8] | 3.7 | 0.0760 |
| Lesotho 2014^¶^ | - | 4.2 [2.1, 8.2] | 8.0 [5.5, 11.4] | - | - |
| Madagascar 2008-09 | 0.3 [0.0, 2.1] | 0.7 [0.3, 1.5] | 0.4 [0.1, 1.2] | 0.1 | 0.6094 |
| Malawi 2015-16 | 0.0 [0.0, 0.0] | 2.5 [1.5, 4.1] | 5.3 [3.2, 8.9] | 5.3 | 0.0636 |
| Mali 2018 | 7.6 [4.8, 12.0] | 3.0 [1.1, 8.0] | 6.9 [4.2, 11.0] | -0.7 | 0.2276 |
| Mozambique 2011 | 3.3 [1.7, 6.2] | 4.4 [3.3, 5.9] | 7.7 [6.0, 9.8] | 4.4 | 0.0034 |
| Namibia 2013^¶^ | - | 1.1 [0.3, 4.7] | 1.8 [0.8, 4.2] | - | - |
| Niger 2012 | 1.2 [0.6, 2.7] | 2.9 [1.2, 7.0] | 1.4 [0.5, 3.9] | 0.2 | 0.2608 |
| Nigeria 2018 | 2.4 [1.4, 4.0] | 3.3 [1.7, 6.5] | 8.2 [6.4, 10.4] | 5.8 | 0.0000 |
| Rwanda 2014-15^¶^ | - | 3.5 [2.4, 5.1] | 2.3 [1.4, 3.7] | - | - |
| STP 2008-09^¶^ | - | 5.5 [3.1, 9.5] | 8.4 [5.0, 13.7] | - | - |
| Senegal 2010-11 | 5.0 [2.8, 9.0] | 3.0 [1.2, 7.5] | 6.1 [3.3, 10.9] | 1.1 | 0.4595 |
| Sierra Leone 2013 | 3.0 [1.2, 7.8] | 7.3 [4.3, 12.3] | 5.0 [3.6, 6.8] | 2.0 | 0.2093 |
| South Africa 2016^¶^ | - | 8.8 [2.2, 29.6] | 10.8 [7.9, 14.5] | - | - |
| Tanzania 2015-16 | 3.4 [1.3, 8.9] | 6.3 [4.9, 8.0] | 7.3 [5.4, 10.0] | 3.9 | 0.2534 |
| Togo 2013-14 | 1.4 [0.2, 9.8] | 10.1 [6.6, 15.2] | 8.2 [5.7, 11.6] | 6.8 | 0.0679 |
| Uganda 2016 | 0.0 [0.0, 0.0] | 2.0 [1.1, 3.5] | 7.3 [4.8, 11.0] | 7.3 | 0.0003 |
| Zimbabwe 2015^¶^ | - | 1.3 [0.4, 4.2] | 5.2 [3.9, 6.9] | - | - |
| **EMRO** | **-** | **-** | **-** | **-** | **-** |
| Egypt 2014^¶^ | - | - | 15.2 [7.8, 27.6] | - | - |
| Jordan 2017-18^¶^ | - | - | 22.3 [12.7, 36.3] | - | - |
| Yemen 2013^§^ | - | - | - | - | - |
| **EURO** | **-** | **-** | **-** | **-** | **-** |
| Albania 2017-18^¶^ | - | 2.6 [1.4, 4.7] | 3.9 [2.3, 6.6] | - | - |
| Armenia 2015-16^¶^ | - | 1.4 [0.3, 5.7] | 0.5 [0.1, 1.9] | - | - |
| Azerbaijan 2006 | 4.7 [3.5, 6.1] | 10.5 [10.2, 10.9] | 3.4 [2.1, 5.3] | -1.3 | 0.1679 |
| Kyrgyz Republic 2012^¶^ | - | - | 2.3 [1.6, 3.2] | - | - |
| Moldova 2005^¶^ | - | - | 2.7 [1.9, 3.7] | - | - |
| Tajikistan 2017^¶^ | - | 0.0 [0.0, 0.0] | 3.4 [2.5, 4.7] | - | - |
| **PAHO** | **4.4 [0.9, 7.9]** | **2.8 [2.2, 3.4]** | **4.0 [2.5, 5.5]** | **-0.4** | **-** |
| Bolivia 2008^¶^ | - | 12.5 [8.5, 18.0] | 9.5 [7.2, 12.5] | - | - |
| Guatemala 2014-15 | 5.1 [2.5, 10.2] | 2.5 [1.9, 3.3] | 2.9 [2.2, 3.7] | -2.2 | 0.2156 |
| Guyana 2009^¶^ | - | 8.7 [2.4, 27.1] | 7.5 [5.6, 10.0] | - | - |
| Haiti 2016-17 | 12.0 [2.8, 38.9] | 3.6 [2.3, 5.5] | 7.0 [5.5, 8.9] | -5.0 | 0.0191 |
| Honduras 2011-12 | 1.5 [1.4, 1.7] | 2.2 [1.6, 3.1] | 2.8 [2.1, 3.6] | 1.3 | 0.3914 |
| Peru 2012^¶^ | - | 5.6 [3.5, 8.8] | 3.6 [2.9, 4.5] | - | - |
| **SEARO** | **1.3 [0.9, 1.7]** | **1.6 [1.3, 1.9]** | **1.7 [0.9, 2.6]** | **0.4** | **-** |
| India 2015-16 | 1.3 [1.0, 1.7] | 1.7 [1.4, 2.1] | 2.5 [2.4, 2.7] | 1.2 | 0.0000 |
| Maldives 2016-17^¶^ | - | - | 10.3 [7.6, 13.7] | - | - |
| Myanmar 2015-16 | 2.3 [0.7, 6.9] | 2.0 [0.6, 6.5] | 2.2 [1.3, 3.6] | -0.1 | 0.9645 |
| Nepal 2016 | 3.7 [1.0, 12.2] | 1.5 [0.5, 4.8] | 2.0 [1.2, 3.3] | -1.7 | 0.5764 |
| Timor-Leste 2016 | 2.6 [0.3, 17.4] | 0.0 [0.0, 0.0] | 0.4 [0.2, 1.0] | -2.2 | 0.0995 |
| **WPRO** | **-** | **-** | **-** | - | **-** |
| Cambodia 2014 | 4.5 [0.5, 30.4] | 1.7 [0.7, 4.1] | 1.5 [0.9, 2.5] | -3.0 | 0.4907 |
| **Overall pooled prevalence** | **2.9 [2.2, 3.6]** | **3.3 [2.7, 3.9]** | **4.3 [3.7, 5.0]** | **1.4** | **-** |

AFRO, African region; DRC, Democratic Republic of the Congo; STP, Sao Tome and Principe; EMRO, Eastern Mediterranean region; EURO, European region; PAHO, Americas region; SEARO, Southeast Asian region; WPRO, Western Pacific region.

*Values are percentages and 95% CIs; estimates account for survey design. Regional estimates are pooled prevalences and 95% CIs, calculated with the available data from countries within that region. The WPRO region only had one country with available data (Cambodia), thus the regional prevalence was not calculated.

† Gaps are expressed in percentage points, calculated as the arithmetic difference between the prevalence in the highest vs. the prevalence in the lowest education level (%E3 - %E1).

^‡^ p-values were obtained through tests for trend for each country. A p-value <0.05 indicate significant differences in the distribution of concurrent overweight/obesity and anaemia by education level.

^§^ Yemen has missing data on education level, and thus, the stratified estimates could not be calculated.

^¶^ Missing estimates for certain categories are missing for Eswatini, Gabon, Lesotho, Namibia, Rwanda, Sao Tome and Principe, South Africa, Zimbabwe, Egypt, Jordan, Albania, Armenia, Kyrgyz Republic, Moldova, Tajikistan, Bolivia, Guyana, Peru and Maldives due to sample size <25. These countries were excluded for the calculation of pooled estimates and, as a result, the pooled prevalence for the Eastern Mediterranean and the European region could not be calculated.

**Supplemental Table 10**. Concurrent overweight/obesity and anaemia by area of residence among adolescent girls (15-19 years old).

|  | **Area of residence^*^** | | | |
| --- | --- | --- | --- | --- |
| **Country** | **Urban** | **Rural** | **Gap**^†^ | **p-value**^‡^ |
| **AFRO** | **6.7 [5.5, 7.9]** | **3.7 [3.0, 4.4]** | **3.0** | **-** |
| Benin 2017-18 | 7.7 [5.8, 10.2] | 3.0 [2.0, 4.3] | 4.7 | 0.0001 |
| Burkina Faso 2010 | 5.0 [3.1, 7.9] | 1.0 [0.5, 2.1] | 4.0 | 0.0001 |
| Burundi 2016-17 | 3.1 [1.3, 7.2] | 1.5 [0.9, 2.4] | 1.6 | 0.1299 |
| Cameroon 2011 | 10.0 [8.0, 12.5] | 3.8 [2.5, 5.5] | 6.2 | 0.0000 |
| Congo 2011-12 | 3.0 [1.5, 6.2] | 2.8 [1.7, 4.5] | 0.2 | 0.8531 |
| Cote d'Ivoire 2011-12 | 11.2 [7.9, 15.8] | 5.3 [2.9, 9.6] | 5.9 | 0.0283 |
| DRC 2013-14 | 4.9 [3.1, 7.6] | 1.7 [0.9, 3.0] | 3.2 | 0.0034 |
| Eswatini 2006-07 | 7.6 [4.8, 11.8] | 8.3 [6.4, 10.8] | -0.7 | 0.7128 |
| Ethiopia 2016 | 2.8 [1.5, 5.0] | 0.5 [0.2, 1.0] | 2.3 | 0.0001 |
| Gabon 2012 | 10.9 [7.9, 14.9] | 11.0 [7.1, 16.8] | -0.1 | 0.9777 |
| Gambia 2013 | 7.1 [4.4, 11.4] | 5.1 [3.4, 7.4] | 2.0 | 0.2854 |
| Ghana 2014 | 8.0 [5.4, 11.6] | 2.7 [1.4, 5.3] | 5.3 | 0.0037 |
| Guinea 2018 | 9.1 [6.5, 12.5] | 5.9 [4.2, 8.1] | 3.2 | 0.0657 |
| Lesotho 2014 | 9.1 [5.0, 15.9] | 5.9 [3.8, 9.9] | 3.2 | 0.2390 |
| Madagascar 2008-09 | 1.1 [0.4, 2.8] | 0.4 [0.2, 0.9] | 0.7 | 0.0973 |
| Malawi 2015-16 | 8.5 [5.0, 14.1] | 2.1 [1.3, 3.3] | 6.4 | 0.0000 |
| Mali 2018 | 8.6 [5.6, 13.0] | 5.5 [3.5, 8.5] | 3.1 | 0.1467 |
| Mozambique 2011 | 7.5 [6.2, 9.2] | 3.9 [2.8, 5.6] | 3.6 | 0.0012 |
| Namibia 2013 | 2.2 [0.8, 6.4] | 1.1 [0.4, 2.8] | 1.1 | 0.3228 |
| Niger 2012 | 3.8 [2.0, 7.1] | 0.9 [0.4, 2.4] | 2.9 | 0.0093 |
| Nigeria 2018 | 8.5 [6.3, 11.5] | 4.5 [3.3, 5.9] | 4.0 | 0.0018 |
| Rwanda 2014-15 | 3.8 [2.3, 6.2] | 2.7 [1.8, 4.0] | 1.1 | 0.2836 |
| STP 2008-09 | 5.5 [3.2, 9.4] | 11.9 [7.2, 19.1] | -6.4 | 0.0323 |
| Senegal 2010-11 | 7.4 [4.5, 12.1] | 2.6 [1.6, 4.2] | 4.8 | 0.0023 |
| Sierra Leone 2013 | 6.2 [4.3, 8.8] | 4.2 [2.7, 6.3] | 2.0 | 0.1648 |
| South Africa 2016 | 9.6 [5.8, 15.3] | 11.9 [8.4, 16.5] | -2.3 | 0.4686 |
| Tanzania 2015-16 | 9.6 [6.7, 13.5] | 4.6 [3.6, 5.7] | 5.0 | 0.0004 |
| Togo 2013-14 | 12.3 [8.7, 17.0] | 4.4 [2.7, 7.2] | 7.9 | 0.0004 |
| Uganda 2016 | 6.0 [3.3, 10.6] | 3.2 [2.1, 4.9] | 2.8 | 0.0889 |
| Zimbabwe 2015 | 6.8 [4.6, 10.0] | 3.1 [2.1, 4.6] | 3.7 | 0.0046 |
| **EMRO** | **12.6 [9.6, 15.5]** | **3.5 [2.5, 4.5]** | **9.1** | **-** |
| Egypt 2014 | 10.6 [2.1, 38.9] | 13.6 [7.4, 23.6] | -3.0 | 0.7403 |
| Jordan 2017-18^¶^ | 23.8 [14.1, 37.3] | - | - | - |
| Yemen 2013 | 11.5 [7.9, 16.3] | 2.5 [1.7, 3.7] | 9.0 | 0.0000 |
| **EURO** | **2.1 [1.0, 3.2]** | **2.7 [1.8, 3.6]** | **-0.6** | **-** |
| Albania 2017-18 | 4.6 [2.6, 8.1] | 2.1 [1.3, 3.6] | 2.5 | 0.0456 |
| Armenia 2015-16 | 0.2 [0.0, 1.3] | 1.1 [0.4, 3.5] | -0.9 | 0.0670 |
| Azerbaijan 2006 | 3.0 [1.5, 5.9] | 4.3 [2.8, 6.6] | -1.3 | 0.3591 |
| Kyrgyz Republic 2012 | 0.9 [0.3, 2.4] | 3.1 [2.1, 4.5] | -2.2 | 0.0165 |
| Moldova 2005 | 2.3 [1.5, 3.7] | 2.9 [1.8, 4.5] | -0.6 | 0.5342 |
| Tajikistan 2017 | 3.9 [2.3, 6.4] | 3.2 [2.1, 4.7] | 0.7 | 0.5387 |
| **PAHO** | **5.2 [3.7, 6.7]** | **4.9 [3.5, 6.4]** | **0.3** | **-** |
| Bolivia 2008 | 9.5 [7.1, 12.6] | 11.9 [8.4, 16.7] | -2.4 | 0.3114 |
| Guatemala 2014-15 | 2.6 [1.9, 3.5] | 2.9 [2.3, 3.7] | -0.3 | 0.4981 |
| Guyana 2009 | 8.6 [5.4, 13.6] | 7.1 [5.0, 10.0] | 1.5 | 0.5055 |
| Haiti 2016-17 | 6.9 [5.3, 9.0] | 5.2 [3.7, 7.3] | 1.7 | 0.1944 |
| Honduras 2011-12 | 3.0 [2.3, 4.0] | 2.0 [1.5, 2.8] | 1.0 | 0.0714 |
| Peru 2012 | 3.4 [2.6, 4.5] | 5.0 [3.8, 6.5] | -1.6 | 0.0480 |
| **SEARO** | **3.0 [1.7, 4.3]** | **2.3 [1.2, 3.5]** | **0.7** | **-** |
| India 2015-16 | 4.0 [3.6, 4.5] | 1.7 [1.6, 1.8] | 2.3 | 0.0000 |
| Maldives 2016-17 | 11.2 [6.6, 18.6] | 9.1 [7.2, 11.5] | 2.1 | 0.4711 |
| Myanmar 2015-16 | 3.6 [1.5, 8.3] | 1.5 [0.9, 2.5] | 2.1 | 0.0715 |
| Nepal 2016 | 2.6 [1.6, 4.2] | 1.0 [0.4, 2.8] | 1.6 | 0.0975 |
| Timor-Leste 2016 | 0.9 [0.4, 2.4] | 0.3 [0.1, 1.6] | 0.6 | 0.2498 |
| **WPRO** | **-** | **-** | **-** | **-** |
| Cambodia 2014 | 2.0 [0.9, 4.1] | 1.5 [0.9, 2.6] | 0.5 | 0.6084 |
| **Overall pooled prevalence** | **5.4 [4.8, 6.1]** | **3.4 [3.0, 3.9]** | **2.0** | **-** |

AFRO, African region; DRC, Democratic Republic of the Congo; STP, Sao Tome and Principe; EMRO, Eastern Mediterranean region; EURO, European region; PAHO, Americas region; SEARO, Southeast Asian region; WPRO, Western Pacific region.

*Values are percentages and 95% CIs; estimates account for survey design. Regional estimates are pooled prevalences and 95% CIs, calculated with the available data from countries within that region. The WPRO region only had one country with available data (Cambodia), thus the regional prevalence was not calculated.

† Gaps are expressed in percentage points, calculated as the arithmetic difference between the prevalence in urban vs. the prevalence in rural areas (%urban - %rural).

^‡^ p-values were obtained through chi-squared tests for each country. A p-value <0.05 indicate significant differences in the distribution of concurrent overweight/obesity and anaemia by area of residence.

^¶^ There is a missing estimate for concurrent overweight/obesity and anaemia among rural residents in Jordan, due to sample size <25.

**Supplemental Table 11.** Concurrent overweight/obesity and anaemia by household wealth quintiles among children (6-59 months).

|  | **Household wealth quintiles^*^** | | | | | | |
| --- | --- | --- | --- | --- | --- | --- | --- |
| **Country** | **Poorest (Q1)** | **Poorer (Q2)** | **Middle (Q3)** | **Richer (Q4)** | **Richest (Q5)** | **Gap**^†^ | **p-value**^‡^ |
| **AFRO** | **2.9 [2.4, 3.5]** | **3.2 [2.6, 3.8]** | **3.1 [2.6, 3.7]** | **2.9 [2.4, 3.4]** | **3.4 [2.8, 4.0]** | **0.5** | **-** |
| Angola 2015-16 | 1.7 [1.0, 2.7] | 2.2 [1.5, 3.1] | 2.8 [1.8, 4.4] | 2.4 [1.2, 4.8] | 2.5 [1.2, 5.1] | 0.8 | 0.7338 |
| Benin 2017-18 | 0.8 [0.4, 1.7] | 1.6 [1.0, 2.7] | 1.9 [1.2, 3.1] | 1.1 [0.6, 2.0] | 1.0 [0.6, 1.9] | 0.2 | 0.1803 |
| Burkina Faso 2010 | 2.6 [1.6, 4.2] | 3.4 [2.5, 4.7] | 2.9 [2.0, 4.2] | 3.1 [2.1, 4.6] | 2.3 [1.4, 3.8] | -0.3 | 0.7464 |
| Burundi 2016-17 | 1.3 [0.7, 2.2] | 1.0 [0.5, 1.9] | 1.1 [0.7, 2.0] | 1.0 [0.5, 1.8] | 0.7 [0.3, 1.4] | -0.6 | 0.8054 |
| Cameroon 2011 | 2.8 [1.9, 4.1] | 5.1 [3.7, 6.9] | 5.9 [4.2, 8.1] | 4.6 [3.3, 6.4] | 4.0 [2.7, 5.8] | 1.2 | 0.0470 |
| Congo 2011-12 | 2.0 [1.3, 3.2] | 2.4 [1.2, 4.8] | 2.9 [1.3, 6.3] | 2.8 [1.3, 5.7] | 4.1 [2.1, 7.8] | 2.1 | 0.6130 |
| Cote d'Ivoire 2011-12 | 3.0 [1.7, 5.3] | 3.2 [1.9, 5.1] | 2.5 [1.3, 4.8] | 2.3 [1.1, 4.7] | 3.3 [1.5, 7.2] | 0.3 | 0.9374 |
| DRC 2013-14 | 2.9 [2.0, 4.4] | 2.9 [1.9, 4.3] | 2.1 [1.4, 3.2] | 1.9 [1.2, 3.0] | 2.2 [1.4, 3.4] | -0.7 | 0.4430 |
| Eswatini 2006-07 | 6.9 [5.0, 9.6] | 7.6 [5.6, 10.3] | 7.9 [5.7, 10.8] | 6.0 [4.0, 8.8] | 6.3 [4.8, 8.1] | -0.6 | 0.8224 |
| Ethiopia 2016 | 1.3 [0.7, 2.3] | 0.6 [0.2, 1.4] | 0.9 [0.5, 1.8] | 2.6 [1.6, 4.3] | 1.8 [0.9, 3.5] | 0.5 | 0.0086 |
| Gabon 2012 | 5.5 [4.1, 7.3] | 5.9 [3.6, 9.7] | 3.9 [2.0, 7.3] | 6.3 [3.0, 12.6] | 9.0 [4.4, 17.8] | 3.5 | 0.3986 |
| Gambia 2013 | 1.3 [0.6, 2.8] | 1.2 [0.5, 2.8] | 2.3 [1.3, 3.9] | 1.6 [0.6, 4.6] | 1.9 [0.6, 5.5] | 0.6 | 0.7995 |
| Ghana 2014 | 1.9 [1.1, 3.2] | 2.2 [1.1, 4.1] | 2.0 [1.0, 4.2] | 1.3 [0.5, 3.3] | 2.2 [0.8, 5.8] | 0.3 | 0.9207 |
| Guinea 2018 | 4.6 [3.0, 6.9] | 3.8 [2.6, 5.6] | 5.2 [3.3, 8.2] | 2.9 [1.6, 5.3] | 7.8 [5.0, 11.8] | 3.2 | 0.0448 |
| Lesotho 2014 | 5.2 [3.2, 8.3] | 5.6 [3.1, 10.0] | 4.6 [2.1, 9.8] | 2.6 [1.1, 6.4] | 3.6 [1.3, 9.3] | -1.6 | 0.6676 |
| Madagascar 2003-04 | 2.2 [0.9, 5.4] | 5.8 [3.9, 8.5] | 2.2 [0.4, 10.9] | 1.9 [1.1, 3.1] | 3.6 [2.3, 5.5] | 1.4 | 0.2637 |
| Malawi 2015-16 | 2.6 [1.7, 4.0] | 4.6 [3.2, 6.6] | 3.9 [2.6, 5.7] | 2.6 [1.6, 4.2] | 2.7 [1.7, 4.5] | 0.1 | 0.1434 |
| Mali 2018 | 0.8 [0.3, 2.2] | 1.8 [0.9, 3.4] | 1.4 [0.7, 3.0] | 2.4 [1.5, 4.0] | 1.4 [0.7, 2.7] | 0.6 | 0.3346 |
| Mozambique 2011 | 5.5 [3.9, 7.7] | 6.6 [5.0, 8.7] | 6.6 [4.8, 9.1] | 6.0 [4.4, 8.1] | 5.5 [4.1, 7.4] | 0.0 | 0.8553 |
| Namibia 2013 | 1.0 [0.4, 2.9] | 2.4 [1.0, 5.7] | 2.4 [1.2, 4.9] | 2.8 [1.3, 5.9] | 4.2 [1.7, 10.2] | 3.2 | 0.3357 |
| Niger 2012 | 1.5 [0.8, 2.8] | 1.1 [0.4, 2.9] | 2.0 [1.0, 4.1] | 1.6 [0.9, 3.0] | 2.1 [1.2, 3.8] | 0.6 | 0.7206 |
| Nigeria 2018 | 1.7 [1.1, 2.6] | 2.2 [1.2, 3.0] | 1.4 [1.0, 2.0] | 1.6 [1.0, 2.6] | 0.6 [0.3, 1.1] | -1.1 | 0.0122 |
| Rwanda 2014-15 | 3.5 [2.3, 5.3] | 3.3 [2.2, 5.0] | 2.4 [1.3, 4.3] | 3.9 [2.6, 5.9] | 3.4 [2.0, 5.8] | -0.1 | 0.7526 |
| STP 2008-09 | 10.5 [7.0, 15.3] | 8.3 [5.2, 12.9] | 7.4 [4.5, 12.1] | 6.8 [3.7, 12.2] | 10.6 [6.2, 17.4] | 0.1 | 0.6277 |
| Senegal 2010-11 | 2.3 [1.5, 3.6] | 1.4 [0.8, 2.7] | 1.7 [0.9, 3.3] | 2.0 [0.7, 5.0] | 1.0 [0.2, 3.9] | -1.3 | 0.6865 |
| Sierra Leone 2013 | 11.0 [8.4, 14.2] | 7.5 [5.4, 10.3] | 6.7 [4.9, 9.1] | 7.7 [5.6, 10.4] | 6.0 [3.8, 9.3] | -5.0 | 0.0566 |
| South Africa 2016 | 13.1 [8.0, 20.8] | 10.6 [5.8, 18.7] | 7.7 [3.8, 14.9] | 4.6 [2.4, 8.9] | 5.8 [2.2, 14.2] | -7.3 | 0.1903 |
| Tanzania 2015-16 | 2.4 [1.8, 3.4] | 2.6 [1.7, 4.0] | 3.0 [2.1, 4.3] | 2.4 [1.7, 3.6] | 3.9 [2.8, 5.4] | 1.5 | 0.3236 |
| Togo 2013-14 | 1.4 [0.8, 2.6] | 1.5 [0.7, 3.0] | 2.1 [0.9, 4.5] | 1.3 [0.5, 3.4] | 2.0 [0.9, 4.3] | 0.6 | 0.8681 |
| Uganda 2016 | 3.3 [1.8, 5.8] | 4.0 [2.3, 6.7] | 3.9 [2.3, 6.6] | 4.1 [2.1, 7.9] | 12.8 [8.7, 18.5] | 9.5 | 0.8914 |
| Zimbabwe 2015 | 2.3 [1.4, 3.6] | 1.6 [0.8, 3.1] | 2.2 [1.3, 3.7] | 2.8 [1.8, 4.4] | 4.1 [2.6, 6.6] | 1.8 | 0.1008 |
| **EMRO** | **2.7 [1.1, 4.2]** | **2.5 [1.4, 3.5]** | **2.2 [1.2, 3.1]** | **2.8 [1.1, 4.6]** | **2.2 [1.2, 3.1]** | **-0.5** | **-** |
| Egypt 2014 | 5.8 [4.0, 8.5] | 4.3 [2.9, 6.3] | 3.6 [2.4, 5.3] | 5.1 [3.5, 7.4] | 4.2 [2.7, 6.5] | -1.6 | 0.4221 |
| Jordan 2012 | 2.2 [1.1, 4.5] | 1.7 [0.9, 3.5] | 0.9 [0.5, 1.5] | 0.4 [0.2, 0.8] | 1.7 [0.5, 5.6] | -0.5 | 0.1418 |
| Yemen 2013 | 1.4 [0.8, 2.7] | 2.0 [1.1, 3.4] | 2.1 [1.2, 3.7] | 3.5 [2.2, 5.6] | 1.0 [0.5, 2.2] | -0.4 | 0.0506 |
| **EURO** | **3.7 [2.2, 5.2]** | **4.2 [2.9, 5.5]** | **3.8 [2.3, 5.3]** | **3.3 [2.0, 4.6]** | **3.3 [1.5, 5.1]** | **-0.4** | **-** |
| Albania 2017-18 | 5.5 [3.8, 8.0] | 5.8 [3.5, 9.5] | 4.5 [2.5, 7.9] | 2.3 [1.2, 4.6] | 2.3 [0.4, 11.4] | -3.2 | 0.2873 |
| Armenia 2015-16 | 1.5 [0.5, 3.9] | 4.2 [2.1, 8.2] | 4.5 [2.3, 8.5] | 2.3 [1.0, 5.2] | 3.9 [1.7, 8.5] | 2.4 | 0.3759 |
| Azerbaijan 2006 | 4.2 [2.8, 6.1] | 6.0 [4.1, 8.6] | 6.3 [4.3, 9.1] | 4.8 [2.5, 9.1] | 4.7 [2.5, 8.6] | 0.5 | 0.8088 |
| Kyrgyz Republic 2012 | 5.2 [3.4, 7.8] | 4.5 [3.2, 6.4] | 4.6 [3.3, 6.3] | 5.7 [3.6, 8.8] | 5.1 [3.2, 8.0] | -0.1 | 0.9065 |
| Moldova 2005 | 2.8 [1.1, 6.7] | 3.8 [1.5, 9.5] | 2.3 [1.1, 4.5] | 2.3 [0.9, 5.3] | 4.4 [2.1, 9.0] | 1.6 | 0.7294 |
| Tajikistan 2017 | 1.4 [0.8, 2.3] | 1.9 [1.1, 3.2] | 2.0 [1.2, 3.3] | 1.8 [1.1, 2.8] | 1.1 [0.6, 2.2] | -0.3 | 0.5898 |
| **PAHO** | **2.1 [1.5, 2.8]** | **2.6 [1.6, 3.6]** | **2.5 [1.7, 3.4]** | **2.3 [1.5, 3.1]** | **2.3 [1.3, 3.2]** | **0.2** | **-** |
| Bolivia 2008 | 7.6 [5.4, 10.6] | 9.9 [6.7, 14.4] | 4.3 [2.6, 6.9] | 5.4 [3.2, 8.9] | 5.7 [3.1, 10.1] | -1.9 | 0.0488 |
| Guatemala 2014-15 | 1.5 [1.0, 2.2] | 1.3 [0.9, 1.8] | 1.1 [0.7, 1.8] | 1.0 [0.6, 1.6] | 1.0 [0.5, 1.9] | -0.5 | 0.6273 |
| Guyana 2009 | 1.8 [1.0, 3.5] | 1.6 [0.5, 4.6] | 4.8 [2.3, 9.6] | 4.7 [1.7, 12.2] | 1.8 [0.5, 5.9] | 0.0 | 0.1610 |
| Haiti 2016-17 | 2.2 [1.5, 3.3] | 2.4 [1.6, 3.5] | 2.9 [1.9, 4.5] | 1.8 [0.9, 3.3] | 2.4 [1.3, 4.5] | 0.2 | 0.7170 |
| Honduras 2011-12 | 1.4 [1.0, 2.0] | 1.0 [0.6, 1.7] | 2.0 [1.0, 4.0] | 1.2 [0.7, 2.1] | 0.8 [0.4, 1.5] | -0.6 | 0.2598 |
| Peru 2012 | 1.8 [1.3, 2.5] | 2.0 [1.4, 2.9] | 2.7 [1.9, 3.9] | 3.3 [2.1, 5.1] | 3.1 [1.6, 5.8] | 1.3 | 0.2448 |
| **SEARO** | **1.1 [0.6, 1.6]** | **1.1 [1.0, 1.1]** | **1.1 [0.7, 1.6]** | **1.0 [0.3, 1.7]** | **1.5 [0.6, 2.5]** | **0.4** | **-** |
| India 2015-16 | 0.9 [0.8, 1.0] | 0.9 [0.8, 1.0] | 1.2 [1.0, 1.4] | 1.4 [1.2, 1.6] | 1.7 [1.4, 2.0] | 0.8 | 0.0000 |
| Maldives 2016-17 | 2.2 [1.3, 3.7] | 1.6 [0.8, 3.2] | 1.5 [0.7, 3.1] | 3.1 [0.9, 10.0] | 0.0 [0.0, 0.0] | -2.2 | 0.3545 |
| Myanmar 2015-16 | 0.6 [0.3, 1.5] | 1.2 [0.4, 3.2] | 1.2 [0.5, 3.0] | 0.4 [0.1, 2.0] | 2.3 [1.0, 5.3] | 1.7 | 0.2026 |
| Nepal 2016 | 0.2 [0.0, 1.2] | 0.6 [0.2, 1.8] | 0.6 [0.1, 2.6] | 0.2 [0.0, 1.7] | 0.4 [0.1, 3.0] | 0.2 | 0.8155 |
| Timor-Leste 2016 | 2.5 [1.1, 5.5] | 0.4 [0.1, 1.9] | 1.1 [0.4, 3.3] | 1.5 [0.4, 6.1] | 2.7 [1.2, 6.1] | 0.2 | 0.3088 |
| **WPRO** | **-** | **-** | **-** | **-** | **-** | **-** | **-** |
| Cambodia 2014 | 0.8 [0.4, 1.8] | 2.2 [1.3, 3.8] | 1.0 [0.4, 2.3] | 1.3 [0.5, 3.3] | 1.7 [0.5, 5.3] | 0.9 | 0.4137 |
| **Overall pooled prevalence** | **2.6 [2.3, 2.9]** | **2.8 [2.5, 3.2]** | **2.7 [2.4, 3.0]** | **2.6 [2.2, 2.9]** | **2.8 [2.4, 3.1]** | **0.2** | **-** |

AFRO, African region; DRC, Democratic Republic of the Congo; STP, Sao Tome and Principe; EMRO, Eastern Mediterranean region; EURO, European region; PAHO, Americas region; SEARO, Southeast Asian region; WPRO, Western Pacific region.

*Values are percentages and 95% CIs; estimates account for survey design. Regional estimates are pooled prevalences and 95% CIs, calculated with the available data from countries within that region. The WPRO region only had one country with available data (Cambodia), thus the regional prevalence was not calculated.

† Gaps are expressed in percentage points, calculated as the arithmetic difference between the prevalence in the highest vs. the prevalence in the lowest household wealth quintile (%Q5 - %Q1).

^‡^ p-values were obtained through tests for trend for each country. A p-value <0.05 indicate significant differences in the distribution of concurrent overweight/obesity and anaemia by household wealth quintiles.

**Supplemental Table 12**. Concurrent overweight/obesity and anaemia by education level (maternal) among children (6-59 months).

|  | **Maternal education level^*^** | | | | | |
| --- | --- | --- | --- | --- | --- | --- |
| **Country** | **None (E1)** | **Primary (E2)** | **Secondary (E3)** | **Higher (E4)** | **Gap**^†^ | **p-value**^‡^ |
| **AFRO** | **2.8 [2.3, 3.4]** | **3.0 [2.5, 3.6]** | **3.1 [2.5, 3.7]** | **3.5 [2.3, 4.7]** | **0.7** | **-** |
| Angola 2015-16 | 2.4 [1.6, 3.5] | 2.6 [1.8, 3.7] | 2.0 [1.2, 3.2] | 1.7 [0.2, 12.1] | -0.7 | 0.8348 |
| Benin 2017-18 | 1.2 [0.9, 1.7] | 1.2 [0.7, 2.1] | 1.6 [0.9, 2.9] | 3.5 [1.9, 6.6] | 2.3 | 0.4241 |
| Burkina Faso 2010^¶^ | 2.9 [2.3, 3.6] | 3.3 [2.1, 5.4] | 2.4 [1.0, 5.4] | - | - | - |
| Burundi 2016-17 | 0.9 [0.6, 1.4] | 1.3 [0.9, 1.9] | 0.5 [0.2, 1.4] | 0.0 [0.0, 0.0] | -0.9 | 0.4037 |
| Cameroon 2011 | 2.3 [1.4, 3.7] | 5.1 [4.1, 6.2] | 5.4 [4.1, 7.1] | 5.6 [2.7, 11.1] | 3.3 | 0.0046 |
| Congo 2011-12 | 2.1 [0.6, 7.5] | 2.8 [1.8, 4.3] | 2.8 [1.8, 4.2] | 2.6 [0.5, 13.0] | 0.5 | 0.9732 |
| Cote d'Ivoire 2011-12^¶^ | 3.1 [2.2, 4.4] | 2.6 [1.5, 4.4] | 1.4 [0.3, 6.5] | - | - | - |
| DRC 2013-14 | 2.4 [1.5, 3.7] | 2.3 [1.7, 3.1] | 2.8 [2.0, 3.7] | 0.0 [0.0, 0.0] | -2.4 | 0.6201 |
| Eswatini 2006-07 | 4.7 [2.6, 8.2] | 7.5 [5.7, 10.0] | 7.0 [5.4, 9.1] | 6.9 [3.9, 11.9] | 2.2 | 0.6856 |
| Ethiopia 2016 | 1.1 [0.7, 1.6] | 1.9 [1.2, 3.1] | 0.9 [0.4, 2.0] | 3.2 [0.6, 15.1] | 2.1 | 0.0932 |
| Gabon 2012 | 7.7 [3.0, 18.4] | 5.2 [3.7, 7.3] | 6.1 [4.2, 8.9] | 5.8 [1.5, 20.2] | -1.9 | 0.8773 |
| Gambia 2013 | 1.2 [0.7, 1.9] | 1.7 [0.7, 3.9] | 2.7 [1.2, 5.8] | 2.1 [1.1, 3.9] | 0.9 | 0.1817 |
| Ghana 2014 | 1.8 [1.1, 3.1] | 1.7 [0.9, 3.4] | 2.1 [1.2, 3.6] | 1.7 [0.2, 12.1] | -0.1 | 0.9576 |
| Guinea 2018 | 4.0 [3.1, 5.1] | 6.3 [3.7, 10.5] | 8.2 [4.7, 13.8] | 6.1 [2.2, 15.6] | 2.1 | 0.0260 |
| Lesotho 2014^¶^ | - | 3.6 [2.4, 5.5] | 4.4 [2.6, 7.2] | 9.4 [3.7, 21.8] | - | - |
| Madagascar 2003-04 | 3.5 [2.1, 5.9] | 2.9 [1.5, 5.6] | 2.8 [2.0, 4.0] | 11.4 [2.9, 35.7] | 7.9 | 0.4658 |
| Malawi 2015-16 | 3.5 [2.1, 5.8] | 3.5 [2.8, 4.3] | 3.1 [1.9, 4.9] | 0.7 [0.4, 1.3] | -2.8 | 0.5332 |
| Mali 2018 | 1.7 [1.2, 2.4] | 1.0 [0.4, 3.0] | 1.4 [0.6, 2.9] | 0.9 [0.6, 1.2] | -0.8 | 0.6340 |
| Mozambique 2011 | 7.0 [5.6, 8.7] | 5.7 [4.6, 7.0] | 5.1 [3.4, 7.7] | 0.0 [0.0, 0.0] | -7.0 | 0.2380 |
| Namibia 2013 | 1.5 [0.3, 5.9] | 2.1 [1.0, 4.2] | 2.4 [1.5, 3.9] | 3.9 [1.1, 13.0] | 2.4 | 0.7393 |
| Niger 2012 | 1.7 [1.2, 2.4] | 1.3 [0.6, 3.2] | 1.8 [0.6, 5.3] | 3.3 [0.5, 20.2] | 1.6 | 0.8513 |
| Nigeria 2018 | 1.8 [1.4, 2.4] | 1.5 [0.9, 2.3] | 1.5 [1.1, 2.0] | 0.3 [0.1, 1.2] | -1.5 | 0.0259 |
| Rwanda 2014-15 | 3.8 [2.3, 6.0] | 3.2 [2.5, 4.1] | 3.8 [2.1, 6.7] | 1.1 [0.1, 8.1] | -2.7 | 0.6448 |
| STP 2008-09^¶^ | 11.8 [5.2, 24.6] | 8.3 [6.5, 10.5] | 8.9 [5.8, 13.6] | - | - | - |
| Senegal 2010-11^¶^ | 2.1 [1.5, 3.0] | 0.6 [0.2, 1.5] | 1.4 [0.2, 9.4] | - | - | - |
| Sierra Leone 2013 | 8.4 [7.0, 9.9] | 5.5 [3.4, 9.0] | 7.3 [5.0, 10.5] | 21.4 [8.5, 44.4] | 13.0 | 0.0278 |
| South Africa 2016^¶^ | - | 17.5 [8.2, 33.4] | 9.1 [6.6, 12.4] | 1.7 [0.4, 7.4] | - | - |
| Tanzania 2015-16 | 2.8 [2.0, 3.9] | 2.6 [2.1, 3.2] | 3.8 [2.6, 5.5] | 6.0 [1.5, 20.6] | 3.2 | 0.2491 |
| Togo 2013-14 | 1.4 [0.8, 2.3] | 1.9 [1.1, 3.3] | 1.7 [0.8, 3.3] | 2.2 [0.3, 16.4] | 0.8 | 0.7938 |
| Uganda 2016 | 5.4 [3.0, 9.7] | 4.5 [3.4, 6.1] | 8.9 [5.1, 15.0] | 6.4 [3.3, 12.0] | 1.0 | 0.7791 |
| Zimbabwe 2015 | 4.9 [0.7, 26.7] | 2.1 [1.4, 3.3] | 2.5 [1.9, 3.3] | 4.6 [2.1, 9.9] | -0.3 | 0.3406 |
| **EMRO** | **3.3 [2.1, 4.5]** | **3.3 [2.1, 4.6]** | **1.9 [1.5, 2.2]** | **2.2 [1.6, 2.8]** | **-1.1** | **-** |
| Egypt 2014 | 3.1 [2.0, 4.9] | 5.9 [3.6, 9.5] | 4.8 [3.7, 6.2] | 4.5 [2.9, 6.8] | 1.4 | 0.3038 |
| Jordan 2012 | 1.8 [0.5, 6.6] | 1.4 [0.6, 3.4] | 1.5 [0.8, 2.5] | 1.3 [0.7, 2.1] | -0.5 | 0.9362 |
| Yemen 2013^§^ | - | - | - | - | - | - |
| **EURO** | **-** | **-** | **-** | **-** | **-** | **-** |
| Albania 2017-18^¶^ | - | 5.9 [4.2, 8.2] | 3.5 [2.1, 5.6] | 2.1 [1.1, 3.8] | - | - |
| Armenia 2015-16^¶^ | - | 2.2 [0.5, 9.7] | 3.3 [1.9, 5.7] | 3.4 [2.0, 5.7] | - | - |
| Azerbaijan 2006^¶^ | - | 0.0 [0.0, 0.0] | 5.3 [4.1, 6.9] | 3.8 [1.8, 7.9] | - | - |
| Kyrgyz Republic 2012^¶^ | - | - | 5.4 [4.3, 6.7] | 4.6 [3.5, 6.0] | - | - |
| Moldova 2005^¶^ | - | - | 3.1 [2.0, 4.7] | 3.3 [2.1, 5.3] | - | - |
| Tajikistan 2017 | 1.0 [0.2, 3.9] | 1.8 [0.7, 4.3] | 1.7 [1.4, 2.3] | 1.4 [0.7, 2.7] | 0.4 | 0.8131 |
| **PAHO** | **1.8 [1.2, 2.4]** | **2.7 [1.8, 3.5]** | **2.4 [1.6, 3.2]** | **2.1 [1.1, 3.2]** | **0.3** | **-** |
| Bolivia 2008 | 3.4 [1.4, 8.1] | 7.9 [6.1, 10.1] | 5.8 [4.1, 8.3] | 4.7 [2.6, 8.5] | 1.3 | 0.1066 |
| Guatemala 2014-15 | 1.4 [0.9, 2.2] | 1.2 [1.0, 1.6] | 1.1 [0.7, 1.7] | 1.1 [0.3, 3.9] | -0.3 | 0.8791 |
| Guyana 2009 | 1.3 [0.2, 9.7] | 1.3 [0.6, 2.9] | 3.1 [2.0, 4.9] | 4.7 [0.6, 28.9] | 3.4 | 0.3931 |
| Haiti 2016-17 | 2.7 [1.8, 4.1] | 2.5 [1.9, 3.4] | 2.0 [1.3, 2.9] | 1.8 [0.3, 9.3] | -0.9 | 0.6602 |
| Honduras 2011-12 | 1.2 [0.5, 3.1] | 1.5 [1.1, 2.2] | 1.0 [0.7, 1.6] | 1.0 [0.5, 2.0] | -0.2 | 0.4465 |
| Peru 2012 | 1.1 [0.3, 3.8] | 2.4 [1.7, 3.4] | 2.6 [2.0, 3.4] | 2.6 [1.6, 4.0] | 1.5 | 0.6896 |
| **SEARO** | **1.1 [0.5, 1.7]** | **1.0 [0.7, 1.3]** | **1.3 [0.9, 1.6]** | **1.9 [1.7, 2.1]** | **0.8** | **-** |
| India 2015-16 | 0.9 [0.8, 1.0] | 1.0 [0.9, 1.2] | 1.2 [1.1, 1.3] | 1.7 [1.4, 2.1] | 0.8 | 0.0000 |
| Maldives 2016-17 | 2.9 [0.3, 23.1] | 1.5 [0.7, 3.2] | 2.0 [1.1, 3.6] | 1.4 [0.7, 2.8] | -1.5 | 0.7530 |
| Myanmar 2015-16 | 0.2 [0.0, 0.6] | 0.9 [0.5, 1.7] | 1.4 [0.7, 3.1] | 2.3 [0.9, 5.8] | 2.1 | 0.1010 |
| Nepal 2016 | 0.0 [0.0, 0.0] | 0.3 [0.1, 1.6] | 0.7 [0.3, 2.0] | 1.2 [0.4, 3.7] | 1.2 | 0.0612 |
| Timor-Leste 2016 | 2.5 [1.3, 5.1] | 0.5 [0.1, 2.1] | 1.5 [0.7, 3.2] | 2.6 [0.7, 9.6] | 0.1 | 0.2660 |
| **WPRO** | **-** | **-** | **-** | **-** | **-** | **-** |
| Cambodia 2014 | 1.8 [0.9, 3.6] | 1.4 [0.9, 2.2] | 0.9 [0.4, 2.1] | 3.4 [0.9, 12.4] | 1.6 | 0.2421 |
| **Overall pooled prevalence** | **2.4 [2.1, 2.8]** | **2.6 [2.3, 3.0]** | **2.5 [2.2, 2.8]** | **2.3 [1.8, 2.8]** | **-0.1** | **-** |

AFRO, African region; DRC, Democratic Republic of the Congo; STP, Sao Tome and Principe; EMRO, Eastern Mediterranean region; EURO, European region; PAHO, Americas region; SEARO, Southeast Asian region; WPRO, Western Pacific region.

*Values are percentages and 95% CIs; estimates account for survey design. Regional estimates are pooled prevalences and 95% CIs, calculated with the available data from countries within that region. The WPRO region only had one country with available data (Cambodia), thus the regional prevalence was not calculated.

† Gaps are expressed in percentage points, calculated as the arithmetic difference between the prevalence in the highest vs. the prevalence in the lowest maternal education level (%E4 - %E1).

^‡^ p-values were obtained through tests for trend for each country. A p-value <0.05 indicate significant differences in the distribution of concurrent overweight/obesity and anaemia by maternal education level.

^§^ Yemen has missing data on education level, and thus, the stratified estimates could not be calculated.

^¶^ Missing estimates for certain categories are missing for Burkina Faso, Cote d’Ivoire, Lesotho, Sao Tome and Principe, Senegal, South Africa, Albania, Armenia, Azerbaijan, Kyrgyz Republic, Moldova due to sample size <25. These countries were excluded for the calculation of pooled estimates and, as a result, the pooled prevalence for the European region could not be calculated.

**Supplemental Table 13**. Concurrent overweight/obesity and anaemia by area of residence among children (6-59 months).

|  | **Area of residence^*^** | | | |
| --- | --- | --- | --- | --- |
| **Country** | **Urban** | **Rural** | **Gap**^†^ | **p-value**^‡^ |
| **AFRO** | **3.5 [2.9, 4.0]** | **3.3 [2.3, 3.8]** | **0.2** | **-** |
| Angola 2015-16 | 2.6 [1.9, 3.6] | 1.9 [1.4, 2.6] | 0.7 | 0.1620 |
| Benin 2017-18 | 1.0 [0.7, 1.5] | 1.5 [1.1, 2.0] | -0.5 | 0.1406 |
| Burkina Faso 2010 | 3.2 [2.1, 5.0] | 2.8 [2.3, 3.6] | 0.4 | 0.5996 |
| Burundi 2016-17 | 1.0 [0.5, 2.2] | 1.0 [0.8, 1.4] | 0.0 | 0.9843 |
| Cameroon 2011 | 4.7 [3.6, 5.9] | 4.3 [3.5, 5.3] | 0.4 | 0.6330 |
| Congo 2011-12 | 3.1 [2.0, 4.8] | 2.1 [ 1.6, 2.8] | 1.0 | 0.1416 |
| Cote d'Ivoire 2011-12 | 2.6 [1.5, 4.3] | 3.0 [2.3, 4.1] | -0.4 | 0.5649 |
| DRC 2013-14 | 2.1 [1.5, 2.8] | 2.6 [2.0, 3.3] | -0.5 | 0.2598 |
| Eswatini 2006-07 | 10.3 [7.9, 13.4] | 6.3 [5.2, 7.6] | 4.0 | 0.0027 |
| Ethiopia 2016 | 1.8 [0.9, 3.4] | 1.3 [0.9, 1.8] | 0.5 | 0.4117 |
| Gabon 2012 | 6.0 [4.3, 8.2] | 5.9 [4.5, 7.9] | 0.1 | 0.9697 |
| Gambia 2013 | 1.7 [0.9, 3.3] | 1.5 [1.0, 2.4] | 0.2 | 0.8259 |
| Ghana 2014 | 2.1 [1.3, 3.6] | 1.8 [1.1, 2.8] | 0.3 | 0.5669 |
| Guinea 2018 | 5.8 [3.8, 8.8] | 4.3 [3.3, 5.5] | 1.5 | 0.2165 |
| Lesotho 2014 | 3.1 [1.2, 7.7] | 4.9 [3.6, 6.6] | -1.8 | 0.3516 |
| Madagascar 2003-04 | 3.4 [2.1, 5.2] | 3.1 [1.7, 5.6] | 0.3 | 0.8039 |
| Malawi 2015-16 | 1.8 [0.9, 3.3] | 3.6 [2.9, 4.4] | -1.8 | 0.0334 |
| Mali 2018 | 2.0 [1.2, 3.2] | 1.5 [1.0, 2.1] | 0.5 | 0.3149 |
| Mozambique 2011 | 5.9 [4.6, 7.6] | 6.1 [5.1, 7.2] | -0.2 | 0.8329 |
| Namibia 2013 | 2.8 [1.6, 5.0] | 2.0 [1.3, 3.2] | 0.8 | 0.3636 |
| Niger 2012 | 2.5 [1.4, 4.7] | 1.5 [1.0, 2.2] | 1.0 | 0.1593 |
| Nigeria 2018 | 1.2 [0.8, 1.7] | 1.7 [1.4, 2.1] | -0.5 | 0.1013 |
| Rwanda 2014-15 | 3.4 [2.0, 5.7] | 3.3 [2.6, 4.1] | 0.1 | 0.8813 |
| STP 2008-09 | 7.9 [5.6, 11.0] | 9.4 [7.2, 12.0] | -1.5 | 0.4153 |
| Senegal 2010-11 | 1.8 [0.9, 3.8] | 1.6 [1.1, 2.4] | 0.2 | 0.7705 |
| Sierra Leone 2013 | 7.5 [5.6, 10.0] | 8.1 [6.7, 9.8] | -0.6 | 0.6699 |
| South Africa 2016 | 8.1 [4.9, 13.0] | 10.3 [7.3, 14.4] | -2.2 | 0.4166 |
| Tanzania 2015-16 | 3.1 [2.2, 4.2] | 2.8 [2.3, 3.3] | 0.3 | 0.5621 |
| Togo 2013-14 | 1.8 [1.0, 3.2] | 1.6 [1.0, 2.4] | 0.2 | 0.7835 |
| Uganda 2016 | 9.2 [5.6, 14.6] | 4.7 [3.6, 6.1] | 4.5 | 0.8565 |
| Zimbabwe 2015 | 3.5 [2.4, 5.0] | 2.1 [1.6, 2.9] | 1.4 | 0.0447 |
| **EMRO** | **2.3 [0.8, 3.7]** | **2.9 [1.8, 3.9]** | **-0.6** | **-** |
| Egypt 2014 | 4.6 [3.4, 6.3] | 4.5 [3.6, 5.7] | 0.1 | 0.9037 |
| Jordan 2012 | 1.2 [0.7, 2.0] | 2.4 [1.5, 3.8] | -1.2 | 0.0379 |
| Yemen 2013 | 1.8 [1.0, 3.1] | 2.1 [1.5, 2.8] | -0.3 | 0.6208 |
| **EURO** | **3.3 [1.8, 4.7]** | **4.2 [2.7, 5.6]** | **-0.9** | **-** |
| Albania 2017-18 | 3.0 [1.8, 5.2] | 5.8 [4.3, 7.6] | -2.8 | 0.0357 |
| Armenia 2015-16 | 3.0 [1.8, 5.1] | 3.6 [2.3, 5.7] | -0.6 | 0.5802 |
| Azerbaijan 2006 | 5.9 [3.9, 8.9] | 4.5 [3.6, 5.7] | 1.4 | 0.2761 |
| Kyrgyz Republic 2012 | 5.1 [3.6, 7.1] | 5.0 [4.1, 6.1] | 0.1 | 0.9439 |
| Moldova 2005 | 2.8 [1.5, 5.0] | 3.2 [1.9, 5.5] | -0.4 | 0.7096 |
| Tajikistan 2017 | 1.3 [0.8, 2.0] | 1.8 [1.4, 2.3] | -0.5 | 0.1823 |
| **PAHO** | **2.4 [1.6, 3.2]** | **2.7 [1.9, 3.5]** | **-0.3** | **-** |
| Bolivia 2008 | 5.4 [3.9, 7.3] | 8.4 [6.5, 10.7] | -3.0 | 0.0262 |
| Guatemala 2014-15 | 0.9 [0.7, 1.3] | 1.4 [1.1, 1.8] | -0.5 | 0.0670 |
| Guyana 2009 | 1.7 [0.6, 4.4] | 3.0 [1.9, 4.7] | -1.3 | 0.2666 |
| Haiti 2016-17 | 1.8 [1.2, 2.7] | 2.6 [2.1, 3.3] | -0.8 | 0.1089 |
| Honduras 2011-12 | 1.4 [0.9, 2.4] | 1.3 [1.0, 1.6] | 0.1 | 0.6263 |
| Peru 2012 | 2.7 [2.1, 3.4] | 2.1 [1.6, 2.7] | 0.6 | 0.1414 |
| **SEARO** | **1.2 [0.6, 1.9]** | **1.2 [0.8, 1.6]** | **0.0** | **-** |
| India 2015-16 | 1.4 [1.2, 1.6] | 1.1 [1.0, 1.1] | 0.3 | 0.0011 |
| Maldives 2016-17 | 1.8 [0.4, 6.9] | 1.8 [1.3, 2.6] | 0.0 | 0.9674 |
| Myanmar 2015-16 | 1.8 [1.0, 3.5] | 0.8 [0.4, 1.5] | 1.0 | 0.0659 |
| Nepal 2016 | 0.4 [0.2, 1.0] | 0.5 [0.2, 1.3] | -0.1 | 0.6708 |
| Timor-Leste 2016 | 1.1 [0.4, 3.2] | 1.9 [1.1, 3.1] | -0.8 | 0.4099 |
| **WPRO** | **-** | **-** | **-** | **-** |
| Cambodia 2014 | 1.3 [0.5, 3.1] | 1.4 [0.9, 2.1] | -0.1 | 0.8734 |
| **Overall pooled prevalence** | **2.9 [2.5, 3.2]** | **3.0 [2.7, 3.3]** | **-0.1** | **-** |

AFRO, African region; DRC, Democratic Republic of the Congo; STP, Sao Tome and Principe; EMRO, Eastern Mediterranean region; EURO, European region; PAHO, Americas region; SEARO, Southeast Asian region; WPRO, Western Pacific region.

*Values are percentages and 95% CIs; estimates account for survey design. Regional estimates are pooled prevalences and 95% CIs, calculated with the available data from countries within that region. The WPRO region only had one country with available data (Cambodia), thus the regional prevalence was not calculated.

† Gaps are expressed in percentage points, calculated as the arithmetic difference between the prevalence in urban vs. the prevalence in rural areas (%urban - %rural).

^‡^ p-values were obtained through chi-squared tests for each country. A p-value <0.05 indicate significant differences in the distribution of concurrent overweight/obesity and anaemia by area of residence.

**Supplemental Table 14**. Concurrent overweight/obesity and anaemia by sex among children (6-59 months).

|  | **Sex^*^** | | | |
| --- | --- | --- | --- | --- |
| **Country** | **Girls** | **Boys** | **Gap**^†^ | **p-value**^‡^ |
| **AFRO** | **2.9 [2.4, 3.3]** | **3.5 [3.0, 4.1]** | **0.6** | **-** |
| Angola 2015-16 | 1.8 [1.2, 2.7] | 2.8 [2.1, 3.7] | 1.0 | 0.0797 |
| Benin 2017-18 | 1.3 [0.9, 1.9] | 1.3 [0.9, 1.9] | 0.0 | 0.9824 |
| Burkina Faso 2010 | 3.0 [2.4, 3.9] | 2.8 [2.1, 3.7] | -0.2 | 0.6094 |
| Burundi 2016-17 | 0.8 [0.5, 1.3] | 1.2 [0.9, 1.7] | 0.4 | 0.1388 |
| Cameroon 2011 | 4.0 [3.2, 5.1] | 4.9 [4.0, 6.0] | 0.9 | 0.1976 |
| Congo 2011-12 | 2.7 [1.8, 4.0] | 2.8 [1.9, 4.2] | 0.1 | 0.8347 |
| Cote d'Ivoire 2011-12 | 2.9 [1.9, 4.3] | 2.9 [1.9, 4.2] | 0.0 | 0.9998 |
| DRC 2013-14 | 2.4 [1.8, 3.2] | 2.5 [1.9, 3.29] | 0.1 | 0.7930 |
| Eswatini 2006-07 | 5.6 [4.3, 7.3] | 8.5 [6.9, 10.3] | 2.9 | 0.0244 |
| Ethiopia 2016 | 1.2 [0.7, 1.9] | 1.5 [1.1, 2.2] | 0.3 | 0.3820 |
| Gabon 2012 | 5.2 [3.6, 7.3] | 6.7 [4.7, 9.6] | 1.5 | 0.2787 |
| Gambia 2013 | 1.7 [1.0, 2.8] | 1.6 [0.9, 2.7] | -0.1 | 0.8389 |
| Ghana 2014 | 1.3 [0.7, 2.2] | 2.5 [1.6, 3.8] | 1.2 | 0.0342 |
| Guinea 2018 | 3.8 [2.9, 5.2] | 5.5 [4.1, 7.5] | 1.7 | 0.0869 |
| Lesotho 2014 | 3.5 [2.0, 5.9] | 5.4 [3.8, 7.7] | 1.9 | 0.1651 |
| Madagascar 2003-04 | 3.2 [1.5, 6.6] | 3.0 [1.8, 5.0] | -0.2 | 0.8804 |
| Malawi 2015-16 | 2.8 [2.1, 3.8] | 3.9 [3.1, 5.0] | 1.1 | 0.0898 |
| Mali 2018 | 1.8 [1.2, 2.7] | 1.4 [0.9, 2.1] | -0.4 | 0.3725 |
| Mozambique 2011 | 5.4 [4.4, 6.6] | 6.8 [5.6, 8.2] | 1.4 | 0.1189 |
| Namibia 2013 | 2.2 [1.3, 3.7] | 2.5 [1.5, 4.4] | 0.3 | 0.7012 |
| Niger 2012 | 1.7 [1.1, 2.7] | 1.6 [1.1, 2.4] | -0.1 | 0.8366 |
| Nigeria 2018 | 1.1 [0.8, 1.5] | 1.8 [1.4, 2.4] | 0.7 | 0.0186 |
| Rwanda 2014-15 | 2.7 [1.9, 3.9] | 3.9 [3.0, 4.9] | 1.2 | 0.1098 |
| STP 2008-09 | 9.0 [6.6, 12.1] | 8.1 [5.7, 11.5] | -0.9 | 0.6772 |
| Senegal 2010-11 | 2.0 [1.2, 3.4] | 1.4 [0.9, 2.1] | -0.6 | 0.2610 |
| Sierra Leone 2013 | 7.5 [6.2, 9.2] | 8.4 [6.8, 10.3] | 0.9 | 0.4000 |
| South Africa 2016 | 8.6 [5.5, 13.3] | 9.5 [6.3, 14.1] | 0.9 | 0.7458 |
| Tanzania 2015-16 | 2.3 [1.8, 2.9] | 3.4 [2.7, 4.1] | 1.1 | 0.0179 |
| Togo 2013-14 | 1.2 [0.7, 2.1] | 2.1 [1.3, 3.2] | 0.9 | 0.1124 |
| Uganda 2016 | 1.8 [1.2, 2.6] | 3.5 [2.5, 4.7] | 1.7 | 0.0085 |
| Zimbabwe 2015 | 1.9 [1.4, 2.7] | 3.1 [2.3, 4.3] | 1.2 | 0.0440 |
| **EMRO** | **2.2 [0.9, 3.4]** | **2.9 [1.7, 4.0]** | **0.7** | **-** |
| Egypt 2014 | 4.2 [3.3, 5.5] | 4.8 [3.8, 6.2] | 0.6 | 0.4506 |
| Jordan 2012 | 1.2 [0.6, 2.4] | 1.6 [1.0, 2.5] | 0.4 | 0.5087 |
| Yemen 2013 | 1.5 [0.9, 2.3] | 2.5 [1.8, 3.5] | 1.0 | 0.0756 |
| **EURO** | **3.4 [2.1, 4.8]** | **4.2 [2.6, 5.7]** | **0.8** | **-** |
| Albania 2017-18 | 3.1 [2.1, 4.5] | 5.5 [3.8, 7.8] | 2.4 | 0.0289 |
| Armenia 2015-16 | 3.2 [1.9, 5.4] | 3.4 [2.1, 5.4] | 0.2 | 0.9074 |
| Azerbaijan 2006 | 4.5 [3.2, 6.2] | 5.9 [4.3, 8.1] | 1.4 | 0.1937 |
| Kyrgyz Republic 2012 | 4.8 [3.7, 6.2] | 5.2 [4.2, 6.5] | 0.4 | 0.5862 |
| Moldova 2005 | 3.8 [2.4, 6.0] | 2.4 [1.4, 4.2] | -1.4 | 0.2533 |
| Tajikistan 2017 | 1.4 [1.0, 2.1] | 1.9 [1.4, 2.6] | 0.5 | 0.1857 |
| **PAHO** | **2.3 [1.6, 3.0]** | **2.8 [1.9, 3.7]** | **0.5** | **-** |
| Bolivia 2008 | 5.3 [3.8, 7.2] | 8.1 [6.3, 10.4] | 2.8 | 0.0354 |
| Guatemala 2014-15 | 1.0 [0.7, 1.3] | 1.5 [1.1, 1.9] | 0.5 | 0.0373 |
| Guyana 2009 | 3.7 [2.2, 6.2] | 1.8 [0.9, 3.4] | -1.9 | 0.0929 |
| Haiti 2016-17 | 1.7 [1.2, 2.4] | 2.9 [2.3, 3.8] | 1.2 | 0.0115 |
| Honduras 2011-12 | 1.4 [1.0, 2.1] | 1.3 [0.9, 1.7] | -0.1 | 0.5264 |
| Peru 2012 | 2.5 [1.9, 3.3] | 2.5 [1.9, 3.2] | 0.0 | 0.9669 |
| **SEARO** | **1.0 [0.4, 1.6]** | **1.3 [0.9, 1.7]** | **0.3** | **-** |
| India 2015-16 | 1.2 [1.1, 1.3] | 1.2 [1.1, 1.3] | 0.0 | 0.8269 |
| Maldives 2016-17 | 1.0 [0.6, 1.7] | 2.5 [1.4, 4.3] | 1.5 | 0.0164 |
| Myanmar 2015-16 | 0.6 [0.3, 1.3] | 1.4 [0.8, 2.4] | 0.8 | 0.0527 |
| Nepal 2016 | 0.2 [0.0, 1.3] | 0.6 [0.3, 1.3] | 0.4 | 0.2516 |
| Timor-Leste 2016 | 1.9 [1.0, 3.4] | 1.4 [0.8, 2.7] | -0.5 | 0.4839 |
| **WPRO** | **-** | **-** | **-** | **-** |
| Cambodia 2014 | 1.0 [0.5, 1.9] | 1.7 [1.2, 2.6] | 0.7 | 0.0893 |
| **Overall pooled prevalence** | **2.5 [2.2, 2.8]** | **3.2 [2.8, 3.5]** | 0.7 | **-** |

AFRO, African region; DRC, Democratic Republic of the Congo; STP, Sao Tome and Principe; EMRO, Eastern Mediterranean region; EURO, European region; PAHO, Americas region; SEARO, Southeast Asian region; WPRO, Western Pacific region.

*Values are percentages and 95% CIs; estimates account for survey design. Regional estimates are pooled prevalences and 95% CIs, calculated with the available data from countries within that region. The WPRO region only had one country with available data (Cambodia), thus the regional prevalence was not calculated.

† Gaps are expressed in percentage points, calculated as the arithmetic difference between the prevalence in boys vs. the prevalence in girls (%boys - %girls).

^‡^ p-values were obtained through chi-squared for each country. A p-value <0.05 indicate significant differences in the distribution of concurrent overweight/obesity and anaemia by sex.
